# Metabolic stress–activated endothelial STING rewires the sinusoidal immune niche to promote MASLD progression

**DOI:** 10.1172/JCI207031

**Published:** 2026-08-06

**Authors:** Zhi-Bin Lin, Peng Zou, Xian-Yi Ma, Jun-Bo Song, Hong Zhang, Wei Du, Dan Wei, Ping Song, Xin Hong, Jing-Jing Liu, Zhi-Qiang Fang, Hao Xu, Fei He, Juan-Li Duan, Ke-Feng Dou, Lin Wang

**Affiliations:** Department of Hepatobiliary Surgery, Xijing Hospital, Fourth Military Medical University, Xi’an, China.

**Keywords:** Hepatology, Metabolism, Fibrosis, Innate immunity, Molecular biology

## Abstract

Liver sinusoidal endothelial cells (LSECs) regulate nutrient flux and immune surveillance within the hepatic niche, yet how they function as metabolic stress sensors that instruct adaptive immune remodeling during metabolic dysfunction–associated steatotic liver disease (MASLD) remains unclear. Here, single-nucleus transcriptomics of human MASLD revealed stage-dependent activation of cyclic GMP-AMP synthase/stimulator of interferon genes (cGAS/STING) signaling in LSEC comparable with that in macrophages, with endothelial activation showing greater responsiveness to metabolic stress. Endothelium-specific STING deletion attenuated steatohepatitis and fibrosis in mice. Mechanistically, LSEC-intrinsic STING activation reprogrammed the angiocrine landscape through NF-κB–mediated transcriptional repression of the endothelium-derived factor BMP4. Loss of BMP4 disrupted the tolerance-supporting sinusoidal immunometabolic niche, skewing CD4^+^ T cell differentiation toward pathogenic Th17 states while destabilizing Treg, collectively exacerbating hepatic metabolic failure. In human MASLD, endothelial STING activity inversely correlated with BMP4 expression at single-cell resolution. Targeted delivery of a STING inhibitor to LSECs using peptide-functionalized nanoparticles restored hepatic immunometabolic balance at one-tenth the systemic dose. Together, these findings establish endothelial STING as a metabolically responsive vascular immune checkpoint that links chronic metabolic stress to adaptive immune remodeling and fibrotic progression.

## Abstract 2

<strong>Endothelial STING skews hepatic CD4⁺ T cell differentiation toward pathogenic Th17 while destabilizing Treg, thereby promoting MASLD progression</strong>

## Graphical abstract

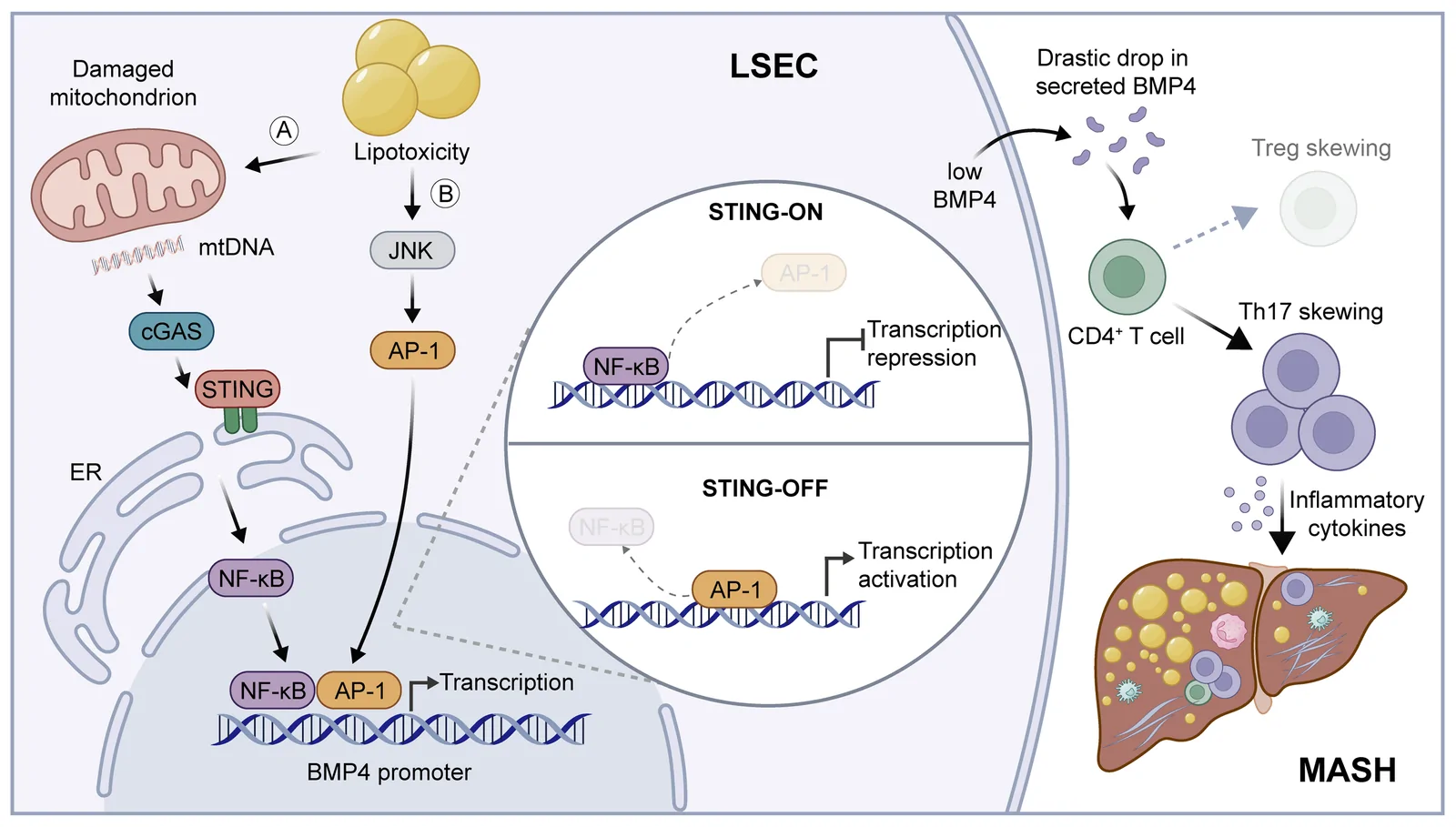

## Introduction

Metabolic dysfunction–associated steatotic liver disease (MASLD) affects nearly one-third of the global population and exemplifies the pathological consequences of chronic nutrient excess (1). While hepatocellular lipid accumulation initiates metabolic stress, progression to steatohepatitis and fibrosis reflects a broader collapse of hepatic immunometabolic homeostasis (2). Increasing evidence indicates that this transition is driven not solely by hepatocyte injury, but by maladaptive signaling within the sinusoidal niche that coordinates metabolic and immune responses (3).

Liver sinusoidal endothelial cells (LSECs) form the primary vascular interface of the liver and occupy a strategic position at the crossroads of metabolic flux and immune surveillance (4). During MASLD, chronic nutrient excess exposes LSECs to lipotoxic intermediates, oxidative stress, and damage-associated molecular patterns (5). These insults activate innate immune sensors highly expressed in LSECs, altering the adhesion and function of immune cells within the hepatic sinusoidal microenvironment (6). However, how LSEC-intrinsic innate immune pathways convert metabolic stress into inflammatory regulatory signals remains poorly understood.

Among innate immune pathways, the cyclic GMP-AMP synthase/stimulator of interferon genes (cGAS/STING) axis has emerged as a central integrator of cytosolic DNA sensing (7, 8). Beyond its established role in host defense, accumulating evidence suggests that STING can be activated by metabolic perturbations, including mitochondrial DNA release and oxidative injury — hallmarks of steatohepatitis (9, 10). While STING activation in macrophages amplifies inflammatory responses, its endothelial role and potential contribution to sinusoidal immunometabolic remodeling remain undefined (11, 12). Given the quantitative predominance and unique spatial position of LSECs at the vascular–metabolic interface, determining whether LSEC-intrinsic cGAS/STING signaling integrates metabolic stress signals to reshape the hepatic niche is essential.

Here, we identify endothelial STING as a vascular metabolic checkpoint that couples cytosolic stress sensing to angiocrine regulation of adaptive immunity. Through human single-nucleus transcriptomics, endothelium-specific genetic models, and targeted nanoparticle delivery, we demonstrate that STING-dependent repression of BMP4 disrupts sinusoidal immunometabolic homeostasis and promotes disease progression in MASLD.

## Results

### Metabolic stress selectively activates endothelial cGAS/STING signaling during MASLD progression.

To define how LSECs respond to chronic metabolic overload in human disease, we analyzed a published single-nucleus RNA-seq (snRNA-seq) dataset comprising 47 human liver biopsies spanning multiple disease stages (Figure 1, A and B) (13). Reclustering of LSECs revealed pronounced transcriptional heterogeneity across MASLD stages, with distinct gene expression programs and pathway enrichment profiles (Figure 1, C–E). Gene set variation analysis revealed a progressive suppression of endothelial homeostasis and endocytic pathways (Figure 1E), indicating functional deterioration of LSECs during MASLD progression. Concomitantly, lipid metabolism–associated pathways were increasingly enriched, consistent with sustained metabolic overload within the sinusoidal niche (Figure 1E). These coordinated transcriptional changes indicate that LSECs undergo metabolic and functional rewiring during MASLD progression.

We next investigated whether metabolic stress reshapes innate immune programs in LSECs. Lipotoxicity-induced cellular injury can activate innate immune responses in MASLD (2). Given that LSECs and macrophages are the 2 predominant cell populations governing hepatic innate immunity, we examine the interrelationships among lipid metabolism, cellular damage, and innate immune activation–related pathways in them. Our results revealed that these 3 elements were more tightly correlated in LSECs compared with macrophages (Figure 1F). Further analysis indicated that the correlation between lipid metabolism and innate immune activation was also stronger in LSECs (Figure 1, G and H), with the cGAS/STING/cytosolic DNA-sensing pathway — rather than TLR, RIG-I–like receptor (RLR), or NLR pathways — showing the closest association with lipid metabolic perturbations (Figure 1H). Notably, only the cGAS/STING axis exhibited robust and stage-dependent activation in LSECs during MASLD progression, paralleling the emergence of metabolic stress signatures (Figure 1E). This activation initiates key downstream signaling pathways, most notably the interferon-responsive transcriptional programs (14); concurrently, pathways associated with leukocyte adhesion and tissue injury were upregulated (Figure 1E). These findings indicate that the LSEC-intrinsic cGAS/STING pathway links metabolic stress to inflammatory niche remodeling.

To independently validate these findings, we applied the AddModuleScore approach to quantify pathway activity in LSECs. Both the cGAS/STING and RLR signaling pathways were activated, with cGAS/STING signaling exhibiting the most pronounced increase (Figure 1I and Supplemental Figure 1A; supplemental material available online with this article; https://doi.org/10.1172/JCI207031DS1). Consistent with prior reports implicating cGAS/STING signaling in hepatic macrophages (11, 12), we observed robust pathway activation within macrophage populations (Figure 1J). Strikingly, LSECs demonstrated a comparable degree of activation, identifying endothelial cells as an underappreciated innate immune responder in MASLD.

We next experimentally validated these findings. Immunofluorescence analysis revealed increased dsDNA accumulation in hepatic sinusoids of human metabolic dysfunction–associated steatohepatisis (MASH) livers relative to healthy controls (Supplemental Figure 1B). To investigate whether LSECs constitute a source of this dsDNA, we treated primary LSECs with palmitic acid (PA), a predominant free fatty acid in MASH. PA directly triggered mtDNA release — a well-established source of dsDNA in MASH (10) — and promoted phosphorylation of p65 and IRF3, 2 established markers of STING pathway activation (Supplemental Figure 1, C and D) (14). Notably, depleting mtDNA with 2′,3′-dideoxycytidine (15) abolished STING pathway activation under PA treatment (Supplemental Figure 1, C and D). These findings establish a lipotoxicity/mtDNA release/STING activation axis in LSECs. We position cGAS/STING as a dominant endothelial stress-sensing axis selectively engaged during metabolic overload, linking chronic nutrient excess to transcriptional reprogramming within the sinusoidal niche.

### STING is endothelium enriched and progressively amplified under metabolic stress.

In the snRNA-seq dataset, we noticed that STING itself emerged as the predominantly expressed molecule among the core signaling factors (cGAS, STING, TBK1, and IRF3) within LSECs (Supplemental Figure 1E). To further confirm this, we interrogated the Human Protein Atlas. Similarly, data showed that STING transcripts are preferentially expressed in human LSECs relative to other core components of the cGAS/STING pathway, and STING expression levels in LSECs were comparable with those observed in Kupffer cells (KCs), in which STING exerts a pro-MASH role (Figure 2A) (11, 12). We then recapitulated this finding in primary murine hepatic cell populations (Figure 2B). At the protein level, costaining with endothelial markers confirmed robust STING expression — rather than cGAS, TBK1, or IRF3 — within sinusoidal endothelial cells across species (Figure 2C and Supplemental Figure 2, A and B). Together, these findings indicate that endothelial cells constitute a quantitatively significant reservoir of STING signaling capacity.

We next investigated whether endothelial STING expression is further amplified during MASLD. Western blot analysis of primary LSECs isolated from 2 distinct murine MASH models — the choline-deficient, l-amino acid–defined, high-fat diet (CDAHFD) and the Western diet combined with CCl_4_ administration (WD-CCl_4_) models — revealed increased STING protein abundance accompanied by enhanced phosphorylation of p65 and IRF3 during disease progression (Figure 2, D and E, and Supplemental Figure 2C). Consistently, GSEA of a murine MASH-associated LSEC transcriptome (16) demonstrated significant activation of the cGAS/STING pathway and downstream signaling modules, including type I interferon production and NF-κB signaling (Figure 2F) (14). Furthermore, interrogation of another human single-cell atlas (17) demonstrated preferential enrichment of STING, RELA (p65), and IRF3 transcripts during endothelial-to-mesenchymal transition (Supplemental Figure 2D), a phenotypic shift linked to sinusoidal capillarization and fibrosis in MASH (5, 18). Together, these data indicate that STING is not only intrinsically enriched within hepatic endothelium, but is progressively amplified under metabolic stress.

### Endothelial STING is required for progression from metabolic stress to inflammatory-fibrotic niche collapse.

To investigate the role of cGAS/STING signaling in LSECs during MASH pathogenesis, we generated endothelial cell–specific *Sting*-knockout mice (*Sting*^fl/fl^ *Cdh5*-Cre^+^ *Sting*^ΔEC^) and littermate controls (*Sting*^fl/fl^ *Cdh5*-Cre^–^ *Sting*^fl/fl^) (Supplemental Figure 3, A–D), prompted by the abundant expression of STING in LSEC. *Sting*^ΔEC^ did not affect sinusoidal endothelial fenestration under basal conditions (Supplemental Figure 3E). Moreover, *Sting*^ΔEC^ mice maintained on a normal chow diet displayed no significant differences in body weight, liver weight, blood glucose levels, or hepatic histological architecture compared with control littermates (Supplemental Figure 4, A–F), indicating that LSEC-STING is dispensable for liver homeostasis under physiological conditions.

We next subjected *Sting*^ΔEC^ mice and the littermate controls to the 2 independent murine MASH models (Figure 3, A and B). *Sting*^ΔEC^ successfully inhibited the activation of its 2 downstream effectors in the MASH model (Supplemental Figure 5A). Across both models, livers from *Sting*^ΔEC^ mice exhibited a significantly reduced NAFLD activity score (NAS), a composite index encompassing steatosis, lobular inflammation, and hepatocellular ballooning, compared with controls (Figure 3, C and D). Among these components, lobular inflammation was consistently and markedly attenuated in livers from *Sting*^ΔEC^ mice in both models (Figure 3, C and D), whereas steatosis was markedly attenuated only in the WD-CCl_4_ model, likely due to the potent and pathology-misaligned steatogenic effect of the CDAHFD model (Figure 3, E and F, Supplemental Figure 5, D and H) (19). In contrast, hepatic fibrosis was robustly alleviated in *Sting*^ΔEC^ mice across models (Figure 3, G and H, and Supplemental Figure 5, E and I), accompanied by a concomitant reduction in the SAF score, which integrates steatosis, inflammatory activity, and fibrosis (Figure 3, I and K). Consistent with these histological improvements, LSEC-STING deficiency significantly mitigated liver injury during MASH, as reflected by reduced serum levels of alanine aminotransferase (ALT), aspartate aminotransferase (AST), and total bilirubin (TBIL) (Figure 3, J and L). However, LSEC-STING deletion did not alter body weight, liver weight, or blood glucose levels during MASH progression (Supplemental Figure 5, B, C, F, and G), indicating that its protective effects are independent of systemic metabolic alterations.

To gain mechanistic insight into the role of LSEC-STING in MASH, we performed bulk RNA-seq on liver tissues from *Sting*^ΔEC^ and control mice fed a CDAHFD (Supplemental Figure 5J). GSEA of KEGG and Reactome pathways revealed that multiple cancer-associated and fibrogenic signaling pathways were enriched in control livers but markedly suppressed in *Sting*^ΔEC^ livers (Supplemental Figure 5K). Conversely, pathways related to T cell regulation and metabolism were preferentially enriched in *Sting*^ΔEC^ livers (Supplemental Figure 5L). Collectively, these findings indicate that endothelium-intrinsic STING signaling drives MASH progression, likely by reshaping T cell–mediated immune responses.

### Endothelium-specific Sting knockout elevates Treg/Th17 ratio during MASH.

To determine how LSEC-intrinsic STING signaling shapes T cell responses during MASH, we performed scRNA-seq on liver tissues from *Sting*^ΔEC^ and control mice subjected to WD-CCl_4_ administration. Unbiased cell-type annotation revealed broad immune remodeling following LSEC-STING deletion (Supplemental Figure 6, A–C). Among immune populations, B cells displayed the largest relative increase in abundance; however, subclustering analysis demonstrated a selective reduction in plasmocytes in *Sting*^ΔEC^ livers, suggesting attenuation of plasmocyte-associated inflammatory responses (Supplemental Figure 6, D and E). In parallel, activation markers in mesothelial cells and hepatic stellate cells were reduced in livers from *Sting*^ΔEC^ mice (Supplemental Figure 6F), consistent with the diminished fibrotic phenotype observed in these animals.

Given the enrichment of T cell–related pathways in *Sting*^ΔEC^ livers, we next focused on CD4^+^ T cells, which were resolved into 4 distinct subsets (Figure 4A and Supplemental Figure 7A). LSEC-STING deletion resulted in a marked reduction of Th17 and Th1, accompanied by a concomitant expansion of Treg, leading to a significantly increased Treg/Th17 ratio in *Sting*^ΔEC^ livers compared with controls (Figure 4A). As Th17 can be stratified into pathological and nonpathological subsets based on established molecular signatures (20), and pathological Th17 has been implicated as a key driver of MASH pathogenesis (3, 21–24), we further examined Th17 heterogeneity. The data indicate that the majority of Th17 in the MASH liver was pathological, and LSEC-STING deficiency markedly reduced the pathological Th17 population, defined as *Il23r*^+^ Th17, based on the decisive role of IL-23 in conferring pathogenicity (Figure 4B) (25).

Further subclustering of Treg identified 3 distinct subsets, among which LSEC-STING deletion predominantly expanded the Treg_1 population (Figure 4C and Supplemental Figure 7B). Treg_1 constituted the major fraction of the Treg compartment and was characterized by selective regulation of IL-17/Th17–associated responses (Figure 4D and Supplemental Figure 7, C and D). Consistent with this, Treg_1 displayed increased activity of transcription factors (TFs) associated with immune tolerance (e.g., *Tcf4*, *Nr4a2*, *Pbx1*), while proinflammatory TFs (e.g., *Stat1*, *Mxi1*, *Zeb1*) were suppressed (Figure 4E). In contrast, proinflammatory signaling pathways were preferentially enriched in the Treg_2 and Treg_3 subsets rather than in Treg_1 (Figure 4F). Collectively, these data indicate that LSEC-STING deletion skews the intrahepatic CD4^+^ T cells toward an antiinflammatory state, characterized by reduced pathological Th17 and expanded functionally suppressive Treg during MASH.

To validate these scRNA-seq findings, we performed flow cytometry analysis of immune populations. Consistent with the transcriptomic data, the frequency of Th17 was significantly decreased, whereas Tregs were increased, in the livers of *Sting*^ΔEC^ mice (Figure 4G). Notably, no corresponding changes were observed in peripheral blood or spleen (Supplemental Figure 7, E and F), indicating that LSEC-STING selectively regulates CD4^+^ T cell composition within the hepatic niche without altering systemic immunity.

### LSEC-STING regulates Th17/Treg fate decision through BMP signaling–mediated intercellular communication.

We next sought to elucidate the mechanisms by which LSEC-intrinsic STING signaling regulates the Treg/Th17 balance during MASH. Given the pronounced plasticity of CD4^+^ T subsets and their capacity for interconversion (26, 27), we first examined CD4^+^ T cell differentiation trajectories. CytoTRACE analysis identified tissue-resident memory T cells (Trm) as the least differentiated population, whereas Tregs occupied a terminally differentiated state (Figure 4H), a hierarchy that was further supported by pseudotime analysis (Figure 4I). These observations suggest that LSEC-intrinsic STING signaling may govern hepatic CD4^+^ T cell fate decision in MASH.

We therefore analyzed intercellular communication between LSECs and CD4^+^ T subsets. Cell interaction analysis revealed that, in *Sting*^ΔEC^ livers, LSECs exhibited enhanced crosstalk with Trm and Th17 through the bone morphogenetic protein (BMP) signaling axis (Figure 4J and Supplemental Figure 7G), a pathway that elevates Treg/Th17 ratio in CD4^+^ T cells (28, 29). In contrast, STING-deficient LSECs displayed minimal direct interaction with Treg and lacked enrichment of chemotactic pathways (Supplemental Figure 7G), suggesting that LSEC-STING regulates intrahepatic Treg abundance primarily by modulating transdifferentiation of CD4^+^ T cells rather than by direct recruitment of Tregs. Consistent with these findings, scRNA-seq analysis demonstrated that BMP ligands were predominantly expressed by LSECs, and *Sting*^ΔEC^ deletion led to a marked upregulation of BMP ligands compared with controls (Figure 4K). Together, these data support a model in which STING restrains endothelial BMP production under metabolic stress, thereby limiting BMP-dependent Treg polarization and favoring pathological Th17 differentiation.

### STING/NF-κB signaling inhibits Treg skewing by suppressing BMP4 transcription.

To validate that LSEC-STING regulates CD4^+^ T cell differentiation via BMP signaling, we focused on BMP4, the most prominently activated BMP ligand in LSEC–CD4^+^ T cell crosstalk (Figure 4J). ELISA results demonstrated that BMP4 was upregulated in *Sting*^ΔEC^ livers compared with controls (Figure 5A). Furthermore, BMP4 in combination with TGF-β and IL-2 promoted naive CD4^+^ T cell differentiation into Tregs more efficiently than the TGF-β and IL-2 treatment alone (Figure 5B).

To delineate the regulatory mechanism between STING and BMP, we next examined TFs altered in STING-deficient LSECs (Supplemental Figure 8A). Based on prior studies and predicted binding affinity to *Bmp4*, we shortlisted 4 candidate TFs: EBF1, THRB, PBX1, and HEY1 (Supplemental Figure 8B). However, none of these TFs induced *BMP4* expression in vitro (Supplemental Figure 8, C and D), prompting us to explore alternative regulatory pathways. Utilizing the NetworkAnalyst platform (30), we identified NF-κB, a canonical downstream effector of the cGAS/STING pathway, as a predicted regulator of *BMP4* transcription (Figure 5C). To validate this prediction, we treated mouse primary LSECs with TNF-α to activate NF-κB signaling, which led to a significant suppression of *BMP4* expression (Figure 5D). Luciferase reporter assays further showed that p65, a subunit of NF-κB, significantly inhibited *BMP4* promoter activity on its own (Figure 5E). Motif analysis identified several putative p65-binding sites within the *BMP4* promoter (Figure 5F and Supplemental Figure 8E), and ChIP assays confirmed direct binding of p65 to the *BMP4* promoter region (Figure 5, G and H, and Supplemental Figure 8, F and G).

To obtain direct evidence, we isolated primary LSECs from our *Sting*^ΔEC^ and control mice and found that, under PA-induced lipotoxic conditions, *Bmp4* expression was elevated in *Sting*-deficient LSECs; this increase could be suppressed by TNF-α (Figure 5, I and J). Correspondingly, naive CD4^+^ T cells treated with conditioned medium of *Sting*-deficient LSECs were more likely to differentiate into Tregs, and this effect was blocked by TNF-α (Figure 5, K and L).

### NF-κB suppresses BMP4 transcription by disrupting AP-1 binding to its promoter.

Given that NF-κB is more commonly regarded as a transcriptional activator, we hypothesized that NF-κB binding to the BMP4 promoter might interfere with the binding of its positive TFs. We therefore analyzed our previously identified ChIP-qPCR–positive region (–1,146 to –1,004) and compared it with the reported motifs of BMP4-activating TFs, including Activator Protein-1 (AP-1) (31, 32), SP1 (33), and SMAD-1/5/8 (34). Notably, we identified an AP-1–binding site (–1,107 to –1,100) adjacent to the NF-κB–binding site (–1,087 to –1,077) (Figure 5M).

Subsequent experiments confirmed that NF-κB can compete with AP-1 for binding to the *BMP4* promoter (Figure 5N), thereby affecting promoter activity (Figure 5O) and *Bmp4* expression (Figure 5P). We further found that the elevated *Bmp4* expression in *Sting*-deficient LSECs under PA-induced lipotoxic conditions could be suppressed by T-5224 (Figure 5Q), a specific inhibitor of AP-1 binding to DNA (35, 36), further supporting the AP-1 dependence of this effect.

### Inhibition of BMP signaling abrogates the protective effects conferred by endothelium-specific Sting knockout.

To validate the functional relevance of the BMP signaling pathway in vivo, we performed rescue experiments in the WD-CCl_4_ MASH model (Figure 6A). Pharmacological inhibition of BMP signaling by LDN-193189 (37) largely abrogated the protective effects conferred by *Sting*^ΔEC^, reversing improvements in NAS (Figure 6B), lipid accumulation (Figure 6C), fibrosis (Figure 6D), SAF score (Figure 6E), serum markers of liver injury (Figure 6, F–H), and Treg and Th17 proportions (Figure 6, I and J). Combined with the finding that BMP ligands were predominantly expressed by LSECs (Figure 4K), these data demonstrate that endothelium-derived BMP signaling is required for the immunomodulatory and hepatoprotective effects observed in *Sting*^ΔEC^ mice, functionally positioning BMPs as critical angiocrine mediators downstream of STING.

### Upregulated BMP4 in human MASH liver represents an angiocrine stress–adaptive axis and a potential therapeutic target.

Having established that *Sting*^ΔEC^ alleviates MASH by relieving NF-κB–mediated repression of BMP4 in LSECs, we next asked whether BMP4 might represent a therapeutically actionable target in human MASLD. To this end, we interrogated hepatic transcriptomes from patients with MASLD. Analysis of a public dataset (38) revealed that hepatic *BMP4* mRNA levels were significantly elevated in patients with MASH and stage 4 fibrosis; in contrast, *BMP4* expression was not increased when the same cohort was stratified by NAS (Supplemental Figure 9A), indicating that *BMP4* upregulation is more closely associated with fibrosis severity rather than steatosis or inflammatory activity. Consistently, hepatic *BMP4* mRNA levels exhibited strong positive correlations with established fibrosis marker genes, including *COL1A1* and *ACTA2* (Supplemental Figure 9B). These observations were independently validated in an additional human MASLD dataset (39) with a smaller sample size (Supplemental Figure 9, C–E). However, serum proteomic analysis of a human MASLD cohort (40) revealed only a slight increase in BMP4 levels in patients with MASLD (Supplemental Figure 9F), suggesting that BMP4 upregulation predominantly occurs within the hepatic microenvironment and likely reflects a localized angiocrine response rather than a systemic endocrine signal. Together, these findings imply that increased hepatic BMP4 expression in advanced MASH likely reflects a local compensatory response to progressive fibrogenesis, which is nonetheless insufficient to effectively restrain fibrosis progression.

We therefore reasoned that pharmacological amplification of this endogenous angiocrine tolerance program might confer therapeutic benefit. To test this hypothesis, we treated MASH mice with 2 BMP agonists: SB4, a specific BMP4 signaling agonist (41), and SJ000291942, a pan-BMP signaling agonist (42) (Supplemental Figure 10A). While the BMP4 agonist SB4 activated the canonical BMP effector Smad1/5/9 to a lesser extent than the pan-BMP agonist SJ000291942 (Supplemental Figure 10B), the 2 compounds showed nearly equivalent efficacy in reversing MASH progression. Treatment with either agonist led to significant reduction in disease phenotypes, including NAS, lipid accumulation, fibrotic area, SAF score, hepatic Th17/Treg ratio, and liver injury biomarkers, in the WD-CCl_4_ model (Supplemental Figure 10, C–K). Collectively, these data suggest that BMP4 upregulation in advanced human MASH reflects an endogenous but inadequate angiocrine stress–adaptive response. Therapeutic reinforcement of BMP4 signaling may therefore restore endothelial-immune crosstalk and represent a viable strategy for the treatment of progressive MASH.

### Human single-cell analysis validates a conserved endothelial STING-BMP4 angiocrine checkpoint.

To validate the LSEC-intrinsic STING/BMP4 signaling axis in human MASH, we analyzed another scRNA-seq dataset of human liver cirrhosis (43). Consistently, among all major hepatic cell types, *STING* expression was uniquely enriched in liver endothelial cells, whereas other parenchymal and immune populations exhibited markedly lower expression levels (Figure 7, A–C, and Supplemental Figure 11, A and B). To quantitatively assess innate immune pathway activity across cell types, we applied 4 independent scoring approaches. Across all scoring approaches, cGAS/STING signaling activity was highest in endothelial cells, despite the observation that disease-associated dynamic changes in pathway activation were more pronounced in macrophages (Figure 7, D and E).

Stratification of liver endothelial cells based on *STING* expression revealed a striking functional dichotomy. *STING*^–^ LSECs were enriched for BMP signaling pathways, as well as the broader TGF-β signaling network to which BMP signaling belongs (Figure 7, F and G) (44). Among all identified BMP ligands expressed in the mouse scRNA-seq data, *BMP4*, but not *BMP2* or *BMP6*, was selectively and robustly expressed in endothelial cells (Figure 7, H and I, and Supplemental Figure 11, C and D). Importantly, *BMP4* expression exhibited a strong inverse correlation with *STING* levels at the single-cell level (Figure 7J), consistent with STING-mediated repression of BMP4 observed in murine LSECs. Finally, ligand–receptor interaction analysis revealed that *STING*^–^ LSECs engaged more extensively with CD4^+^ T cells through BMP ligand-receptor pairs identified in mouse scRNA-seq, compared with their *STING*^+^ counterparts (Figure 7, K–M), indicating that the angiocrine communication axis linking endothelial BMP4 to CD4^+^ T cell fate is conserved in human disease. Collectively, these human single-cell analyses validate a conserved endothelial STING/BMP4 angiocrine checkpoint that governs endothelial CD4^+^ T cell crosstalk in MASLD, extending our mechanistic findings from mouse models to human liver pathology.

### Endothelium-specific Sting deletion restrains CD8^+^ T cell exhaustion during MASH.

In addition to CD4^+^ T cells, we found that LSEC-intrinsic STING signaling also influences CD8^+^ T cell states during MASH progression. Single-cell analysis revealed that *Sting*^ΔEC^ markedly reduced the frequency of exhausted CD8^+^ T cells (Tex), concomitant with an increase in effector CD8^+^ T cells (Teff) and Trm cells in the liver (Supplemental Figure 12A). Functional enrichment analysis demonstrated that Teff cells were preferentially enriched for pathways associated with IFN-γ production and cytotoxic effector functions (Supplemental Figure 12B), whereas Tex cells exhibited strong enrichment of inhibitory checkpoint signaling, including the PD-1/PD-L1 pathway (Supplemental Figure 12C). Consistently, TFs associated with T cell exhaustion (e.g., *Zfp64, Eomes, Batf*) were selectively activated in Tex cells, while transcriptional regulators promoting effector differentiation and function (e.g., *Rora, Runx1, Nfkb1*) were enriched in Teff cells (Supplemental Figure 12D). To delineate the developmental origin of Tex cells, we performed CytoTRACE and pseudotime analyses, which revealed that Tex cells primarily arise from hepatic Trm and Teff populations (Supplemental Figure 12, E and F). These findings were independently validated by flow cytometry, which confirmed a significant reduction in Tex frequency in the livers of *Sting*^ΔEC^ mice, with no corresponding changes in the circulation or spleen (Supplemental Figure 12, G–I). This liver-restricted modulation of CD8^+^ T cell exhaustion parallels our findings in CD4^+^ T cell fate reprogramming, reinforcing the concept that endothelial STING acts as a local angiocrine checkpoint governing adaptive immune fitness within the hepatic niche.

### Delivery of a STING inhibitor via LSEC-directed RLTR-liposome ameliorates MASH progression.

To target aberrant activation of the cGAS/STING pathway in LSECs during MASH, we engineered RLTR-liposome@H-151 (RLH) (Figure 8A). A stabilin-2–targeted peptide, RLTRKRGLK (RLTR), which confers high specificity toward LSEC (45), was conjugated to DMPE-PEG2000-NHS to generate DMPE-PEG2000-RLTR (Supplemental Figure 13, A and B). This conjugate was subsequently incorporated onto the surface of liposomes, yielding RLTR-functionalized liposomes. The potent covalent STING inhibitor H-151 (46) was then encapsulated within these nanoparticles to generate RLH (Supplemental Figure 13C). Both RLH and empty RLTR-liposome exhibited a uniform size distribution with an average diameter of approximately 110  nm (Supplemental Figure 13, D and E), and H-151 was efficiently encapsulated, achieving an encapsulation efficiency of 84.3% (Supplemental Figure 13, F and G).

We next evaluated the therapeutic efficacy of RLH in the WD-CCl_4_–induced MASH model. Intravenous administration of RLH, containing H-151 at a dose of 1 mg/kg, was performed 8 times during weeks 5–8 after disease induction. For comparison, a separate group received systemic H-151 via intraperitoneal injection at 10  mg/kg following the same treatment schedule (Figure 8B). In vivo imaging demonstrated hepatic enrichment of RLH (Figure 8C) and its subsequent uptake by LSECs (Figure 8D). Notably, RLH achieved substantial inhibition of p65 and IRF3 phosphorylation in LSECs but had minimal effect in KCs (Figure 8E). Despite a 10-fold reduction in administered dose, RLH achieved therapeutic efficacy comparable with that of systemic H-151, as evidenced by similar improvements in hepatic lipid accumulation, fibrosis, and inflammatory responses (Figure 8, F–N), effectively phenocopying the protective effects observed in endothelium-specific *Sting* deletion. These findings demonstrate that inhibition of endothelial STING within the hepatic angiocrine compartment is sufficient to attenuate MASH progression, highlighting endothelial STING as a druggable vascular immune checkpoint.

## Discussion

LSECs occupy a central anatomical and functional position at the interface between metabolic flux and immune surveillance, yet how they integrate nutrient stress to shape adaptive immunity has remained unclear. Here, we identify endothelial STING as a vascular immunometabolic switch that couples chronic nutrient overload to angiocrine remodeling, adaptive immune polarization, and fibrotic progression. Rather than acting merely as a proinflammatory sensor, LSEC-intrinsic STING signaling reprograms the endothelial secretome, undermining a tolerance-supporting niche and driving coordinated alterations in CD4^+^ and CD8^+^ T cell states. These findings position the hepatic endothelium as a metabolically sensitive immune checkpoint within the MASH microenvironment.

STING has long been regarded as an innate immune sensor mainly expressed in immune cells (47). However, recent studies have shown that STING is also present in endothelial cells (15, 48–51). In the liver, its expression in LSECs has remained unclear. While one study reported a role for LSEC-STING in shaping hepatocellular carcinoma vasculature (52) and another described increased endothelial STING expression in MASH (12), a separate report concluded that STING is largely absent from the liver (53). These conflicting observations likely reflect differences in detection methods and cell-type resolution.

To address this, we profiled STING expression across major hepatic cell populations by analyzing snRNA-seq and scRNA-seq data of human livers, with subsequent experimental validation. We reveal that STING is readily detectable in LSECs under physiological conditions at levels comparable with those in macrophages and that both LSEC-STING expression and signaling activity are further elevated during MASLD progression. A recent study has shown that STING protein can be upregulated by gut microbiota (54). Given that gut microbial translocation occurs in MASH (55), it is reasonable that STING upregulation in LSECs results from stimulation by translocated microbial products within the hepatic microenvironment. On this basis, mtDNA released by fatty acid–induced cellular stress can more readily activate the cGAS/STING signaling pathway. Since LSECs are far more abundant than macrophages in the liver, our findings suggest that targeting STING signaling in this numerically dominant compartment may represent a particularly impactful therapeutic strategy.

Prior to our investigation, the role of LSEC-intrinsic STING signaling in MASLD was poorly understood (9). Here, we uncover its function in shaping the hepatic Th17/Treg balance during MASH progression. Th17 cells are recently considered major contributors to the pathogenesis of MASLD (3, 21–24), as an increased Th17/Treg ratio marks the transition from simple steatosis to MASH (56). Importantly, disease severity and clinical outcomes in MASH are determined primarily by the extent of fibrosis rather than lipid accumulation (57–59), placing immune programs linked to fibrogenic progression, such as Th17 responses, at the center of pathogenic relevance. Within this metabolic context, endothelial STING functions as a vascular switch that translates metabolic stress signals into adaptive immune polarization, thereby coupling immunometabolic imbalance to fibrotic remodeling.

Mechanistically, we uncover a direct link between LSEC-STING and BMP signaling–mediated regulation of CD4^+^ T cell fate. As a key member of the TGF-β superfamily, previous research on BMP4 has primarily focused on its roles in fibrosis, angiogenesis, and tissue homeostasis (44). In recent years, the function of BMP signaling in T cell differentiation has gradually gained attention, with existing studies indicating that BMPR1, one of the receptors for BMP proteins, plays a crucial role in Treg and Th17 differentiation (28, 29). However, within the pathological context of MASH, the regulatory mechanisms of BMP4 expression and its role in the hepatic immune microenvironment have not yet been thoroughly investigated. Our findings extend this concept by demonstrating that BMP4 functions as an endothelium-derived angiocrine cue that reinforces Treg while inhibiting Th17 lineage commitment within the hepatic microenvironment. Under metabolic stress conditions characteristic of MASLD, sustained STING activation represses BMP4 transcription, effectively disrupting a tolerance-supporting angiocrine program. Notably, BMP4 expression was largely restricted to LSECs rather than macrophages, supporting the idea that modulation of Th17/Treg responses represents a distinct immunoregulatory function of LSEC-STING. This finding suggests that in endothelial cells, the STING/NF-κB axis does not simply exert a proinflammatory effect, but rather indirectly disrupts local hepatic immune homeostasis by suppressing the expression of immunoregulatory molecules.

Analysis of human MASH samples further supports a liver-confined role for BMP4, as its expression was high in hepatic tissue but minimal in circulation. This spatial restriction mirrors our functional data, in which LSEC-specific STING deletion altered the Th17/Treg ratio in the liver without affecting peripheral lymphoid compartments, indicating metabolic stress–induced endothelial remodeling operates primarily within the hepatic niche rather than systemically. Despite persistent STING activation in advanced disease, BMP4 expression was not uniformly suppressed, suggesting that additional regulatory inputs shape BMP signaling in vivo, among which the JNK/AP-1 (comprising c-Fos and c-Jun) axis may represent a key mechanism: JNK is reported to be activated in MASH liver (60), and AP-1 has been shown to activate *BMP4* transcription in previous studies (31, 32) and herein. However, increased BMP4 expression is insufficient to inhibit MASH progression in the absence of intervention. In this context, therapeutic activation of BMP signaling emerges as a rational strategy. Indeed, by administering SB4, a specific BMP4 signaling agonist (41), we demonstrate that enhancement of BMP signaling effectively improves fibrotic pathology, supporting BMP4 agonism as a translationally promising approach for MASLD treatment. Given the complex expression patterns and pleiotropic functions of BMP4 in MASLD, additional translational studies are needed prior to clinical translation.

In addition to the regulation of the LSEC/STING/BMP4 axis in CD4^+^ T cells, our findings further reveal a pivotal role of LSEC-intrinsic STING in driving CD8^+^ T cell exhaustion. While acute STING activation can support effective antitumor immunity, increasing evidence indicates that chronic STING signaling instead promotes a protumorigenic immune state (61). This distinction is particularly relevant in MASH-associated hepatocellular carcinoma (HCC), which not only shows poor responsiveness to immunotherapy but has also been reported to exhibit paradoxical tumor acceleration following immune checkpoint blockade, in contrast to virus-induced HCC (62, 63).

Consistent with this notion, we reveal that endothelium-specific STING deficiency markedly reduced the accumulation of exhausted CD8^+^ T cells specifically within the liver, but not in peripheral blood or spleen. Such chronic activation likely reflects persistent metabolic stress signaling within the hepatic vasculature, further linking nutrient overload to adaptive immune dysfunction. Moreover, while transcriptomic analyses of the normal control group revealed pronounced activation of cancer-related pathways, these signatures were largely absent in the STING-deficient group. Together, these findings indicate that although inhibition of LSEC-STING in MASH increases Treg abundance, cells that have been implicated in tumor promotion, it nevertheless confers a net reduction in tumor-promoting immune pressure by attenuating Th17-driven protumorigenic inflammation (64) and limiting CD8^+^ T cell exhaustion.

LSECs are particularly well suited for nanoparticle-based targeting owing to their highly permeable fenestrae and exceptional endocytic capacity, supported by abundant scavenger receptors, most notably stabilin-1/2, which are major and relatively specific scavenger receptors on LSECs. This same property underlies the well-recognized hepatic accumulation and toxicity observed with many systemically administered nanomedicines (65–67). Leveraging this biological feature, we developed RLH by controlling nanoparticle size to approximately 100  nm, which favors uptake by LSECs instead of KCs (68), and by incorporating a stabilin-2–targeting peptide to further enhance endothelial delivery. This design markedly reduced the effective dose required to achieve therapeutic benefit, reaching efficacy comparable with intraperitoneal administration of a 10-fold higher dose of free H-151. By spatially confining inhibition to a metabolically responsive endothelial compartment, this strategy limits systemic interference with innate immune surveillance while restoring angiocrine immune-metabolic balance.

This study has several limitations. First, consistent with most studies investigating hepatic endothelium (16), we employed systemic endothelial STING deletion rather than LSEC-restricted genetic ablation. However, MASH-associated pathological changes and immune remodeling were largely confined to the liver, supporting a dominant role for hepatic endothelial STING in this context. Second, single-cell transcriptomic analyses suggest that LSEC-STING may also influence additional nonparenchymal populations, including B cells and hepatic stellate cells, which warrants dedicated investigation in future studies. Third, the use of mice with endothelium-specific deletion of *Bmp4* in rescue experiments would provide more compelling evidence. Finally, owing to the limited capacity of current murine models to fully recapitulate the immunopathology of human MASH-HCC, the contribution of LSEC-STING–mediated CD8^+^ T cell exhaustion to MASH-HCC progression remains to be established.

Collectively, this study positions endothelial STING as a central immunometabolic regulator within the hepatic vascular niche and underscores the therapeutic potential of targeting angiocrine checkpoints in progressive MASLD.

## Methods

Additional details may be found in Supplemental Methods.

### Sex as a biological variable.

Our study examined male mice because male animals exhibited less variability in phenotype. It is unknown whether the findings are relevant for female mice.

### Human liver samples.

Human liver specimens were collected from patients receiving hepatic resection within the Department of Hepatobiliary Surgery at Xijing Hospital, Fourth Military Medical University. Participants selected for the study had no history of drug- or toxin-related liver injury, hepatitis viral infections, or significant alcohol intake. Evaluation of NAS and SAF scores was performed independently by a minimum of 2 pathologists. They assessed H&E along with Masson’s trichrome–stained tissue sections in a blinded manner, following the criteria established by the NASH-CRN (69) and SAF (70) scoring systems, respectively. Individuals with a NAS exceeding 4 were assigned to the NASH cohort. The protocol for human sample acquisition and usage received ethical approval from the Institutional Review Board of Xijing Hospital and was conducted in accordance with the Declaration of Helsinki. Written informed consent was provided by all subjects.

### Generation and genotyping of Sting^ΔEC^ mice.

The *Sting*^fl/fl^ (*Tmem173*-Flox) mice were purchased from Shanghai Model Organisms Center (NM-CKO-00014). In this model, a loxP site was inserted between the 5′ homology arm of exon 3 and the 3′ homology arm of exon 5. The *Sting*^fl/fl^ mice were then crossed with *Cadherin-5*-Cre (*Cdh5*-Cre) transgenic mice (006137, The Jackson Laboratory) to delete exons 3–5, thereby generating endothelial cell–specific *Sting* (*Tmem173*)–knockout (*Sting*^ΔEC^) mice. Genotype verification was performed using PCR followed by gel electrophoresis. For detection of the flox allele, primers P1–P3 were used, which produced an 800 bp band in floxed mice and a 672 bp band in the controls. For Cre identification, primers P4–P7 were used, yielding a 345 bp band in Cre^+^ mice and a 521 bp band in negative mice. All primer sequences (P1–P7) are provided in Supplemental Table 1.

### Animal models.

All experimental procedures involving animals were approved by the Animal Care and Use Committee of the Fourth Military Medical University. The animals were treated humanely in accordance with the guidelines provided in the Guide for the Care and Use of Laboratory Animals (National Academies Press, National Institutes of Health). Male C57BL/6J mice, 6–8 weeks old, were housed under specific pathogen–free conditions with a 12-hour light/dark cycle, a temperature of 22°C–24°C, and humidity maintained at 40%–70%. Mice were fed a normal control diet (XTI01WC, Xietong Bio-Engineering) with a caloric distribution of 21.5% protein, 11.1% fat, and 67.4% carbohydrates. To establish the CDAHFD model, mice were fed a diet (A06071309, Research Diets) with a caloric distribution of 18% protein, 46% fat, and 36% carbohydrates for 10 weeks. For the WD-CCL_4_ model, mice were fed a diet (D12079B, Research Diets) with a caloric distribution of 17% protein, 40% fat, 43% carbohydrates, and 0.21% cholesterol for 12 weeks. During the final 4 weeks, these mice were administered intraperitoneal injections of CCL_4_ (12.5%; 1 mL/kg) twice per week. The WD-CCL_4_–induced mice were administered intraperitoneal injections of BMP signaling inhibitor LDN-193189 (HY-12071, MedChemExpress; 3 mg/kg), BMP4 agonist SB4 (HY-124697, MedChemExpress; 3 mg/kg), or pan-BMP agonist SJ000291942 (HY-112331, MedChemExpress; 3 mg/kg) twice per week during the final 4 weeks, additionally. The H-151 (HY-112693, MedChemExpress; 10 mg/kg) or RLH (H-151, 1 mg/kg) was injected into the WD-CCL_4_–induced mice from week 5 to week 8. Euthanasia was performed using isoflurane (2%, 0.5 L/min) in the final animal euthanasia practice.

### Cell lines and primary cells.

HEK293T (GNHu17) cells were obtained from the Cell Bank of the Chinese Academy of Sciences. All cell lines were confirmed to be free of mycoplasma contamination prior to use. Primary hepatocytes and hepatic nonparenchymal cells were isolated from 8- to 10-week-old male C57BL/6 mice using an established protocol (71). Briefly, after anesthesia, mice were perfused via the inferior vena cava with Liver Perfusion Medium (17701-038, Thermo Fisher Scientific) followed by Liver Digestion Medium (17701-034, Thermo Fisher Scientific). The liver was then excised, minced, and filtered through a 70 μm cell strainer. Hepatocytes were pelleted by centrifugation at 50*g* for 4 min at 4°C. The resulting supernatant was further centrifuged at 350*g* for 10 min and resuspended in DMEM supplemented with 10% FBS. Subsequent separation was performed using 17.6% and 11.0% Percoll (17-0891-02, GE Healthcare) gradients followed by centrifugation at 350*g* for 10 min at 4°C. KCs and LSECs were collected from the gradient interfaces. Cells were resuspended and incubated with anti-CD146 magnetic beads (130-092-007, Miltenyi Biotec) for 30 min at 4°C in the dark. After incubation, cells were resuspended in 1 mL of washing buffer. The cell suspension was then applied to the magnetic column of a MACS separator (Miltenyi Biotec) and washed 3 times with 500 μL of washing buffer. Finally, 1 mL of washing buffer was loaded onto the MACS column, and LSECs were immediately eluted. LSECs were cultured in Endothelial Cell Medium (1001, ScienCell). Other cells were cultured in DMEM (10569010, Gibco) containing 10% FBS (F05-001-B160216, Bio-One Biotechnology) and 1% penicillin-streptomycin (15140-122, Gibco) at 37°C in a 5% CO_2_ incubator. To activate the NF-κB signaling, TNF-α (10 ng/mL) was added to the culture medium for a 12-hour stimulation. LSECs were treated with 100 μM PA (P0500, Sigma-Aldrich) for 12 hours to establish an in vitro lipotoxic model. LSECs were treated with 10 μM T-5224 (HY-12270, MedChemExpress) for 12 hours to inhibit AP-1 binding on DNA. LSECs were treated with 20 μM 2′,3′-dideoxycytidine/zalcitabine (S1719, Selleck) for 7 days to clear mtDNA.

### In vitro Treg cell differentiation.

Mice were euthanized, and spleens were harvested for single-cell preparation by mechanical dissociation and filtration through a 70 μm cell strainer. Splenocytes were resuspended in PBS containing 2% FBS, and naive CD4^+^ T cells were enriched by negative selection using an isolation kit (480039, BioLegend), according to the manufacturer’s instructions. For Treg differentiation, 24-well plates were precoated with anti-CD3 antibody (5 μg/mL; 100340, BioLegend), and naive CD4^+^ T cells were cultured in T cell expansion medium supplemented with anti-CD28 antibody (5 μg/mL; 102116, BioLegend), IL-2 (5 ng/mL; 212-12-20UG, PeproTech), and TGF-β1 (10 ng/mL; 100-21-10UG, PeproTech), with or without BMP4 (15 ng/mL; HY-P74379, MedChemExpress), at a density of 5 × 10^5^ cells per well for 5 days. To establish the in vitro coculture model, antibodies and cytokines are added to the LSEC-conditioned medium for naive CD4^+^ T cell culture. Cells were subjected to flow cytometry staining as described previously and analyzed by flow cytometry.

### Dual-luciferase reporter assay.

HEK293T cells were plated in 24-well culture plates at a density of 8 × 10^4^ cells per well. Subsequently, the cells were transfected with a luciferase reporter plasmid carrying the *BMP4* promoter or a control vector (200 ng), along with overexpression plasmids, as well as a corresponding control plasmid (200 ng). The transfection was performed using pRLTK (Renilla luciferase plasmid, 50 ng) as an internal control. After 24 hours, the cells were lysed with a commercially available reagent (E1941, Promega), and luciferase activity was measured using the Dual-Glo Luciferase Assay System (E5311, Promega) on a GloMax 20/20 Luminometer (Promega), in accordance with the manufacturer’s instructions.

### ChIP.

The putative binding sites of p65 (RELA) on the human *BMP4* promoter were predicted using JASPAR 2024 (72) and AnimalTFDB (v4.0) (73) databases. HEK293T cells transfected with overexpression plasmids were cross-linked with 1% formaldehyde for 10 min at 37°C, and the reaction was quenched with 125 mM glycine for 5 min at room temperature. Cells were washed twice with ice-cold PBS containing PMSF (ST506, Beyotime Biotechnology) and harvested using SDS Lysis Buffer from a ChIP Assay Kit (P2078, Beyotime Biotechnology). Sonication was performed under the following conditions: 260 W, 25 cycles of 30 s on/60 s off, at 4°C. The whole-cell lysate was precleared with Protein A+G agarose/salmon sperm DNA for 30 min at 4°C. After collecting 2% input samples, the remaining lysate was equally divided and incubated overnight with either anti-FLAG antibody (M185, MBL Life Science) or control IgG (AC011, ABclonal). Protein A+G agarose/salmon sperm DNA was then added and incubated for 2 hours at 4°C. The beads were sequentially washed with low-Salt Immune Complex Wash Buffer, High-Salt Immune Complex Wash Buffer, LiCl Immune Complex Wash Buffer, and TE buffer (twice), each for 5 min with rotation at 4°C. Precipitates were subjected to Western blotting analysis. DNA-protein complexes were eluted with elution buffer (1% SDS, 0.1 M NaHCO_3_) and reverse-cross-linked by incubation with 0.2 M NaCl at 65°C for 4 hours. Proteins were digested with proteinase K for 1 hour at 45°C, and DNA was purified using a DNA Purification Kit (D0033, Beyotime Biotechnology). The purified DNA was analyzed by qPCR and gel electrophoresis. Primers used for ChIP-PCR are listed in Supplemental Table 3.

### RLH synthesis and characterization.

DMPE-PEG2000-RLTR (RLTR) was synthesized by conjugating 1,2-dimyristoyl-*sn*-glycero-3-phosphoethanolamine–polyethylene glycol 2000–*N*-hydroxysuccinimide (DMPE-PEG2000-NHS) with the peptide RLTR (synthesized by solid-phase peptide synthesis by RuixiBiotech), following a previously reported procedure (74). Soybean phosphatidylcholine and cholesterol were dissolved in 1 mL of chloroform and evaporated under reduced pressure to form a thin lipid film in a sample vial, followed by hydration via sonication with DMPE-PEG2000-RLTR. The mixture was then treated with 180 W water bath sonication and extruded through a liposome extruder (Mini-extruder, Avanti) equipped with a polycarbonate membrane (pore size: 100 nm) to obtain blank liposomes. For RLH production, soybean phosphatidylcholine, cholesterol, and the STING inhibitor H-151 were similarly dissolved in chloroform, processed into a thin film, hydrated with DMPE-PEG2000-RLTR under sonication, and subjected to the same ultrasonication and extrusion procedures. The resulting preparation was further dialyzed using a nanodialysis device (ND-1, RuixiBiotech) to obtain purified RLH. The chemical structures of both RLTR and DMPE-PEG2000-RLTR were characterized by ^1^H-NMR spectroscopy at 400 MHz and 25°C using D_2_O as the solvent. Particle size distribution and zeta potential were measured using a NanoBrook 90Plus PALS instrument (Brookhaven Instruments). The H-151 content was measured using HPLC (EClassical3200, Elite Analytical Instruments). The morphology of RLH was imaged using a transmission electron microscope (TF20, FEI).

### Statistics.

Statistical analyses were conducted using SPSS software (version 23.0). Data are presented as mean ± SD. Comparisons between 2 groups were performed using either a 2-tailed Student’s *t* test (for normally distributed data) or Mann-Whitney *U* test (for nonnormally distributed data). For multiple-group comparisons involving normally distributed data, 1-way ANOVA was employed, followed by Bonferroni’s post hoc test (for homoscedastic data) or Tamhane’s T2 test (for heteroscedastic data). For multiple-group comparisons with nonnormally distributed data, the Kruskal-Wallis *H*-test with Dunn’s post hoc test was applied. A *P* value < 0.05 was considered statistically significant.

### Study approval.

All experiments were approved by the Medical Ethics Committee and Ethics Committee of Experimental Animal Welfare of Xijing Hospital, affiliated with the Fourth Military Medical University. All experimental procedures involving animals were approved by the Animal Care and Use Committee of the Fourth Military Medical University. The animals were treated humanely in accordance with the guidelines provided in the Guide for the Care and Use of Laboratory Animals (National Academies Press, NIH). The protocol for human sample acquisition and usage received ethical approval from the Institutional Review Board of Xijing Hospital and was conducted in accordance with the Declaration of Helsinki. Written informed consent was provided by all subjects.

### Data availability.

All data are available in the main text, supplemental material, and the Supporting Data Values file. The bulk transcriptomics (GSE335424) and single-cell transcriptomics (GSE335775) datasets generated in this study were deposited in the Gene Expression Omnibus.

## Author contributions

LW and ZBL designed the experiments and wrote the manuscript. ZBL, PZ, XYM, and JBS performed the experiments and data analysis. HZ, WD, DW, PS, XH, JL, ZQF, HX, FH, and JLD provided technical support. LW and KFD organized and supervised the study. ZBL, PZ, XYM, and JBS are co–first authors based on their distinct contributions; the order of co–first authors was determined based on the overall scientific contributions.

## Conflict of interest

The authors have declared that no conflict of interest exists.

## Funding support

- National Natural Science Foundation of China grants 82325007, 92468202, and 82421002 (to LW); 82371793 (to KFD); and 82400667 (to ZBL).
- National Key Research and Development Program of China grant 2021YFA1100502 (to LW).
- China Postdoctoral Science Foundation grants BX20240496 and 2025M774458 (to ZBL).

## Supplementary Material

Supplemental data

Unedited blot and gel images

Supporting data values

## Figures and Tables

**Figure 1:**
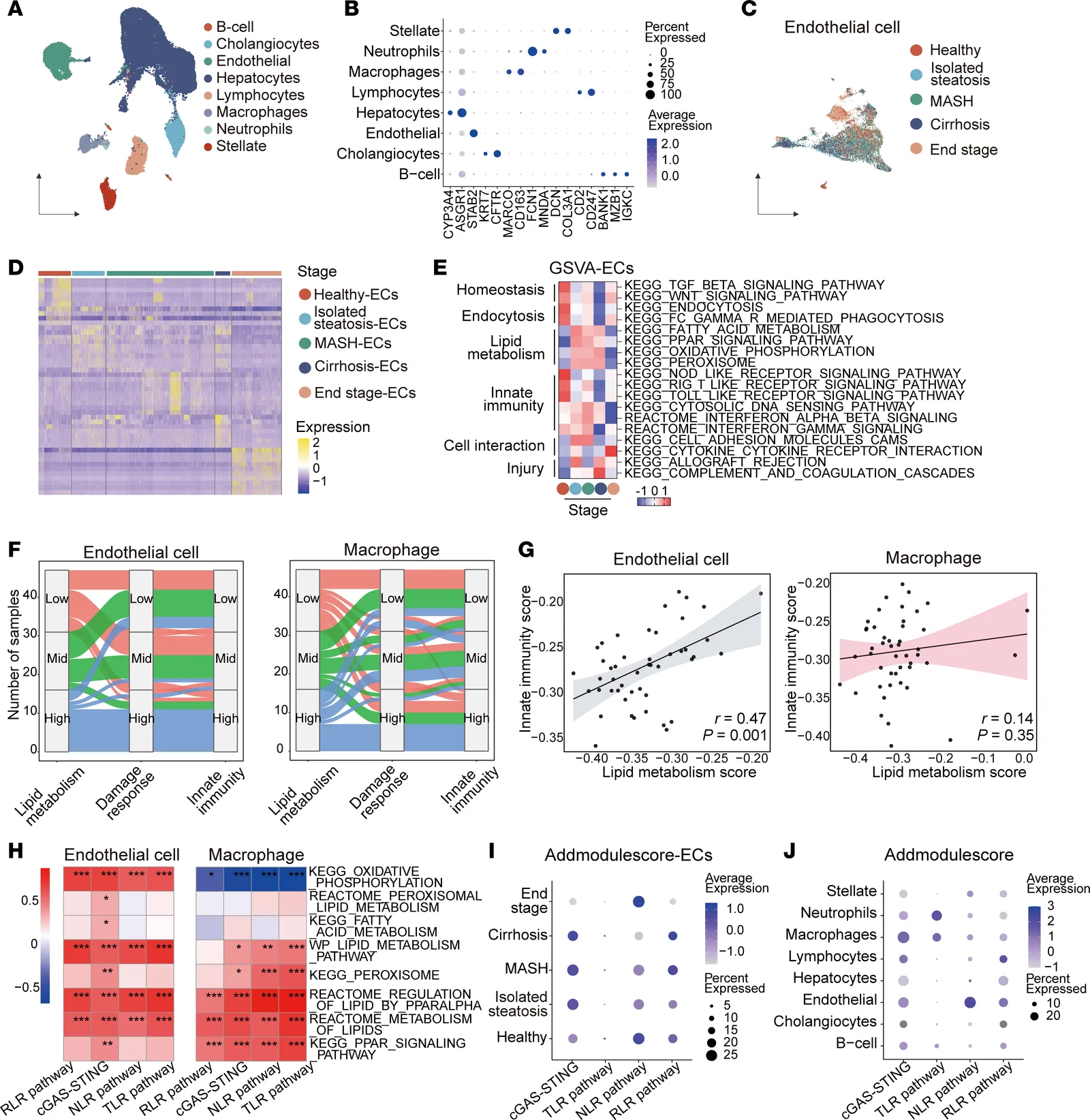
Metabolic stress selectively activates endothelial cGAS/STING signaling during MASLD progression. (**A** and **B**) Global landscape of the liver single-nucleus transcriptome (GEO: GSE202379): UMAP visualization of all integrated nuclei (**A**) and dot plot showing the expression of canonical marker genes used for cell-type annotation (**B**). (**C** and **D**) Reclustering analysis of the endothelial cell (EC) compartment: UMAP plot showing distinct EC subclusters (**C**) and gene heatmap visualizing the expression of marker genes for each EC subcluster (**D**). (**E**) Gene set variation analysis (GSVA) heatmap showing the enrichment of distinct signaling pathways in ECs across disease stages. (**F**) Alluvial plots showing sample distribution across low, medium, and high tertiles of lipid metabolism, damage response, and innate immune pathway activities (based on pseudo-bulk GSVA scores) in hepatic LSECs and macrophages. (**G**) Scatterplots showing correlations between lipid metabolism and innate immune pathway scores in LSECs and macrophages. Dots represent patient pseudo-bulk samples with linear regression lines and 95% CIs. (**H**) Heatmaps showing Spearman’s correlations between individual lipid metabolism and innate immune pathways in LSECs and macrophages, based on pseudo-bulk GSVA scores. Significance after FDR correction: *P < 0.05, **P < 0.01, ***P < 0.001. (**I**) Dot plot illustrating the activation (calculated via AddModuleScore) of 4 major innate immune signaling pathways in ECs across different MASLD stages. (**J**) Dot plot illustrating the activation (calculated via AddModuleScore) of 4 major innate immune signaling pathways across all major liver cell types.

**Figure 2:**
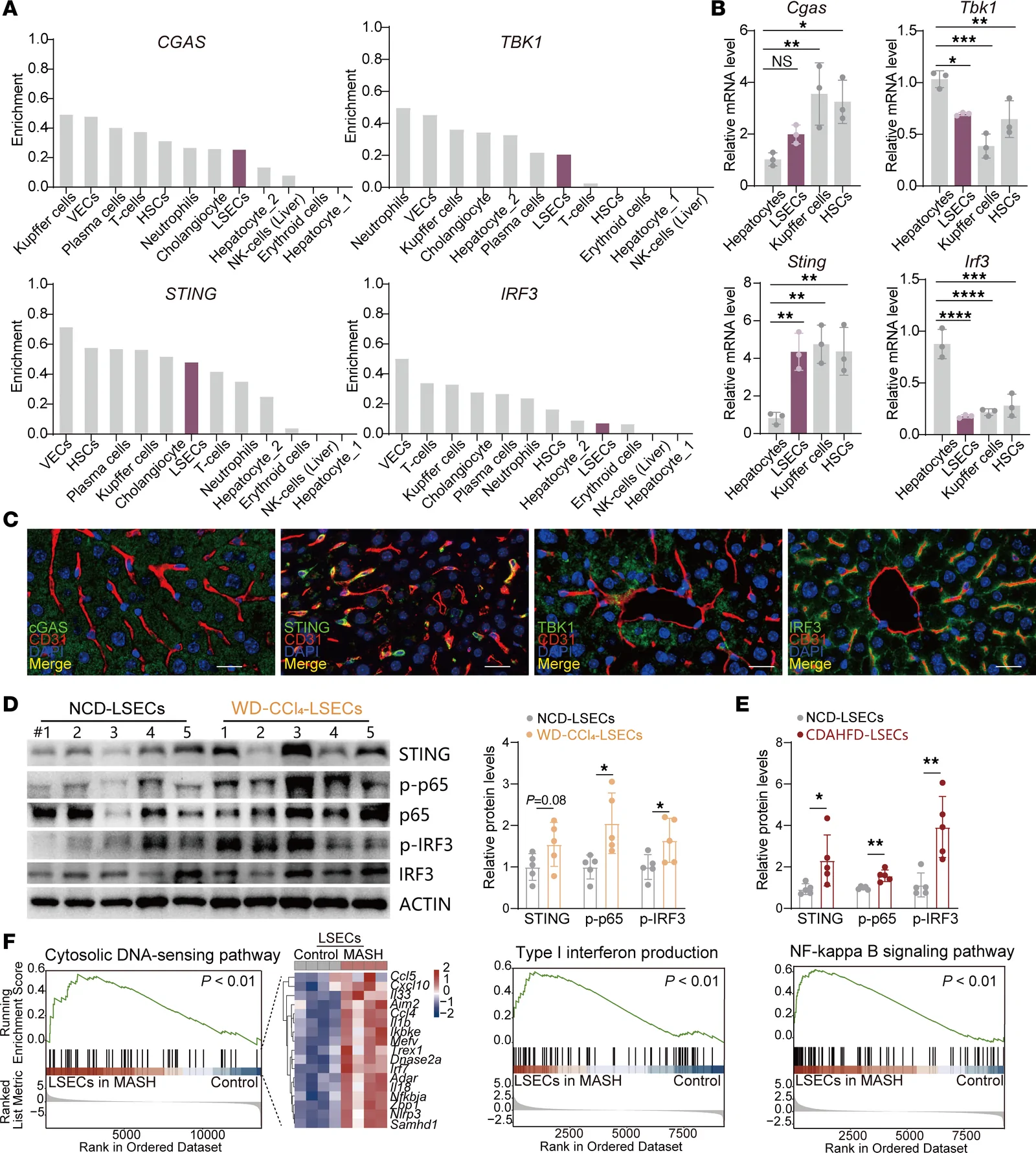
STING is endothelial cell enriched and progressively amplified during metabolic injury. (**A**) Enrichment scores of *CGAS*, *STING*, *TBK1*, and *IRF3* across indicated liver cell types; data sourced from the Human Protein Atlas. (**B**) mRNA expression levels of mouse *Cgas*, *Sting*, *Tbk1*, and *Irf3* in specific primary liver cells under physiological conditions (*n* = 3). (**C**) Representative immunofluorescence images of cGAS, STING, TBK1, and IRF3 expression in endothelial cells (CD31^+^) in human livers. Scale bars: 25 μm. The experiment was repeated 3 times. (**D** and **E**) Western blot analysis and quantification of STING, NF-κB p65, phospho-p65 (p-p65), IRF3, and p-IRF3 in LSECs isolated from the livers of mice fed either a WD-CCl_4_ for 12 weeks or a CDAHFD for 10 weeks. Mice fed a normal chow diet (NCD) served as controls (*n* = 5). (**F**) GSEA of the cytosolic DNA-sensing (cGAS/STING), type I interferon production, and NF-κB signaling pathways in LSECs from MASH liver; data derived from dataset GSE164006 (GEO). Data are mean ± SD. One-way ANOVA with Bonferroni’s post hoc analysis for **B**, **D**, and **E**. *P < 0.05, **P < 0.01, ***P < 0.001, ****P < 0.0001.

**Figure 3:**
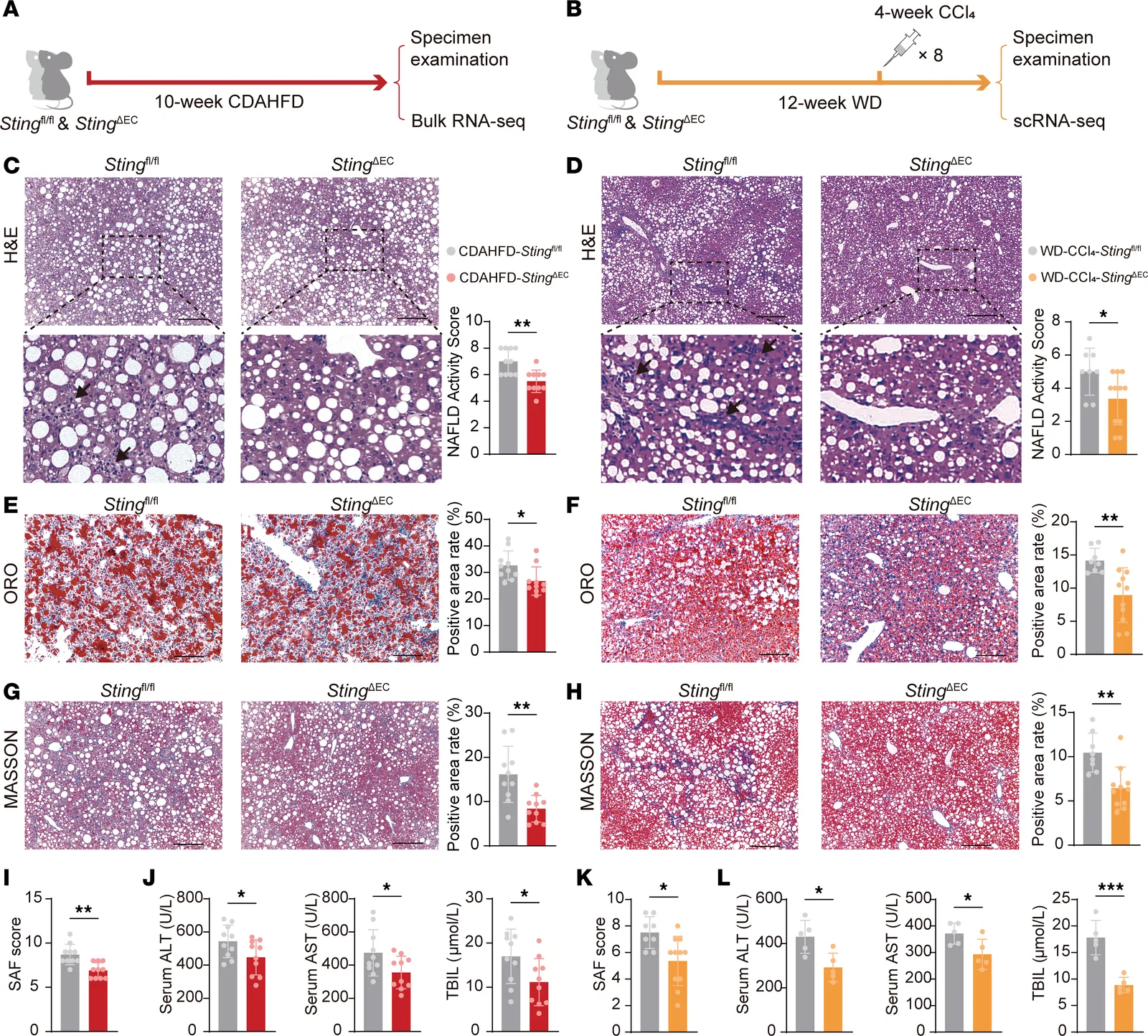
Endothelial STING is required for progression from metabolic stress to inflammatory-fibrotic niche collapse. (**A** and **B**) Schematics illustrating the experimental design for CDAHFD (**A**) and WD-CCl_4_ (**B**) induction in *Sting*^fl/fl^ and *Sting*^ΔEC^ mice. (**C** and **D**) Representative H&E-stained liver sections from *Sting*^fl/fl^ and *Sting*^ΔEC^ mice subjected to CDAHFD (*n* = 10) (**C**) and WD-CCl_4_ (*n* = 8–11) (**D**), followed by quantification using NAS. Scale bars: 200 μm. (**E** and **F**) Oil Red O (ORO) staining and quantification of liver sections from *Sting*^fl/fl^ and *Sting*^ΔEC^ mice subjected to CDAHFD (*n* = 10) (**E**) and WD-CCl_4_ (*n* = 8–11) (**F**). Scale bars: 200 μm. (**G** and **H**) Masson’s trichrome staining and quantification of liver sections from *Sting*^fl/fl^ and *Sting*^ΔEC^ mice subjected to CDAHFD (*n* = 10) (**G**) and WD-CCl_4_ (*n* = 8–11) (**H**). Scale bars: 200 μm. (**I** and **J**) Evaluation of SAF score (**I**) and liver function indices (ALT, AST, and TBIL) (**J**) in *Sting*^fl/fl^ and *Sting*^ΔEC^ mice subjected to CDAHFD (*n* = 10). (**K** and **L**) Evaluation of SAF score (*n* = 8–11) (**K**) and liver function indices (*n* = 5) (**L**) in *Sting*^fl/fl^ and *Sting*^ΔEC^ mice subjected to WD-CCl_4_. Data are mean ± SD. Mann-Whitney *U* test for **C**, **D**, **I**, and **K**; 2-tailed Student’s *t* test for other comparisons. *P < 0.05, **P < 0.01, ***P < 0.001.

**Figure 4:**
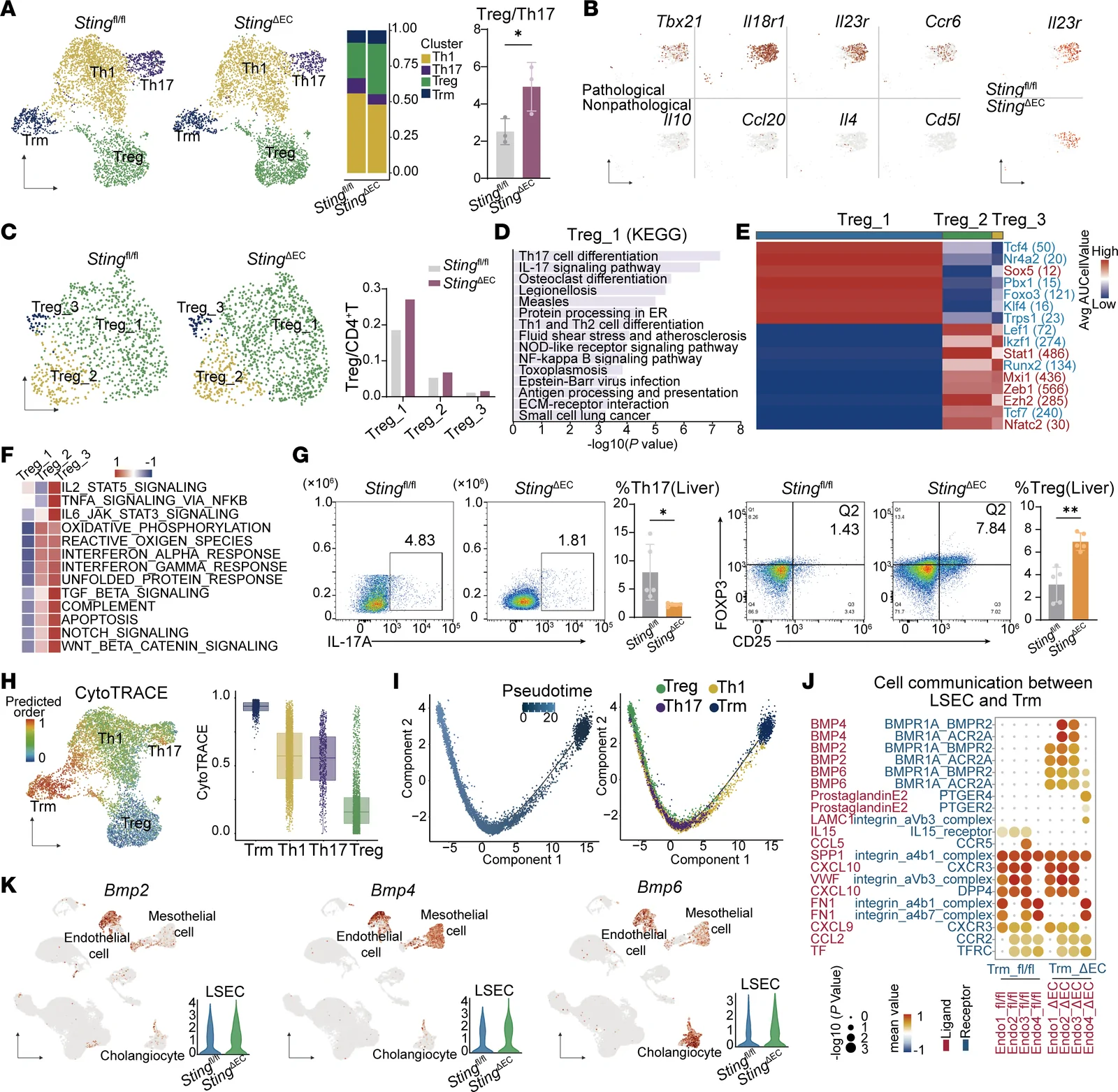
Endothelium-specific deletion of *Sting* elevates the Treg/Th17 ratio through the BMP signaling pathway. (**A**) UMAP visualization and quantification of CD4^+^ T cell subsets in livers of *Sting*^fl/fl^ and *Sting*^ΔEC^ mice subjected to WD-CCl_4_. Treg/Th17 ratios were compared between groups (*n* = 3). (**B**) UMAP plot showing the expression of pathological and nonpathological markers in Th17 from indicated groups. (**C**) UMAP visualization and quantification of identified Treg subsets. (**D**) KEGG pathway enrichment analysis of differentially expressed genes in the Treg_1 subset. (**E**) SCENIC analysis showing the regulatory activity of TFs across Treg subsets. **(F)** QuSAGE analysis of gene set activity in Treg subsets. (**G**) Flow cytometry measurement of Th17 and Treg proportions in livers of *Sting*^fl/fl^ and *Sting*^ΔEC^ mice subjected to WD-CCl_4_ (*n* = 5). (**H** and **I**) Differentiation trajectories of CD4^+^ T cell subsets inferred through CytoTRACE (**H**) and pseudotime analysis (**I**). (**J**) CellPhoneDB analysis of ligand–receptor interaction intensity between LSECs and Trm cells. (**K**) UMAP plots showing *Bmp2*, *Bmp4*, and *Bmp6* expression across all hepatic cell types in the 2 groups. Data are mean ± SD. Two-tailed Student’s *t* test for all comparisons. *P < 0.05, **P < 0.01.

**Figure 5:**
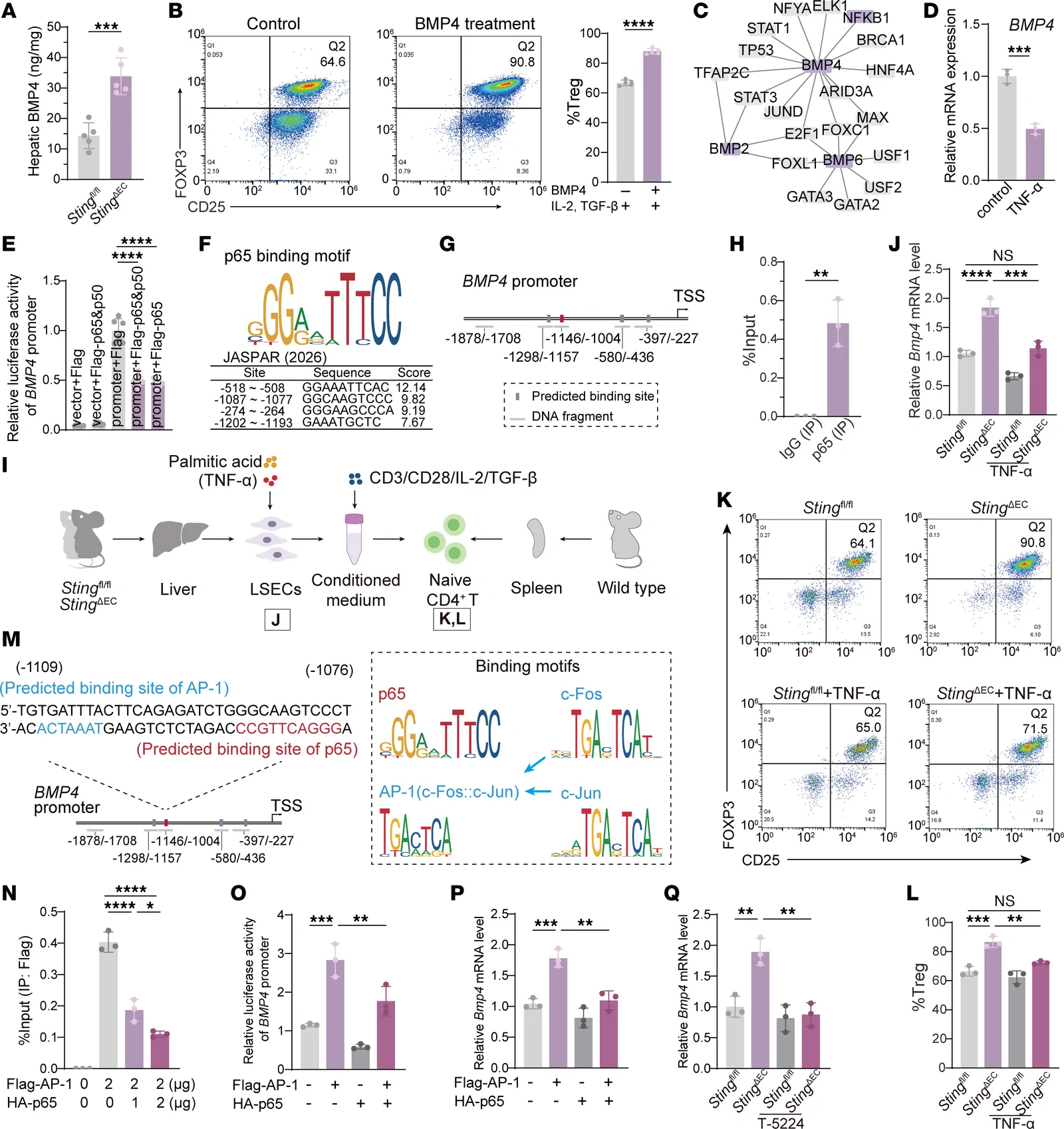
STING/NF-κB suppresses Treg skewing by blocking AP-1–mediated BMP4 transcription. (**A**) ELISA detecting BMP4 protein in *Sting*^fl/fl^ and *Sting*^ΔEC^ livers (*n* = 5). (**B**) FACS analysis of Treg differentiation from primary naive CD4^+^ T cells induced by indicated cytokines (*n* = 5). (**C**) TFs of *BMP2/4/6* predicted by the NetworkAnalyst platform. (**D**) qPCR analyzing *Bmp4* transcription in primary LSECs treated with TNF-α or vehicle (*n* = 3). (**E**) Dual-luciferase reporter assay evaluating transcriptional regulation of *BMP4* by NF-κB subunits (*n* = 6). (**F**) Predicted p65 binding motif and sites within the *BMP4* promoter using the JASPAR database. (**G**) Schematic of predicted p65 binding sites and targeted primers. (**H**) ChIP-qPCR validating p65 binding to the *BMP4* promoter (fragment –1,146 to –1,004) (*n* = 3). (**I**) Schematic of LSEC-mediated Treg differentiation. (**J**) qPCR analyzing *Bmp4* transcription in LSECs from *Sting*^fl/fl^ and *Sting*^ΔEC^ mice treated with TNF-α or vehicle (*n* = 3). (**K** and **L**) FACS and quantification of Treg differentiation induced by the indicated LSEC-conditioned medium (*n* = 3). (**M**) Predicted binding motifs and sites of p65 and AP-1 (including c-Fos and c-Jun) within the *BMP4* promoter using the JASPAR database. (**N**) ChIP-qPCR detection of AP-1 binding to the *BMP4* promoter (fragment –1,146 to –1,004) with or without p65 (*n* = 3). (**O**) Dual-luciferase reporter assay evaluating transcriptional regulation of *BMP4* by AP-1 with or without p65 (*n* = 3). (**P**) qPCR analyzing *Bmp4* transcription in endothelial cells transfected with AP-1 and/or p65 (*n* = 3). (**Q**) qPCR analyzing *Bmp4* transcription in LSECs from *Sting*^fl/fl^ and *Sting*^ΔEC^ mice treated with T-5224 or vehicle (*n* = 3). Data are mean ± SD. Two-tailed Student’s *t* test for **A**, **B**, **D**, and **H**; 1-way ANOVA with Bonferroni’s post hoc analysis for other comparisons. *P < 0.05, **P < 0.01, ***P < 0.001, ****P < 0.0001.

**Figure 6:**
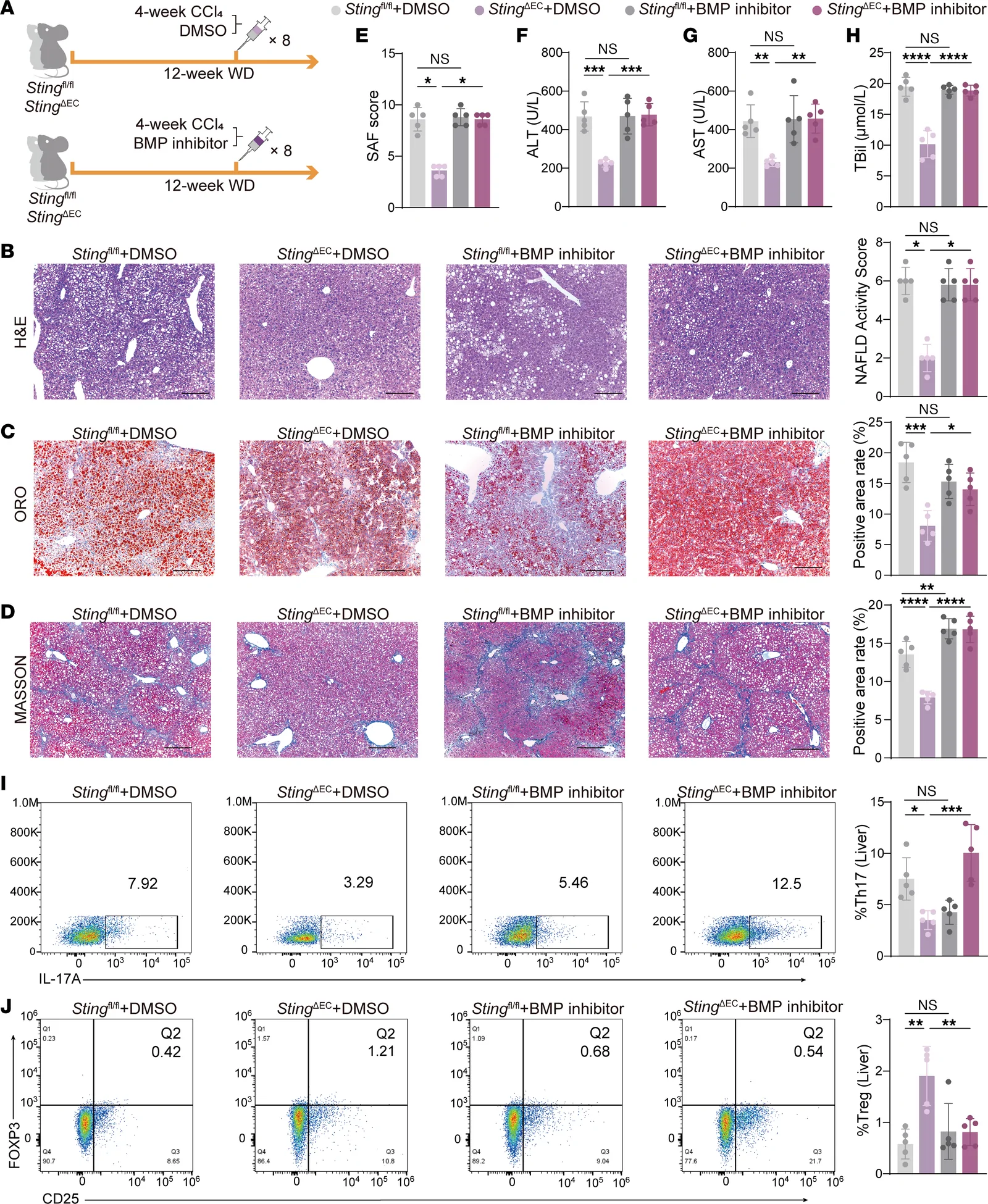
Inhibition of BMP signaling abrogated the protective effects conferred by endothelium-specific *Sting* knockout. (**A**) Schematic of the rescue experiment: WD-CCl_4_ administration in *Sting*^fl/fl^ and *Sting*^ΔEC^ mice, with or without treatment with the BMP signaling inhibitor LDN-193189. (**B**–**E**) H&E (**B**), Oil Red O (ORO; **C**), and Masson’s trichrome (**D**) staining of liver sections from the 4 groups, followed by NAS and SAF scoring (**E**; *n* = 5). Scale bars: 200 μm. (**F**–**H**) Serum liver function indices from the 4 groups: ALT (**F**), AST (**G**), and TBIL (**H**; *n* = 5). (**I** and **J**) Flow cytometry analysis of Th17 (**I**) and Treg proportions (**J**) in livers from the 4 groups (*n* = 5). Data are mean ± SD. Kruskal-Wallis *H*-test with Dunn’s post hoc test for **B** and **E**; 1-way ANOVA with Bonferroni’s post hoc analysis for other comparisons. *P < 0.05, **P < 0.01, ***P < 0.001, ****P < 0.0001.

**Figure 7:**
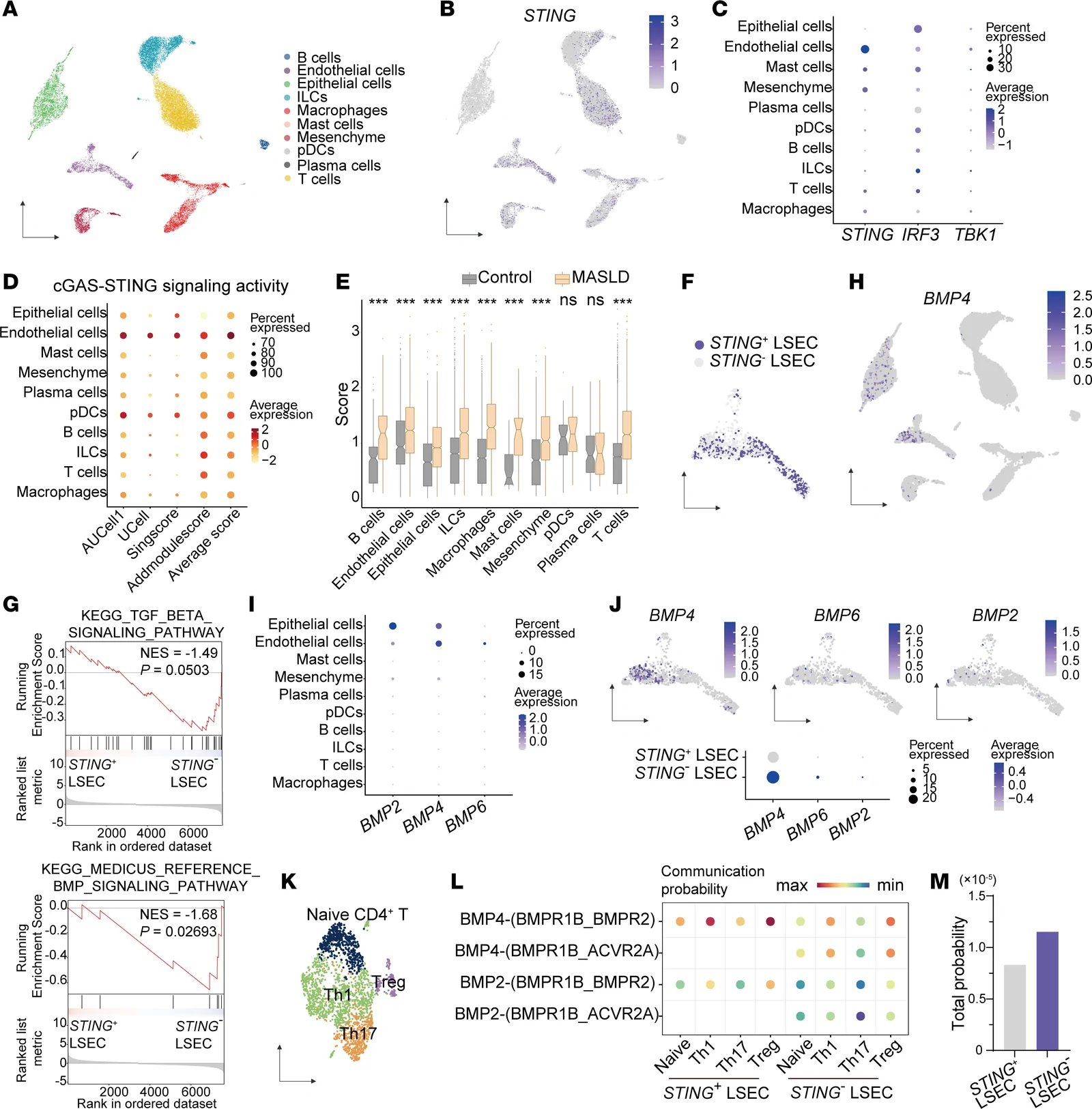
Human single-cell analysis validates a conserved endothelial STING-BMP4 angiocrine checkpoint. (**A**) UMAP visualization of all cell clusters from human liver scRNA-seq data (GEO: GSE136103). (**B**) UMAP plot showing *STING* expression across all cell types. (**C**) Dot plot showing the expression of *STING*, *IRF3*, and *TBK1* across all cell types. (**D**) Evaluation of cGAS/STING signaling pathway activation across all cell types using 4 functional scores and their average. (**E**) Average cGAS/STING pathway activation scores in various cell types from healthy and MASLD livers. (**F**) UMAP plot showing *STING* expression specifically in endothelial cells. (**G**) GSEA of TGF-β and BMP signaling pathway activation in *STING***^–^** versus *STING*^+^ endothelial cells. (**H**) UMAP plot showing *BMP4* expression across all cell types. (**I** and **J**) Dot plots showing the expression of *BMP2*, *BMP4*, and *BMP6* in all cell types (**I**) and specifically in endothelial cells (**J**). (**K**) UMAP visualization of CD4^+^ T cell clusters. (**L** and **M**) CellChat analysis showing BMP signaling interactions between *STING***^–^** or *STING*^+^ endothelial cells and CD4^+^ T cells (**L**) along with the overall interaction score (**M**). ***P < 0.001. (**E**) Wilcoxon test. (**G**) Permutation test.

**Figure 8:**
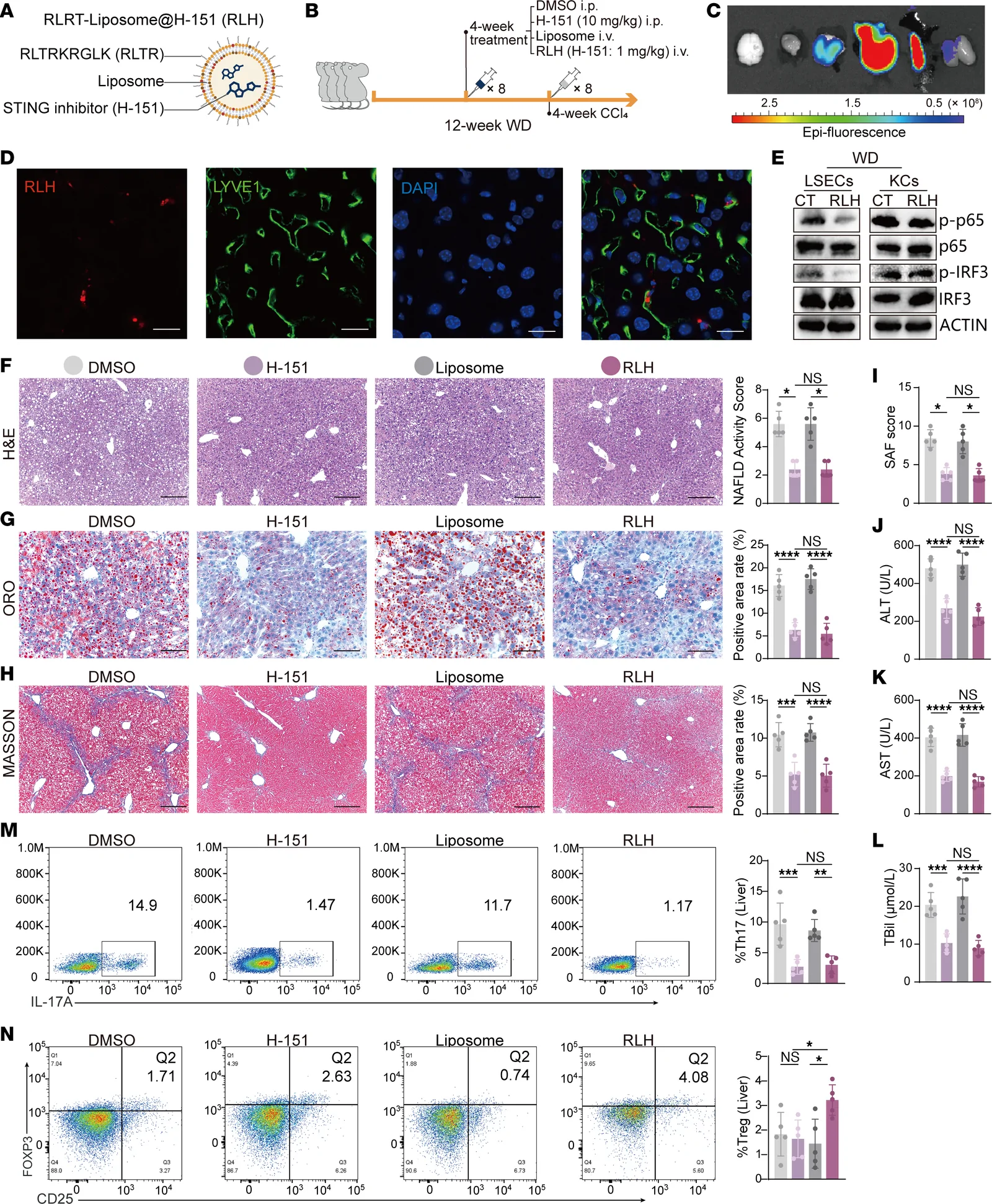
Delivery of a STING inhibitor via LSEC-directed RLTR-liposome ameliorates MASH progression. (**A**) Schematic illustrating the construction of RLH nanoparticles. (**B**) Schematic of the therapeutic experiments using H-151 and RLH. (**C** and **D**) In vivo distribution of DiD perchlorate–labeled RLH in major organs monitored by fluorescence imaging (**C**) and immunofluorescence verification of RLH uptake by LSECs (**D**). Scale bar: 20 μm. (**E**) Western blot analysis of p65, phospho-p65 (p-p65), IRF3, and p-IRF3 in LSECs and KCs isolated from the WD-fed mice treated with RLH or empty liposome (CT). (**F**–**I**) H&E (**F**), Oil Red O (ORO; **G**), and Masson’s trichrome (**H**) staining of liver sections from the 4 groups, followed by NAS and SAF scoring (**I**; *n* = 5). Scale bar: 100 μm (**G**), 200 μm (**F** and **H**). (**J**–**L**) Serum liver function indices from the 4 groups: ALT (**J**), AST (**K**), and TBIL (**L**; *n* = 5). (**M** and **N**) Flow cytometry analysis of the effects of the 4 treatments on hepatic Th17 (**M**) and Treg cell proportions (*n* = 5) (**N**). Data are mean ± SD. Kruskal-Wallis *H*-test with Dunn’s post hoc test for **F** and **I**; 1-way ANOVA with Bonferroni’s post hoc analysis for other comparisons. *P < 0.05, **P < 0.01, ***P < 0.001, ****P < 0.0001. The experiments for **C**–**E** were each repeated 3 times.

## Version 1 08/12/2026

In-Press Preview

## Version 2 10/01/2026

Electronic publication
