# Supplementary material for: Metabolic stress–activated endothelial STING rewires the sinusoidal immune niche to promote MASLD progression
Source: J Clin Invest. 2026 Aug 6;136(19):e207031. doi: 10.1172/JCI207031 (PMC13626822; doi:10.1172/JCI207031)

Supplemental Materials for

# Metabolic stress-activated endothelial STING rewires the sinusoidal immune niche to promote MASLD progression

Zhibin Lin *et al.*

* **Co-corresponding authors:** Kefeng Dou: doukef@fmmu.edu.cn; Lin Wang: fierywang@163.com

**This PDF file includes:**

Supplemental figure 1 to 13

Supplemental methods

Supplemental table 1 to 4

Gating strategy of flow cytometry

## Supplemental figures

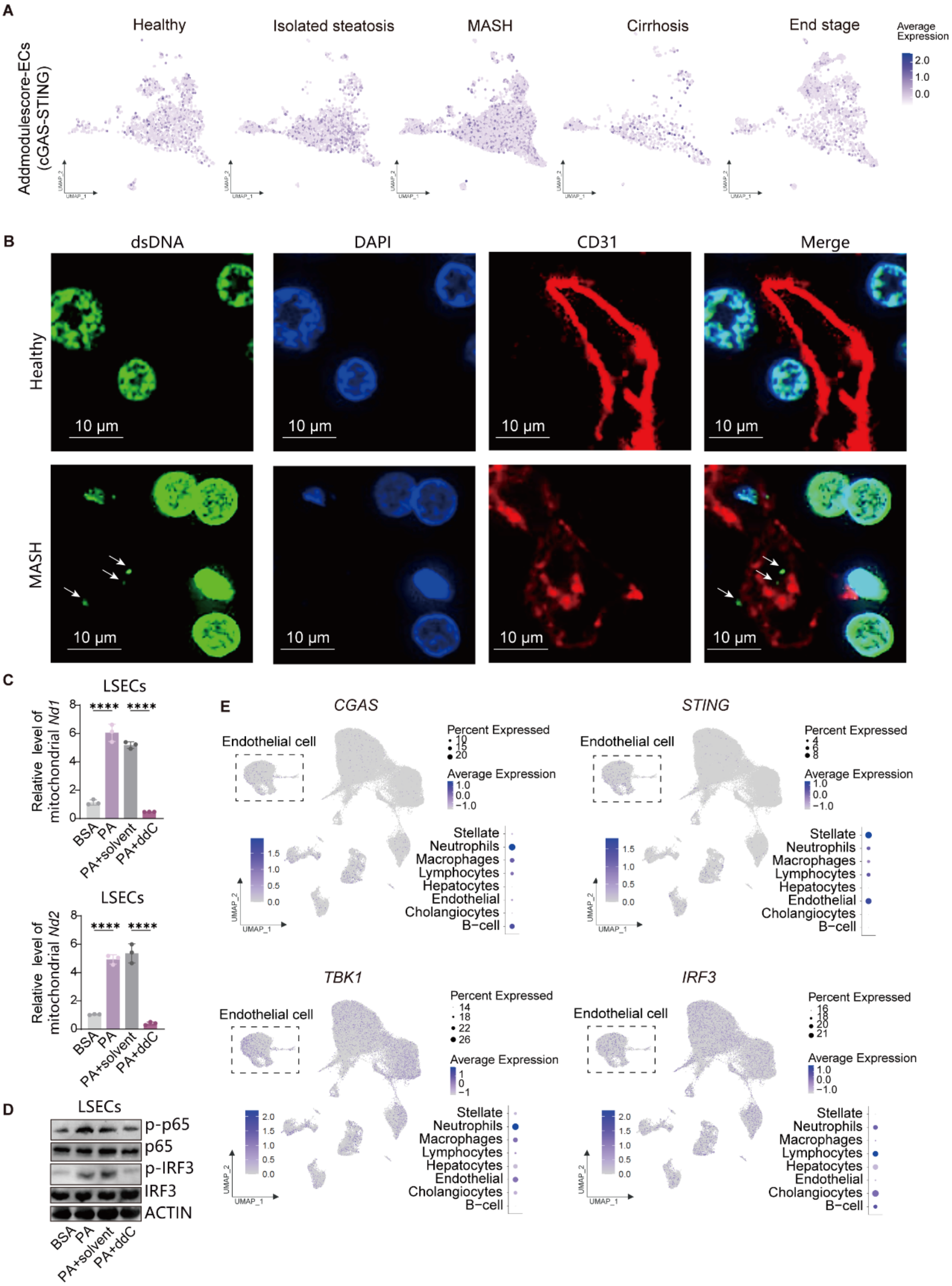


**Supplemental Figure 1. Metabolic stress activates endothelial cGAS-STING signaling during MASLD progression. A,** UMAP plots showing the activation level

of cytosolic DNA-sensing pathway in liver endothelial cells across different stages of MASLD (data sourced from the GSE202379 dataset). **B,** Representative immunofluorescence images of double-stranded DNA (dsDNA) in hepatic sinusoids of healthy and MASH human livers (scale bar: 10 μm). **C,** qPCR detecting the relative level of mitochondrial *Nd1* and *Nd2* in endothelial cells treated with palmitic acid (PA), bovine serum albumin (BSA), or 2',3'-Dideoxycytidine (ddC). **D,** Western blot analysis of p65, phospho-p65 (p-p65), IRF3, and p-IRF3 in endothelial cells treated with palmitic acid (PA), bovine serum albumin (BSA), or 2',3'-Dideoxycytidine (ddC). **E,** UMAP feature plots and dot plots visualizing the mRNA expression levels of key cGAS-STING pathway components (*CGAS*, *STING*, *TBK1*, and *IRF3*) across identified cell clusters. Data are mean ± s.d. One-way ANOVA with Bonferroni post hoc analysis for all comparisons. ****$P$ < 0.0001. The experiments for **B** and **D** were each repeated three times.

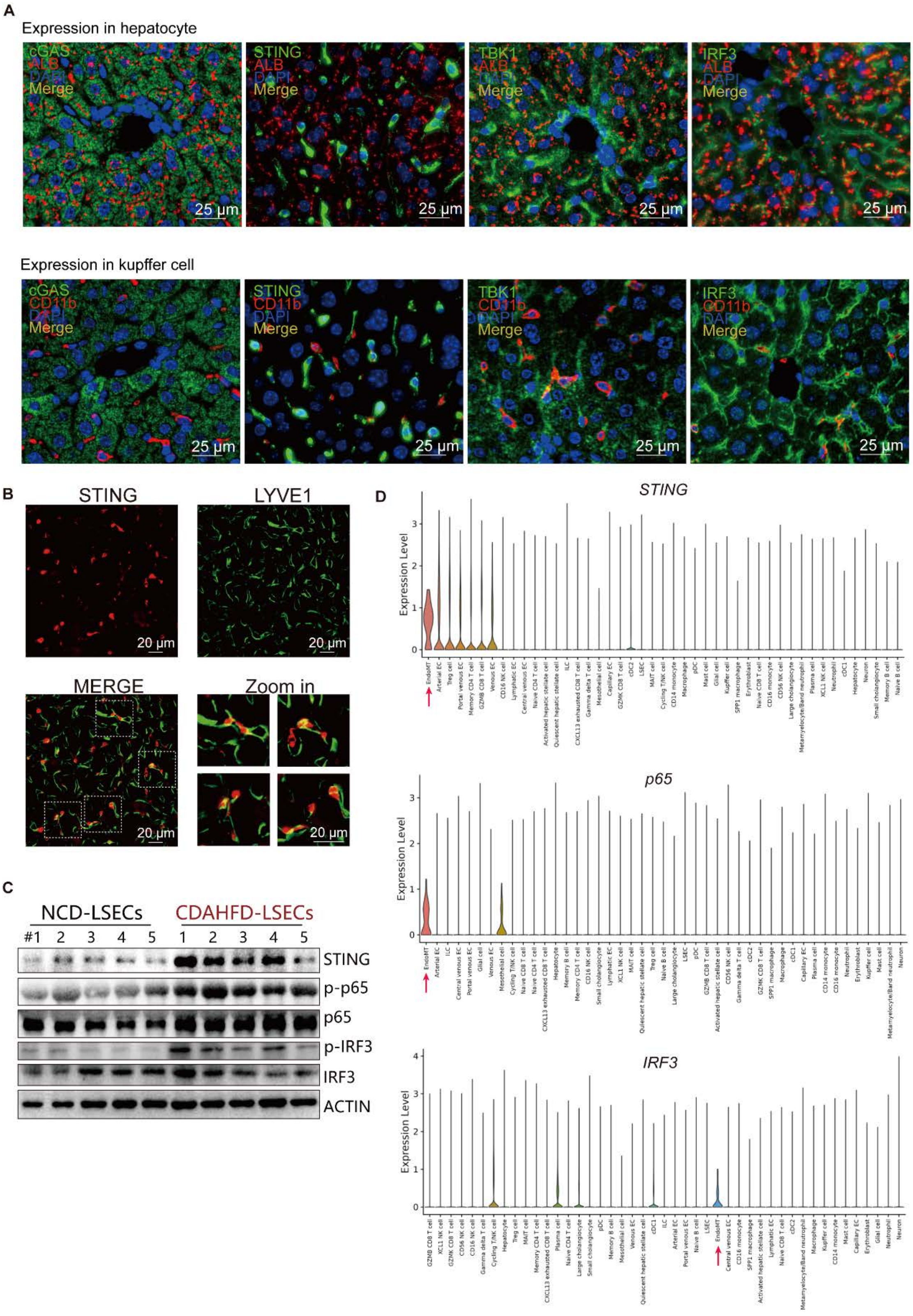


**Supplemental Figure 2. STING expression in human liver. A,** Representative immunofluorescence images of cGAS, STING, TBK1 and IRF3 expression in

hepatocyte ($ALB^+$) and Kupffer cell ($CD11b^+$) in human livers (scale bar: 25 μm). **B**, Representative IF staining of STING and LYVE1 (a marker for LSECs) in mouse liver under physiological conditions (scale bar: 20 μm). **C**, Western blot analysis of STING, NF-κB p65, phospho-p65 (p-p65), IRF3, and p-IRF3 in LSECs isolated from the livers of mice fed a choline-deficient, L-amino acid-defined, high-fat diet (CDAHFD) for 10 weeks. Mice fed a normal chow diet (NCD) served as controls ($n$ = 5). **D,** Expression profiles of *STING*, *RELA* (p65), and *IRF3* across various cell types (data sourced from the DISCO database). The experiments for **A** and **B** were each repeated three times.

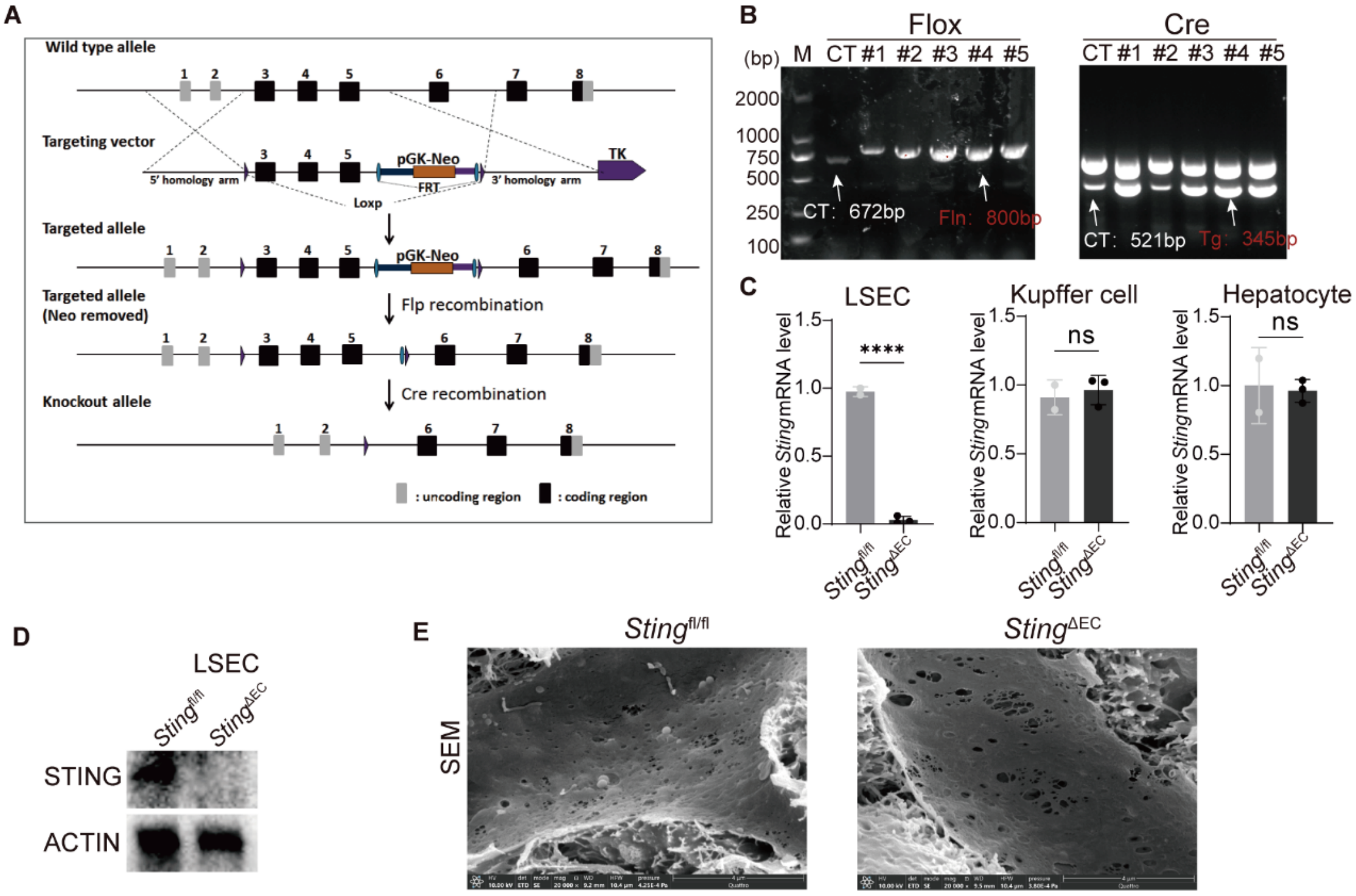


**Supplemental Figure 3. Generation and validation of endothelial-specific *Sting* knockout mice. A,** Schematic strategy for generating *Sting*$^{\Delta EC}$ mice. **B–D,** Validation of knockout efficiency at the genomic DNA (**B**), mRNA (**C**), and protein (**D**) levels in the indicated cells isolated from the livers of *Sting*$^{fl/fl}$ and *Sting*$^{\Delta EC}$ mice. **E,** Representative scanning electron microscopy (SEM) images showing liver sinusoidal endothelial fenestrae structures in *Sting*$^{fl/fl}$ and *Sting*$^{\Delta EC}$ mice under physiological conditions (scale bar: 4 μm). Data are mean ± s.d. Two-tailed Student's *t*-test for **C**. ****$P < 0.0001$; ns, not significant. The experiments for **D** and **E** were each repeated three times.

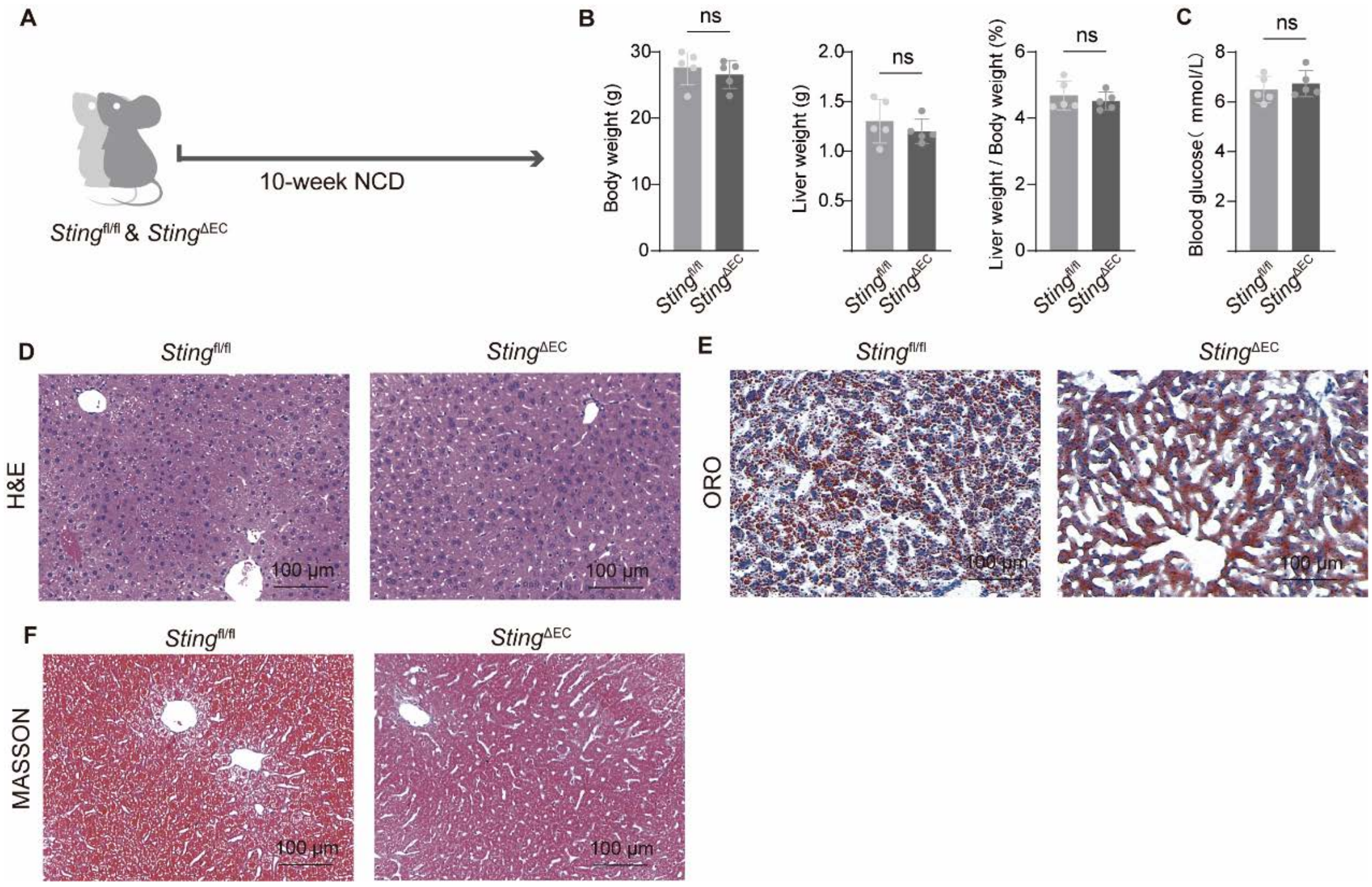


**Supplemental Figure 4. Phenotypic characterization of *Sting*$^{\Delta EC}$ mice under a normal chow diet. A,** Schematic showing the 10-week normal chow diet (NCD) feeding regimen. **B, C,** Changes in body weight, liver weight, liver-to-body weight ratio (**B**), and blood glucose levels (**C**) in the two groups. **D–F,** H&E (**D**), Oil Red O (**E**), and Masson's trichrome (**F**) staining of liver sections (scale bar: 100 μm). Data are mean ± s.d. Two-tailed Student's *t*-test. ns, not significant. The experiments for **D-F** were each repeated three times.

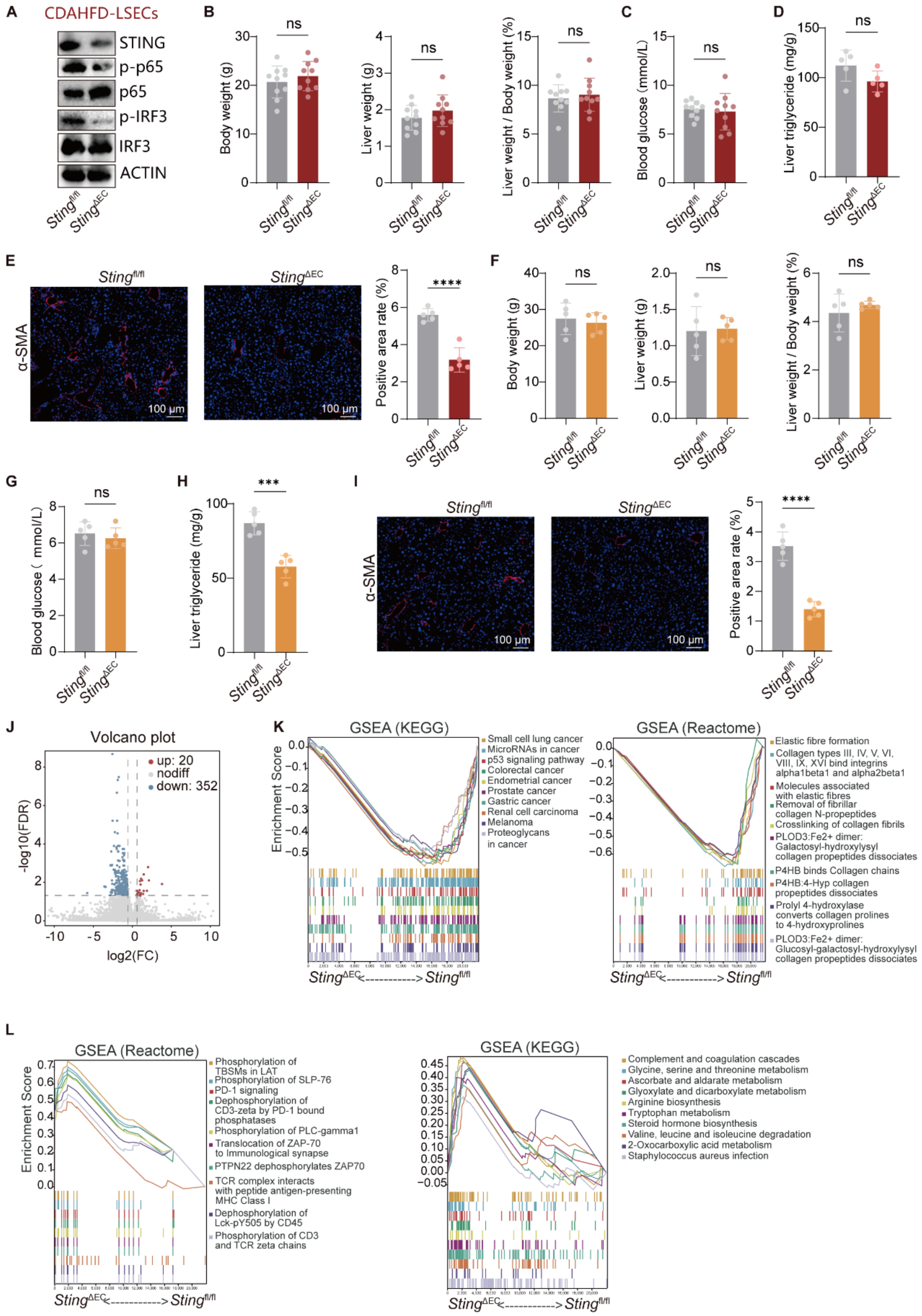

**Supplemental Figure 5. Phenotypic changes in *Sting*$^{\Delta EC}$ mice under MASH-inducing diets. A,** Western blot analysis of STING, p65, phospho-p65 (p-p65), IRF3,

and p-IRF3 in LSECs isolated from the livers of *Sting*$^{fl/fl}$ and *Sting*$^{\Delta EC}$ mice fed a CDAHFD. The experiment was repeated three times. **B, C,** Body weight, liver weight, liver-to-body weight ratio (**B**), and blood glucose (**C**) in mice fed a CDAHFD for 10 weeks. **D,** Levels of liver triglycerides in the livers of *Sting*$^{fl/fl}$ and *Sting*$^{\Delta EC}$ mice fed a CDAHFD. **E,** Representative immunofluorescence images and quantification of alpha-smooth muscle actin (α-SMA) expression in the livers of *Sting*$^{fl/fl}$ and *Sting*$^{\Delta EC}$ mice fed a CDAHFD (scale bar: 100 μm). **F, G,** Body weight, liver weight, liver-to-body weight ratio (**F**), and blood glucose (**G**) in mice induced with WD-$CCl_4$ for 12 weeks. **H,** Levels of liver triglycerides in the livers of *Sting*$^{fl/fl}$ and *Sting*$^{\Delta EC}$ mice induced with WD-$CCl_4$. **I,** Representative immunofluorescence images and quantification of alpha-smooth muscle actin (α-SMA) expression in the livers of *Sting*$^{fl/fl}$ and *Sting*$^{\Delta EC}$ mice induced with WD-$CCl_4$ (scale bar: 100 μm). **J,** Volcano plot of differentially expressed genes (DEGs) from bulk RNA-seq of livers from CDAHFD-fed *Sting*$^{fl/fl}$ and *Sting*$^{\Delta EC}$ mice. **K, L,** GSEA showing the top 10 enriched KEGG and Reactome pathways in livers of *Sting*$^{fl/fl}$ mice (**K**) and *Sting*$^{\Delta EC}$ mice (**L**), $n = 3$. Data are mean ± s.d. Two-tailed Student's *t*-test for all comparisons. ***$P < 0.001$; ****$P < 0.0001$; ns, not significant.

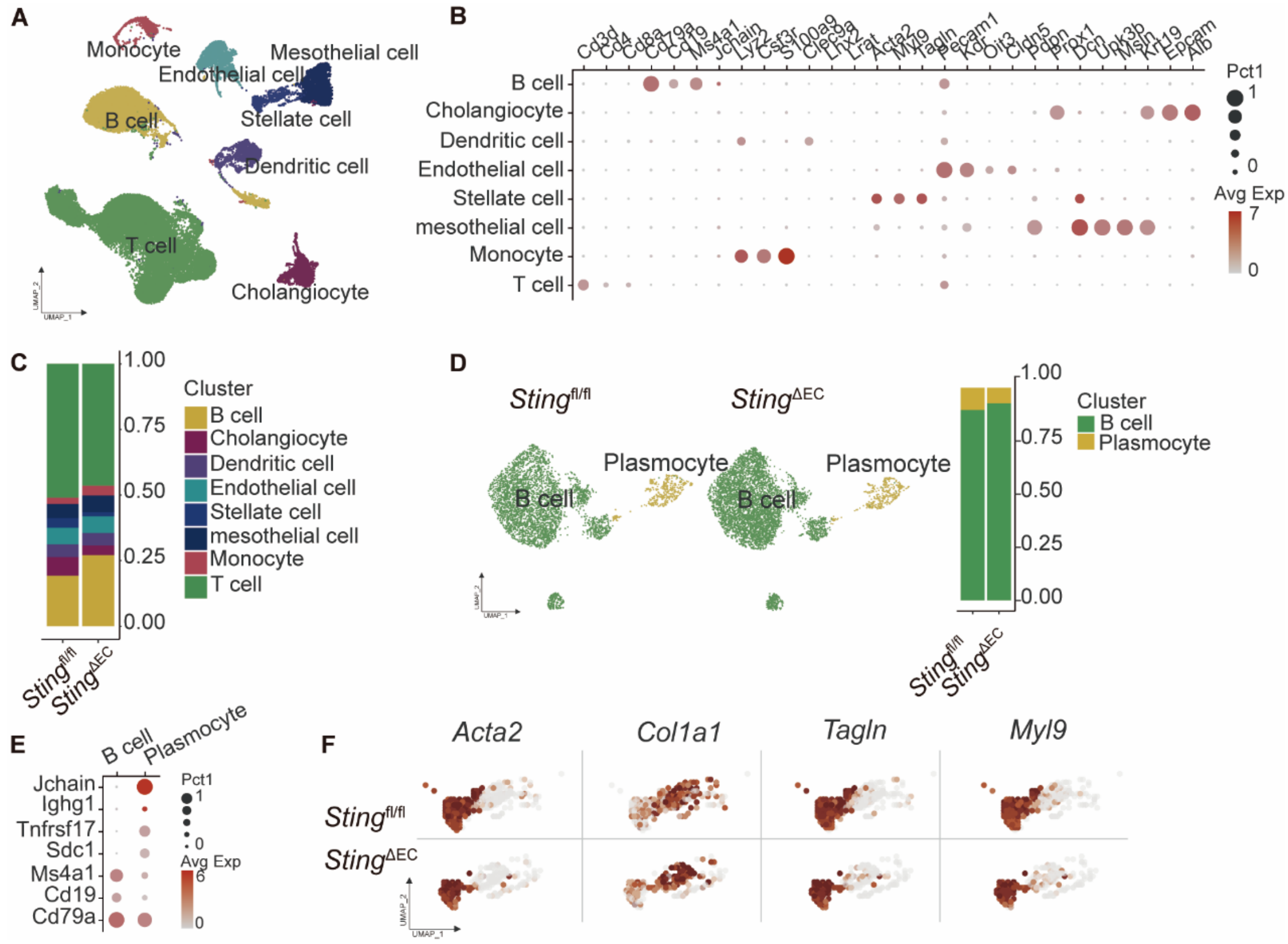


**Supplemental Figure 6. scRNA-seq analysis of B cells and hepatic stellate cells in WD-CCl4-induced mice. A, B,** UMAP visualization of all cell clusters (**A**) and expression of corresponding marker genes (**B**). **C,** Changes in the proportions of various cell types following endothelial *Sting* deletion. **D, E,** Proportions of B cells and plasma cells (**D**) and expression of their respective markers (**E**). **F,** Expression changes of fibrosis-associated genes in hepatic stellate cells following endothelial *Sting* deletion.

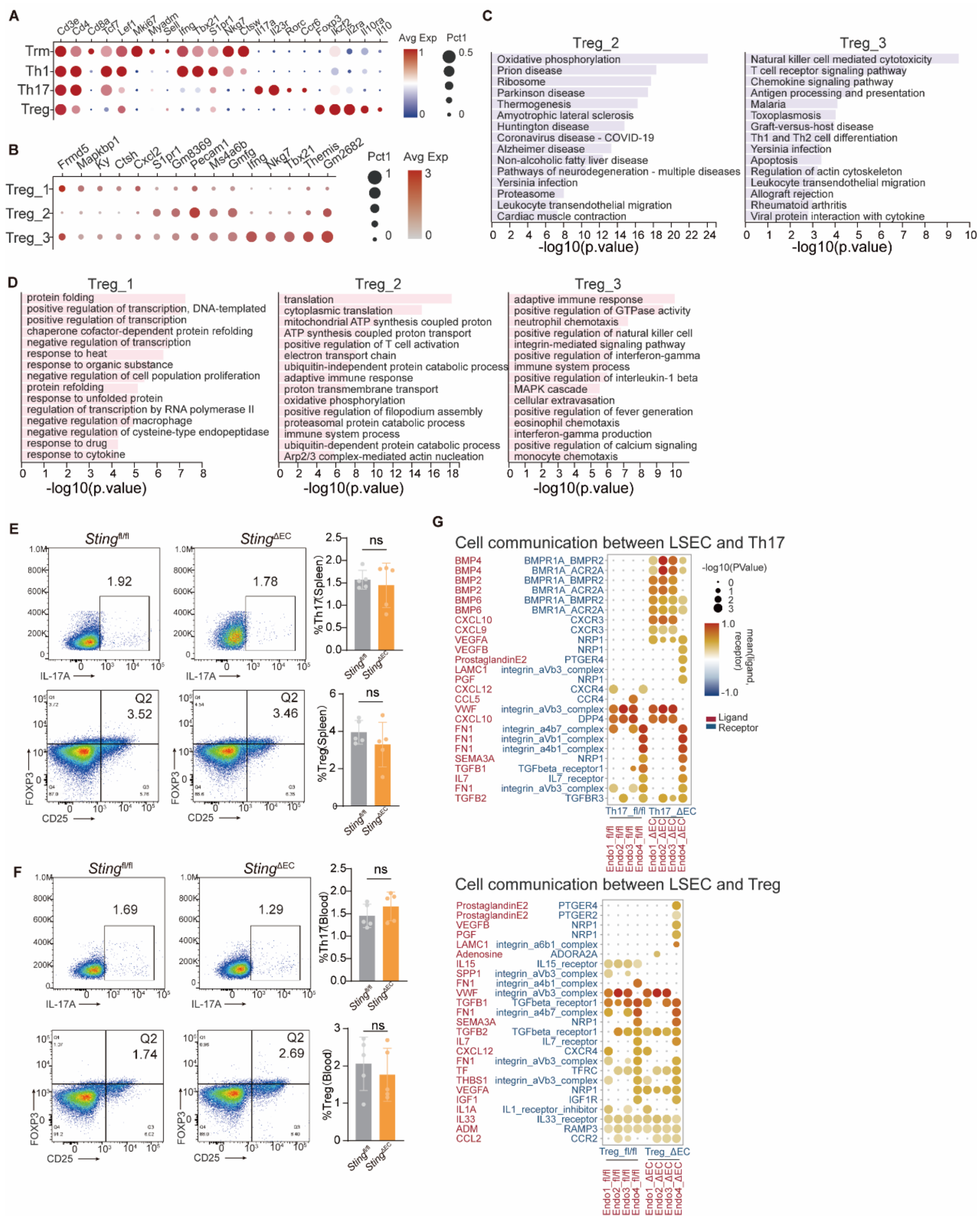

**Supplemental Figure 7. scRNA-seq analysis of CD4⁺ T cells in WD-CCl₄-induced mice. A,** Expression of marker genes for four CD4⁺ T cell clusters. **B–D,** Gene expression profiles (**B**), KEGG pathway enrichment (**C**), and GO Molecular Function (GO-MF) enrichment (**D**) of the three Treg subsets. **E, F,** Flow cytometric analysis of Th17 and Treg proportions in the spleen (**E**) and peripheral blood (**F**) of WD-CCl₄-

induced mice. **G,** CellPhoneDB analysis of ligand–receptor interaction intensity between LSECs and Th17 cells or Tregs. Data are mean ± s.d. Two-tailed Student's *t*-test for all comparisons. ns, not significant.

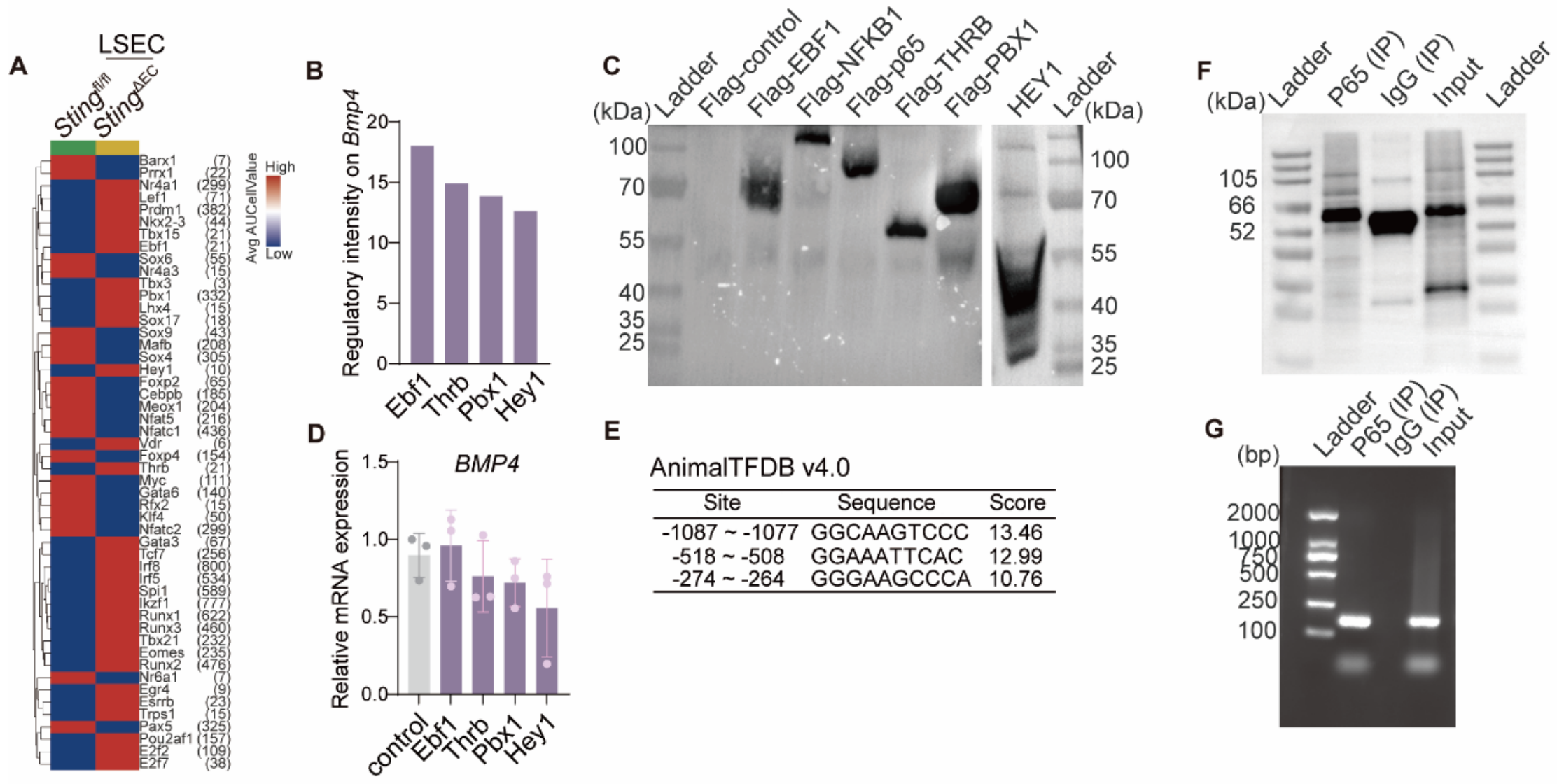


**Supplemental Figure 8. Transcriptional regulation of *Bmp4* in LSECs. A,** SCENIC analysis of transcription factor activity in endothelial cells from *Sting*$^{fl/fl}$ and *Sting*$^{\Delta EC}$ mice based on scRNA-seq data. **B,** Activity of four transcription factors highly correlated with *Bmp4* expression. **C,** Construction and validation of plasmids overexpressing *Bmp4*-related transcription factors. **D,** qPCR analysis of *BMP4* transcription in 293T cells transfected with the indicated transcription factors. **E,** Predicted p65 candidate binding sites within the human *BMP4* promoter, based on AnimalTFDB. **F,** ChIP-Western blot validation using anti-Flag antibody in 293T cells transfected with Flag-p65. **G,** Agarose gel electrophoresis of ChIP-qPCR products. The experiments for **C**, **F** and **G** were each repeated three times.

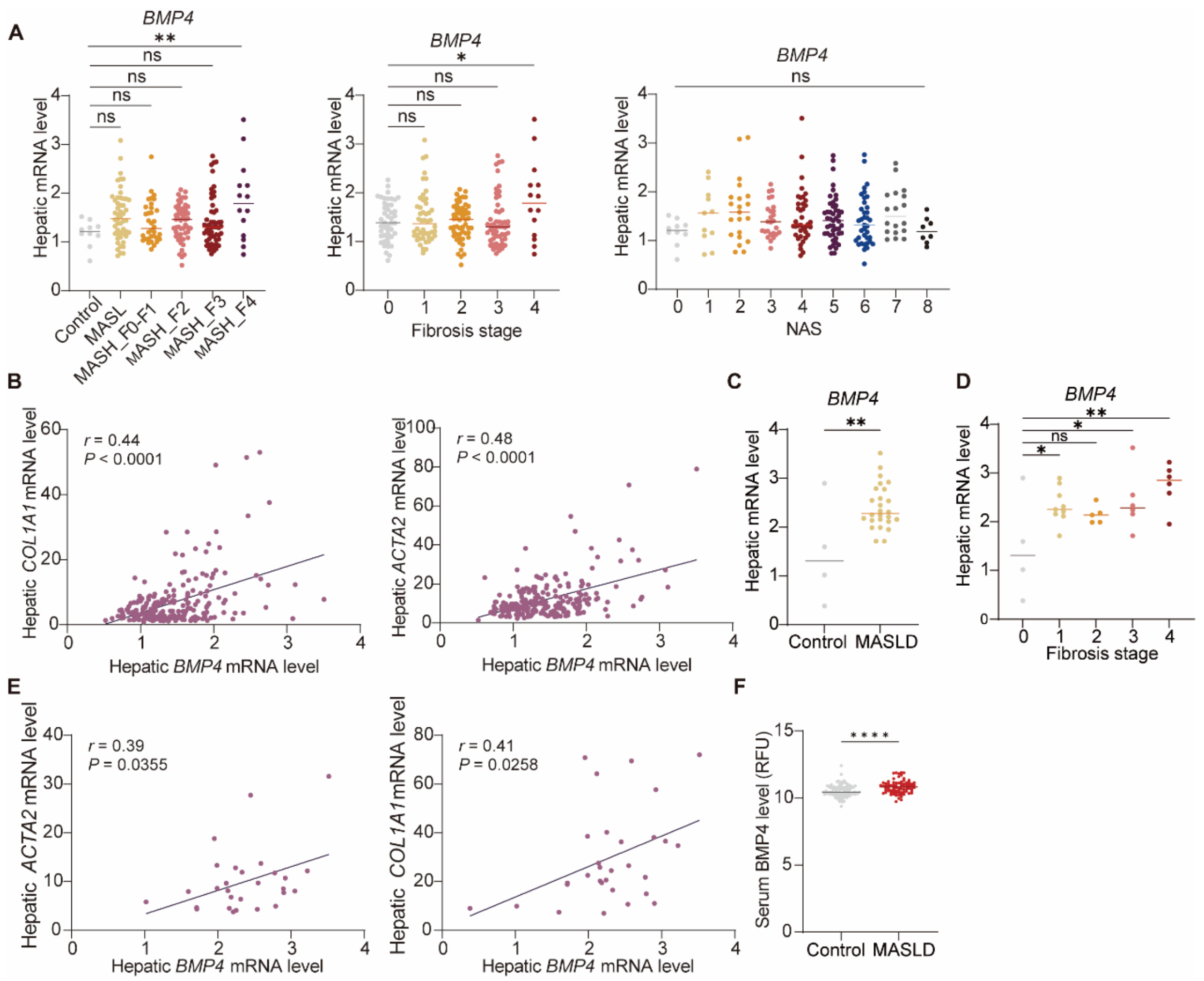


**Supplemental Figure 9. Upregulation of *Bmp4* expression in human MASH livers.**

**A,** Hepatic *BMP4* mRNA levels across different fibrosis stages or NAS scores in human MASLD (data from GSE135251). **B,** Correlation analysis between hepatic *BMP4* mRNA and *COL1A1* or *ACTA2* mRNA expression in the GSE135251 dataset. **C,** Hepatic *BMP4* mRNA levels in healthy controls and patients with MASLD (data from GSE246221). **D,** Hepatic *BMP4* mRNA levels across different fibrosis stages in MASLD (data from GSE246221). **E,** Correlation analysis between hepatic *BMP4* mRNA and *COL1A1* or *ACTA2* mRNA expression (data from GSE246221). **F,** Serum BMP4 protein levels in healthy controls and patients with MASLD (data sourced from *Nature Metabolism* 2023, 10.1038/s42255-023-00775-1). Data are mean ± s.d. Two-tailed Student's *t*-test for **C** and **F**; Pearson correlation analysis for **B** and **E**; one-way

ANOVA with Bonferroni post hoc analysis for other comparisons. *$P < 0.05$, **$P < 0.01$, ***$P < 0.001$, ****$P < 0.0001$; ns, not significant.

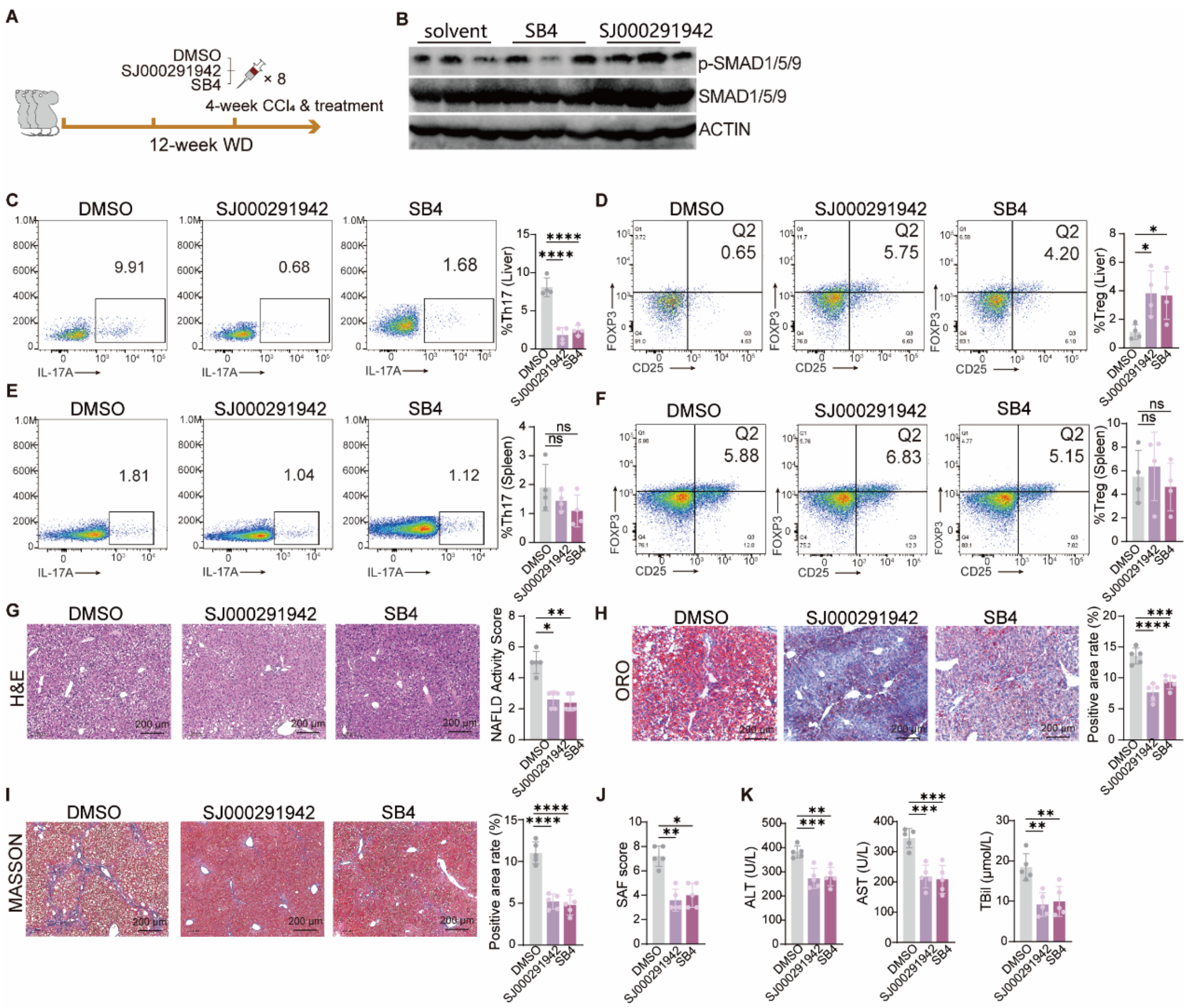


**Supplemental Figure 10. Amplification of BMP signaling confers therapeutic benefits in a mouse MASH model. A,** Schematic of the therapeutic experiment using two BMP pathway agonists: SB4 (a specific BMP4 signaling agonist) and SJ000291942 (a pan-BMP signaling agonist). **B,** Western blot detection of phosphorylated SMAD1/5/9 levels in livers treated with two BMP pathway agonists. The experiment was conducted one time. **C-F,** Flow cytometric analysis of the effects of the two agonists on the proportions of hepatic Th17 (**C**), hepatic Treg (**D**), splenic Th17 (**E**) and splenic Treg (**F**) (*n* = 4). **G–J,** H&E (**G**), Oil Red O (**H**), and Masson's trichrome (**I**) staining of liver sections from the three groups, followed by NAS (**G**) and SAF (**J**) scoring (*n* = 5) (scale bar: 200 μm). **K,** Serum liver function indices (ALT, AST, total

bilirubin) for the three groups ($n$ = 5). Data are mean ± s.d. Kruskal–Wallis *H*-test with Dunn's post hoc test for **G** and **J**; One-way ANOVA with Bonferroni post hoc analysis for other comparisons. *$P < 0.05$, ****$P < 0.01$**, *******$P < 0.001$, ********$P < 0.0001$; ns, not significant.

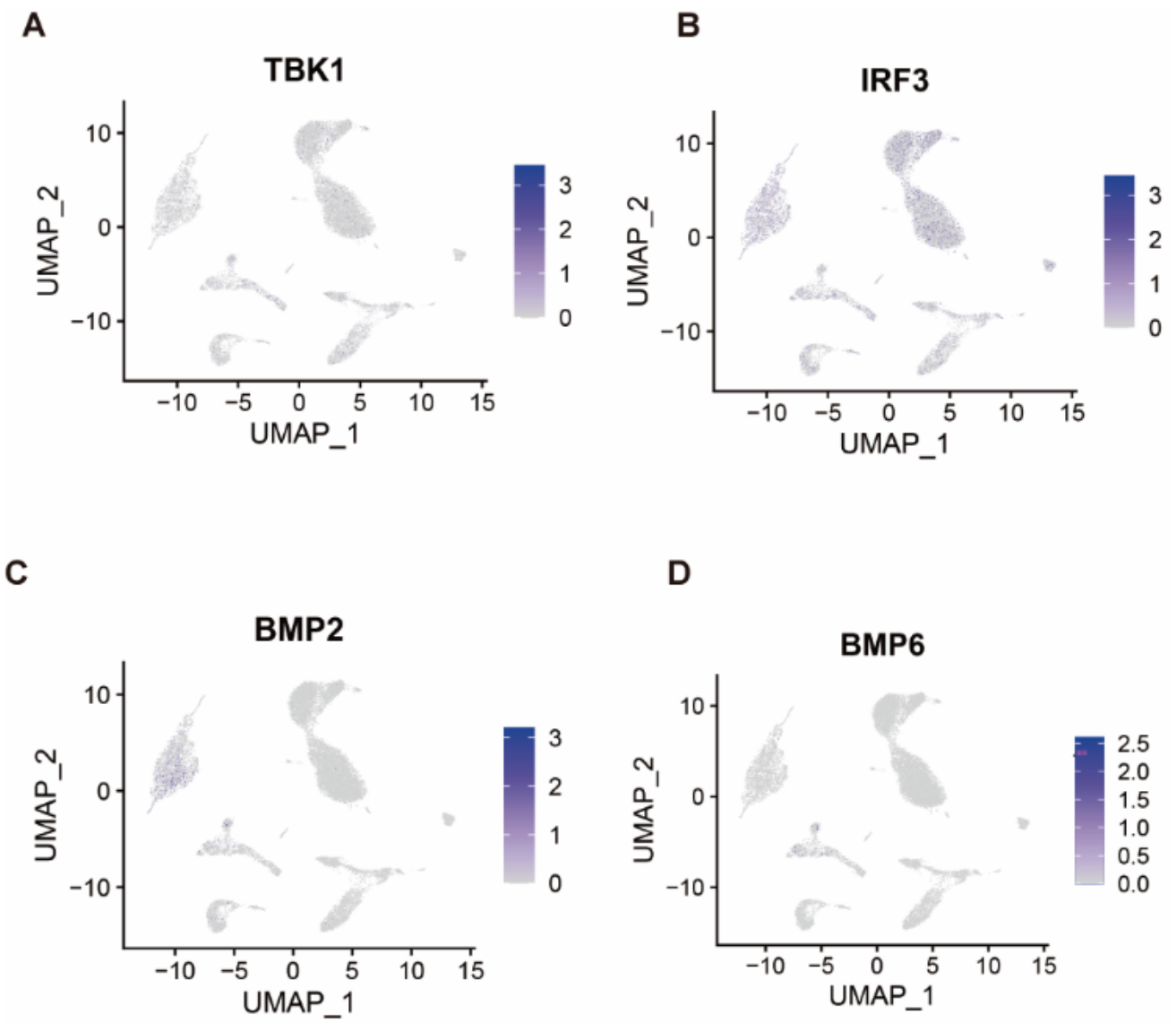


**Supplemental Figure 11. Gene expression in human MASH liver scRNA-seq data.**

**A–D,** UMAP plots showing the expression of *TBK1* (**A**), *IRF3* (**B**), *BMP2* (**C**), and *BMP4* (**D**) across all cell types (GSE136103).

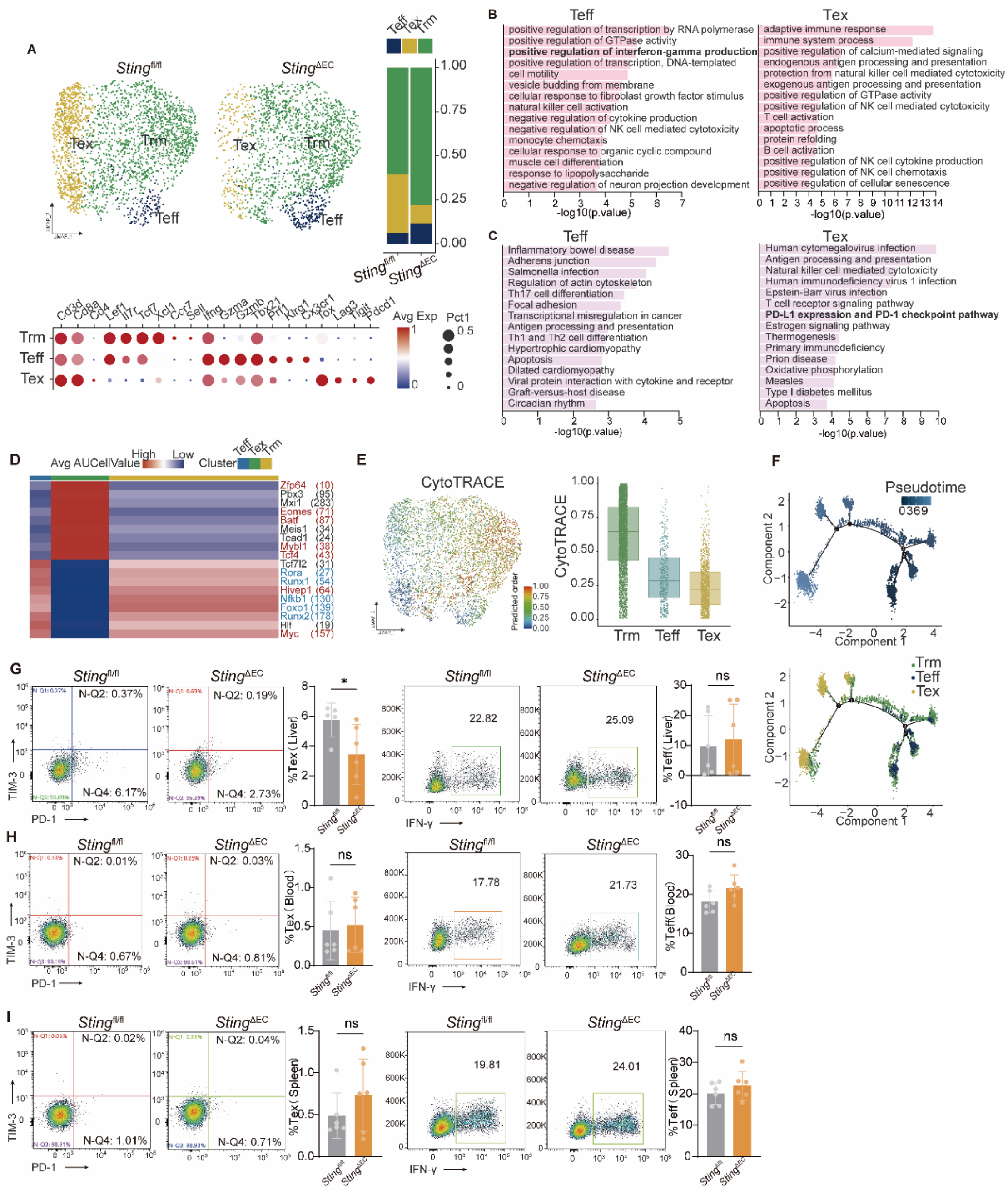


**Supplemental Figure 12. Endothelial *Sting* deletion alleviates CD8+ T cell exhaustion. A,** Types, proportions, and marker genes of CD8+ T cells in livers of *Sting*fl/fl and *Sting*ΔEC mice subjected to WD-$CCl_4$. **B, C,** GO-MF (**B**) and KEGG (**C**) pathway enrichment analysis of effector T cells (Teff) and exhausted T cells (Tex). **D,** SCENIC analysis of transcription factor regulatory activity across CD8+ T cell subsets. **E, F,** Differentiation trajectories of CD8+ T cell subsets inferred through CytoTRACE

(**E**) and pseudotime analysis (**F**). **G-I,** Flow cytometric measurement of Tex and Teff proportions in liver, spleen, and peripheral blood of WD-$CCl_4$-induced mice ($n$ = 5-6). Data are mean ± s.d. Two-tailed Student's $t$-test for all comparisons. *$P < 0.05$; ns, not significant.

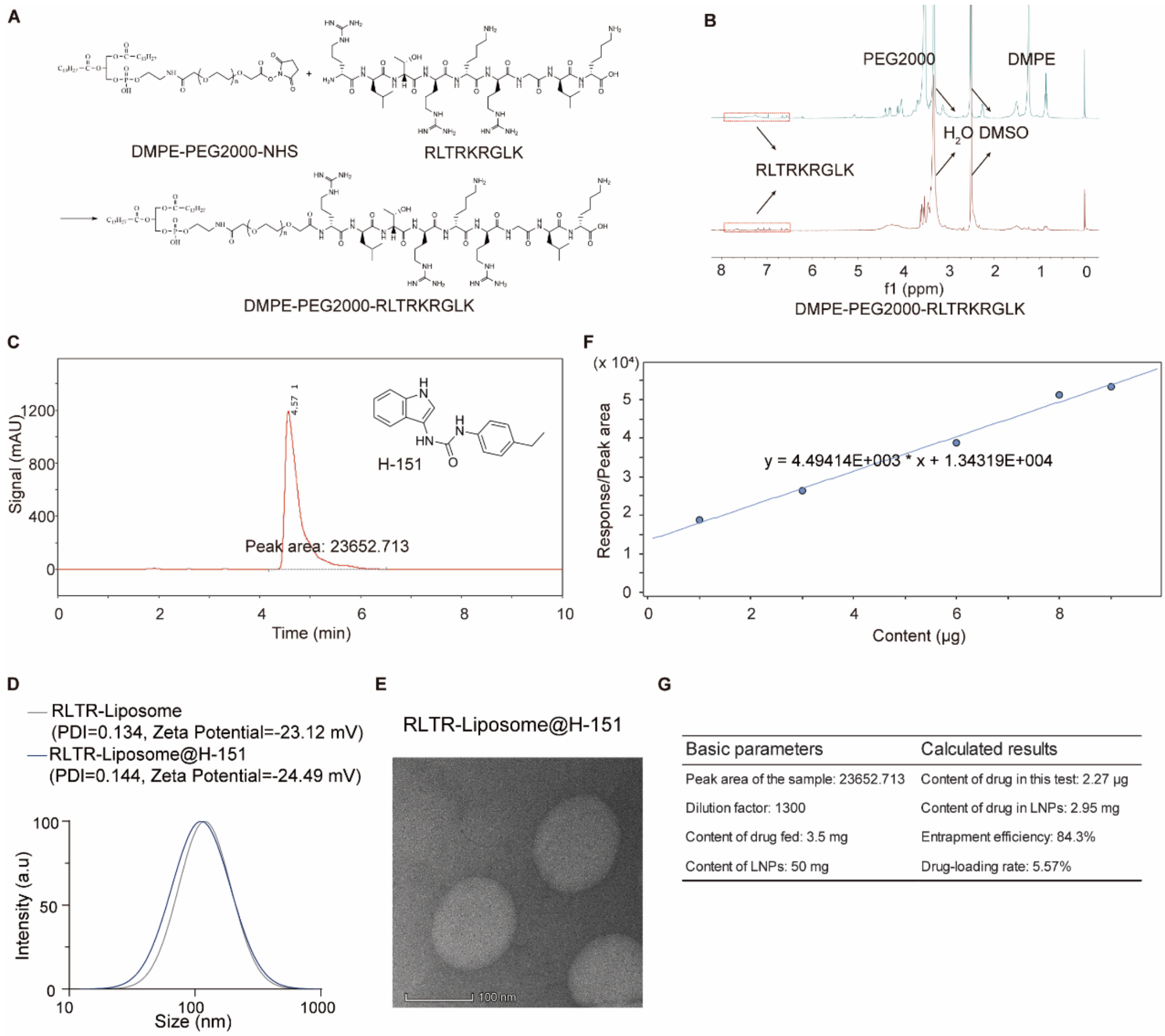


| Basic parameters | Calculated results |
|---|---|
| Peak area of the sample: 23652.713 | Content of drug in this test: 2.27 μg |
| Dilution factor: 1300 | Content of drug in LNPs: 2.95 mg |
| Content of drug fed: 3.5 mg | Entrapment efficiency: 84.3% |
| Content of LNPs: 50 mg | Drug-loading rate: 5.57% |

**Supplemental Figure 13. Synthesis and characterization of RLTR-Liposome@H-151 (RLH). A,** Synthesis scheme and chemical structure of DMPE-PEG2000-RLTRKRGLK. **B,** $^{1}$H-NMR spectrum of DMPE-PEG2000-RLTRKRGLK. **C,** Liquid chromatography (HPLC) chromatogram of H-151. **D,** Size distribution of the indicated nanoparticles measured by NanoBrook 90plus PALS. **E,** Transmission electron microscopy (TEM) image of RLH (scale bar: 100 nm). **F,** Standard curve for determining RLH drug loading efficiency. **G,** Calculation of RLH drug-loading rate and encapsulation efficiency. The experiments for **B**-**F** were each repeated three times.

## Supplemental methods

**Histological staining and immunostaining.** Liver tissues were fixed in 10% formaldehyde for 48 hours. Paraffin-embedded liver sections were subjected to Hematoxylin and Eosin (H&E) staining (Hematoxylin, G1004, Servicebio, Wuhan, China; Eosin, BA-4024, Baso, Zhuhai, China) for histological examination. To evaluate hepatic steatosis, Oil Red O staining (O0625, Sigma-Aldrich, St. Louis, MO, USA) was performed on OCT-embedded liver sections. Liver fibrosis was assessed using Masson staining (G1006, Servicebio, Wuhan, China). All staining procedures were carried out following the manufacturer's protocols. Positive area rate of the image was qualified by Image J (1.53a). For immunohistochemistry staining, paraffin-embedded liver sections were incubated with primary antibodies at 4°C overnight. Subsequently, the sections were treated with a Two-step Detection Kit (PV-9001, ZSGB-BIO, Beijing, China), and positive signals were visualized using DAB staining (ZLI-9018, Zhongshan Biotech, Beijing, China). Histological and immunohistochemical images were captured with a light microscope (ECLIPSE 80i, Nikon, Tokyo, Japan). For immunofluorescence staining, either paraffin- or OCT-embedded liver sections were first incubated with primary antibodies at 4°C overnight, followed by incubation with fluorophore-conjugated secondary antibodies. Images were acquired using a fluorescence microscope (Olympus, Tokyo, Japan) and a confocal laser scanning microscope (TCS SP8, Leica, Wetzlar, Germany) in accordance with the manufacturer’s instructions. All antibodies used in this study are provided in Supplemental Table 2.

**Western blotting analysis**. For western blotting analysis, tissues and cells were homogenized in RIPA lysis buffer (P0013E; Beyotime Biotechnology, Shanghai, China) supplemented with protease (04693132001; Roche, Basel, Switzerland) and phosphatase inhibitor cocktails (4906837001; Roche). Total protein concentration was measured using a BCA Protein Assay Kit (23225; Thermo Fisher Scientific, Waltham, MA, USA). Equal amounts of protein were separated on 8-12% SDS-PAGE gels and subsequently transferred to PVDF membranes (IPVH00010; Millipore, Billerica, MA, USA). After blocking with 5% skim milk in TBST for 1 hour at room temperature, the membranes were incubated overnight at 4 °C with specific primary antibodies, followed by incubation with HRP-conjugated secondary antibodies for 1 hour at room temperature. Protein bands were visualized using an ECL kit (170-5061; Bio-Rad, Hercules, CA, USA) on a ChemiDoc MP Imaging System (Bio-Rad). GAPDH or ACTIN served as the loading control. Relative quantification of protein expression was conducted via Image Lab Software (v6.1). Details of the antibodies used are provided in Supplemental Table 2.

**mRNA isolation and qPCR assay**. The total mRNA was isolated from cells and tissues using TRIzol reagent (T9424; Sigma-Aldrich, St. Louis, MO, USA), and its concentration was quantified with a Nanodrop 2000 spectrophotometer (Thermo Fisher). Subsequently, RNA was reverse transcribed into cDNA using HiScript II Q RT SuperMix for qPCR (R223-01, Vazyme, Nanjing, China), which includes a gDNA wiper, according to the manufacturer’s protocol. mRNA expression levels were

quantified with ChamQ SYBR qPCR Master Mix (Q311-02, Vazyme) on a LightCycler 480 Instrument II real-time PCR system (Roche, Basel, Switzerland), following the supplier's instructions. The mRNA expression of target genes was normalized to *Actb*. To measure cytosolic mtDNA content, PA-stimulated primary LSECs were collected and divided into two equal portions for cytosolic and total DNA isolation using the Cell Fractionation Kit (ab109719; Abcam), according to the manufacturer's instructions. Cytosolic and whole-cell DNA were subsequently extracted with QIAquick Nucleotide Removal Columns (28115; Qiagen) and quantified by qPCR. All primers used are listed in Supplemental Table 3.

**Bulk RNA sequencing**. Total RNA was extracted from the liver tissues of *Sting1*$^{flox/flox}$ and *Sting1*$^{flox/flox}$*Cdh5*$^{Cre}$ mice following 10-week CDAHFD administration (n=3 per group), using TRIzol reagent (T9424; Sigma-Aldrich; St. Louis, MO, USA) as indicated above. The sequencing as well as bioinformatic analysis, including differentially expressed genes (DEGs) visualization in a volcano plot and Gene Set Enrichment Analysis (GSEA) on pathways from KEGG and Reactome databases, was performed by Gene Denovo Corporation (Guangzhou, China), using Omicsmart, a dynamic real-time interactive online platform for data analysis (http://www.omicsmart.com).

**Single-cell RNA sequencing and analysis.** Single cells were isolated from the liver tissues of *Sting1*$^{flox/flox}$ and *Sting1*$^{flox/flox}$*Cdh5*$^{Cre}$ mice following 12-week WD-$CCL_4$

administration (n=3 per group). scRNA-seq data analysis was performed by NovelBio Co., Ltd. using the NovelBrain Cloud Analysis Platform (www.novelbrain.com). Raw sequencing data were filtered using fastp with default parameters to remove adapter sequences and low-quality reads, yielding clean data. Feature-barcode matrices were generated by aligning reads to the mouse reference genome (mm10, Ensembl, version 100) using CellRanger v7.1.0. Cell quality control retained cells expressing >300 genes with a mitochondrial UMI rate <20%. Mitochondrial genes were excluded from the expression matrix. The Seurat package (v4.1.1; https://satijalab.org/seurat/) was used for normalization and regression of UMI counts and mitochondrial gene percentage, generating scaled expression data. Principal component analysis (PCA) was performed on the top 2,000 highly variable genes, and the first 10 principal components were used for t-SNE and UMAP visualization. Unsupervised cell clustering was conducted via graph-based clustering using the top 10 PCA components. Marker genes were identified using the FindAllMarkers function with the Wilcoxon rank-sum test (criteria: log2FC > 0.25, p-value < 0.05, min.pct1 > 0.1). Clusters corresponding to the same cell type were re-clustered for fine-grained annotation, followed by re-clustering and marker gene analysis. Differentially expressed genes (DEGs) between samples were identified using the FindMarkers function with the Wilcoxon rank-sum test (criteria: log2FC > 0.25, p-value < 0.05, min.pct1 > 0.1). Gene Ontology (GO) enrichment analysis was performed using annotations from NCBI (http://www.ncbi.nlm.nih.gov/), the Gene Ontology database (http://www.geneontology.org/), and UniProt (http://www.uniprot.org/). Significant GO terms were determined via Fisher’s exact test with false discovery rate

(FDR) correction for multiple comparisons. Pathway analysis was conducted using the Kyoto Encyclopedia of Genes and Genomes (KEGG) database, with significance assessed by Fisher's exact test and FDR correction. Cell-cell communication was analyzed using CellPhoneD, a public repository of ligands, receptors, and interactions. Membrane, secreted, and peripheral proteins were annotated for clusters across time points, and significant interactions were identified based on normalized expression data (p-value $< 0.05$). Transcription factor regulatory networks were inferred using pySCENIC (v0.9.5) with the 20,000-motif database for RcisTarget and GRNboost. Gene set enrichment analysis was performed using Fisher's exact test with Benjamini-Hochberg FDR correction. Annotated gene sets included KEGG (201900613), Hallmark (h.all.7.0; https://www.gsea-msigdb.org/gsea/msigdb/index.jsp), chromosome location gene sets (https://ftp.ncbi.nlm.nih.gov/gene/DATA/GENE_INFO/Mammalia/), and a custom 41-gene signature (immune-, cytokine-, and neurobiology-related terms from CellPhoneDB). QUSAGE (v2.16.1) was used to quantify relative gene set activation, and GSVA software package was applied to calculate gene set scores per cell. Violin plots were generated using R package, and score differences between cell types were tested via the Wilcoxon rank-sum test. We applied Single-Cell Trajectories analysis using Monocle2 (http://cole-trapnell-lab.github.io/monocle-release) with the DDR-Tree algorithm and default parameters. Prior to Monocle analysis, we selected marker genes from the Seurat clustering results and raw expression counts of cells that passed filtering. Based on pseudo-time analysis, branch expression analysis modeling (BEAM

Analysis) was performed to identify genes determining branch fates. CytoTRACE analysis was applied to predict differentiation states with default parameters.

**Flow Cytometry.** Sample Preparation: Mouse spleen samples were mechanically dissociated, filtered, and centrifuged at 350 × g for 6 min. Red blood cells were lysed for 10 min, and splenocytes were collected. Mouse liver samples were perfused with saline under deep anesthesia, excised, minced, and digested in digestion buffer containing collagenase and DNase I at 37°C for 30 min. Digestion was stopped with PBS containing 2% FBS. Cell suspensions were filtered through a 70 μm strainer, followed by differential centrifugation and 33% Percoll gradient centrifugation (800 × g, 25 min). Red blood cells were lysed on ice for 10 min, and hepatic lymphocytes were collected. Peripheral blood mononuclear cells (PBMCs) were isolated using density gradient centrifugation at 500 × g for 30 min at room temperature.

Intracellular Cytokine Staining: Cells were counted and adjusted to 1–2 × 10^7 cells/ml. Cells were stimulated with cell stimulation cocktail for 4–6 h at 37°C, then washed with PBS. Zombie NIR dye was used for viability staining (10 min, on ice). Fc receptors were blocked with anti-mouse CD16/32 antibody for 10 min on ice. Surface marker staining was performed using fluorophore-conjugated antibodies for 15 min on ice in the dark. Cells were fixed with fixation buffer for 20 min at room temperature, washed, and permeabilized using perm wash buffer. Intracellular cytokines were stained for 20 min at room temperature in the dark. Cells were washed and resuspended in Cell

Staining Buffer for flow cytometric analysis.

Nuclear Transcription Factor Staining: After surface staining, cells were fixed using True-Nuclear™ Fix Buffer for 60 min at room temperature in the dark. Cells were washed twice with True-Nuclear™ Perm Buffer and incubated with transcription factor antibodies in Perm Buffer (30 min, room temperature, dark). Cells were washed and resuspended in Cell Staining Buffer prior to flow cytometric acquisition. All anti-body used are listed in Supplemental Table 4. Gating strategies are provided at the end of the Supplemental Materials.

**Enzyme-linked immunosorbent assay (ELISA).** The hepatic BMP4 protein level of *Sting*$^{fl/fl}$ and *Sting*$^{\Delta EC}$ mice after 12 weeks of WD-$CCL_4$ treatment were quantified using ELISA with specific kits (JL12369; Jonlnbio, Shanghai, China).

**Biochemical analysis.** The serum concentrations of alanine aminotransferase (ALT), aspartate aminotransferase (AST), serum glucose, and total bilirubin (TBil) were measured by an ADVIA 2400 Chemistry System Analyzer (Siemens, Tarrytown, NY, USA), according to the manufacturer's instructions.

**Plasmid construction and transfection.** Expression plasmids for EBF1 (P8356), THRB (P33162), PBX1 (P42995), NFKB1/p105 (P56017), RELA/p65 (P45239 and P40120), FOS (P87126), and JUN(P60132) were purchased from Miaolingbio (Wuhan,

China). The HEY1 expression plasmid was constructed by inserting the full-length core coding sequence into the pcDNA5 vector using PCR-based cloning. Plasmids were transfected into cells using Lipofectamine$^{TM}$ 3000, following the manufacturer's protocol. Cells were harvested 24 hours post-transfection for subsequent experiments.

**Public Database analysis.** To analyze human LSECs in MASLD livers, we examined a published single-nucleus RNA sequencing (snRNA-seq) dataset (GSE202379, including all samples) and a single-cell RNA sequencing (scRNA-seq) dataset (GSE136103, including all control and MASH samples). Data processing was performed using R (v4.3.1) and Seurat (v5.3.0). Following quality control, datasets were normalized using SCTransform and integrated with Harmony to correct batch effects. Dimensionality reduction was performed using PCA, and cell clusters were visualized via UMAP. LSECs were subsetted based on endothelial cell annotations, re-clustered at a resolution of 0.2 using the first 20 principal components, and classified into functional states (homeostatic, inflammatory, capillarized, profibrotic) using AddModuleScore based on established gene signatures. For pathway-level analysis, gene sets including innate immunity (NOD-like, RIG-I-like, Toll-like, cytosolic DNA sensing), lipid metabolism (peroxisomal lipid metabolism, PPAR signaling, fatty acid metabolism), and damage/stress response pathways (apoptosis, p53 signaling, DNA repair, cellular stress response) from msigdbr (v25.1.1) were analyzed. Cells were aggregated by patient to generate pseudo-bulk pathway activity matrices, with mean scores per pathway category. Category scores were discretized into tertiles (Low,

Medium, High) for visualization. Alluvial plots (ggalluvial v0.12.5) depicted combinatorial activity patterns across the three categories, while Spearman correlations between lipid metabolism and innate immunity pathways were calculated, adjusted using the Benjamini-Hochberg method, and visualized with side-by-side heatmaps (pheatmap v1.0.13).

For functional analysis, differential expression between LSECs subpopulations was assessed using the Wilcoxon rank-sum test. Gene Set Variation Analysis (GSVA) was performed using the GSVA (v1.50.5) to evaluate pathway activity scores at the single-cell level. Additionally, Gene Set Enrichment Analysis (GSEA) on MSigDB Hallmark and KEGG gene sets was conducted using fgsea (v1.28.0). Cell-cell communication analysis was performed using CellChat (v1.6.1) to infer ligand-receptor interactions between LSEC subpopulations and other hepatic cell types. All visualizations were generated using ggplot2 (v3.5.2) and ggsci (v4.1.0). Statistical significance was defined as adjusted $P < 0.05$ for all analyses.

The mRNA expression levels of components within the cGAS-STING signaling pathway in human liver were obtained from The Human Protein Atlas (www.proteinatlas.org) and DISCO (disco.bii.a-star.edu.sg). Hepatic mRNA expression of *BMP4*, *ACTA2*, and *COL1A1* in patients with MASLD and controls was analyzed using GEO2R based on two datasets (GSE135251 and GSE246221), and the correlation analysis was conducted after the exclusion of outliers. Serum BMP4 protein

levels in MASLD patients and controls were derived from the proteomics data (1).

## References


1. Govaere O, et al. A proteo-transcriptomic map of non-alcoholic fatty liver disease signatures. *Nat Metab.* 2023;5(4):572-8.

## Supplemental tables

**Supplemental table 1. Primers for genotype**

| Primer | Primer Sequence (5'-3') |
|---|---|
| P1 | TATGAAGGCTTGGGATGA |
| P2 | CTGGAAGGCAGATGAAAA |
| P3 | TCGCCTTCTTGACGAGTTCT |
| P4 | CCAGGCTGACCAAGCTGAG |
| P5 | CCTGGCGATCCCTGAACA |
| P6 | AGTGGCCTCTTCCAGAAATG |
| P7 | TGCGACTGTGTCTGATTTCC |

**Supplemental table 2. Antibodies for western blotting, immunofluorescent staining and immunoprecipitation**

| Antibody (dilution/amount) | Source | Identifier |
|---|---|---|
| Anti-cGAS (IF, 1: 200) | Proteintech | 29958-1-AP |
| Anti-STING (WB, 1: 1000) | Cell Signaling Technology | 13647 |
| Anti-STING (IF, 1: 200) | Cell Signaling Technology | 90947 |
| Anti-TBK1 (IF, 1: 200) | Abcam | Ab40676 |
| Anti-IRF3 (IF, 1: 200; WB, 1: 500) | Proteintech | 80519-1-RR |
| Anti-p-IRF3 (IF, 1: 200; WB, 1: 500) | Cell Signaling Technology | 29047 |
| Anti-p65 (WB, 1: 500) | Cell Signaling Technology | 8242 |
| Anti-p-p65 (WB, 1: 500) | Cell Signaling Technology | 3033 |
| Anti-CD31 (IF, 1: 200) | R&D | AF3628 |
| Anti-LYVE1 (IF, 1: 200) | Thermo Fischer | 14-0443-80 |
| Anti-SMAD1/5/9 (WB, 1: 1000) | Abclonal | A23208 |
| Anti-p-SMAD1/5/9 (WB, 1: 1000) | Cell Signaling Technology | 13820 |
| Anti-HEY1 (WB, 1: 1000) | Abclonal | A16110 |
| Anti-Flag (Mouse mAb) (IP, 2 μg/sample) | Medical & Biological Laboratories | M185 |
| Anti-Flag (Rabbit pAb) (WB, 1: 1000) | Cell Signaling Technology | 14793 |
| Anti-β-Actin (WB, 1: 1000) | Abclonal | AC026 |
| Anti-GAPDH (WB, 1: 1000) | Cell Signaling Technology | 5174 |

**Supplemental table 3. Primers for quantitative PCR.**

| Gene | Forward (5'-3') | Reverse (5'-3') |
|---|---|---|
| *Actb* | GTGACGTTGACATCCGTAAAGA | GCCGGACTCATCGTACTCC |
| *Cgas* | ACGAGGAAATCCGCTGAGTC | TCCACACTGACATCTATATCTTTGA |
| *Sting* | GTTGATCTTACCAGGGCTCC | ATGTACAGTCTTCGGCTCCC |
| *Tbk1* | TGCCGTTTAGACCCTTCGAG | GTGCCTGAAGACCCTGAGAA |
| *Irf3* | ACAAAGAAGGGGGTTGCGTG | TTCCATGCTCTAGCCAGGGG |
| *Bmp4* | TCCGTCCCTGATGGGATTCT | TGGTGTCTCATTGGTTCCTGC |
| *BMP4* | CGGAAGCTAGGTGAGTGTGG | TCGAGATAGCTTGGACGGGA |
| *ACTB* | TGTGGATCAGCAAGCAGGAG | GTCCTCGGCCACATTGTGAA |
| *Mt-Nd1* | CAAACACTTATTACAACCCAAGAACA | TCATATTATGGCTATGGGTCAGG |
| *Mt-Nd2* | CCATCAACTCAATCTCACTTCTATG | GAATCCTGTTAGTGGTGGAAGG |
| *BMP4* (promoter) | GCAGAGGCTGTGATTTACTT | GTGGAATGTGGTGGCTTTTA |

**Supplemental table 4. Antibodies for flow cytometry.**

| Antibody | Source | Identifier |
|---|---|---|
| PerCP anti-mouse CD45 Antibody | Biolegend | 103129 |
| BV510 anti-mouse CD3 Antibody | Biolegend | 100234 |
| FITC anti-mouse CD4 Antibody | Biolegend | 100405 |
| AF647anti-mouse FOXP3 Antibody | Biolegend | 126407 |
| BV421anti-mouse IL-17A Antibody | Biolegend | 506926 |
| PE anti-mouse CD25 Antibody | Biolegend | 102007 |
| BV605 anti-mouse CD8a Antibody | Biolegend | 100744 |
| PE anti-mouse IFN-γ Antibody | Biolegend | 505807 |
| PE anti-mouse CD62L Antibody | Biolegend | 104407 |
| APC anti-mouse CD44 Antibody | Biolegend | 103011 |
| anti-mouse CD16/32 Antibody | Biolegend | 101319 |
| Zombie NIR™ Fixable Viability Kit | Biolegend | 423105 |
| PE-Cy7 anti-mouse Tim3 Antibody | Biolegend | 119716 |
| APC anti-mouse PD1 Antibody | Biolegend | 135209 |

# Th17 gating strategy

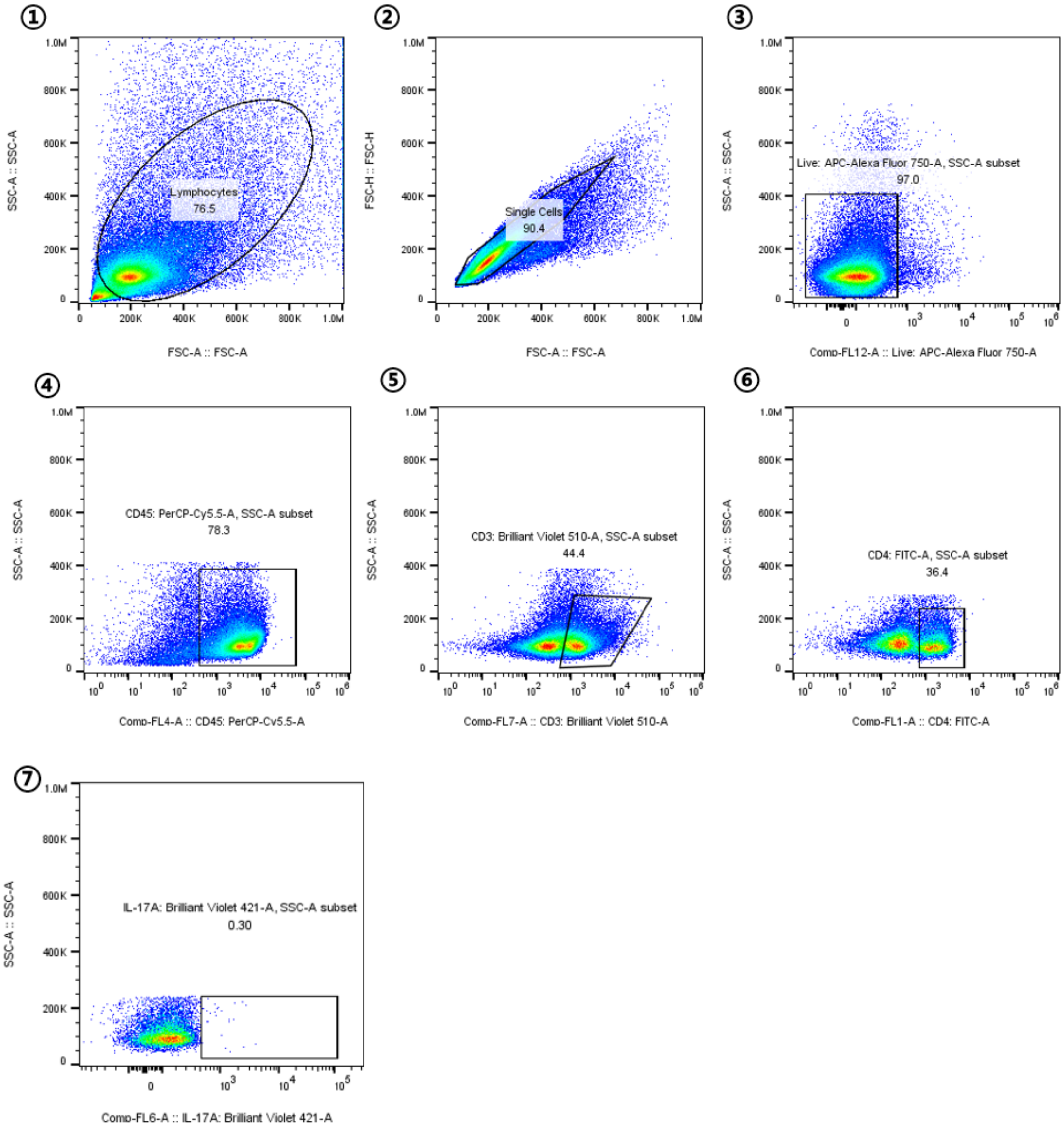

## Treg gating strategy

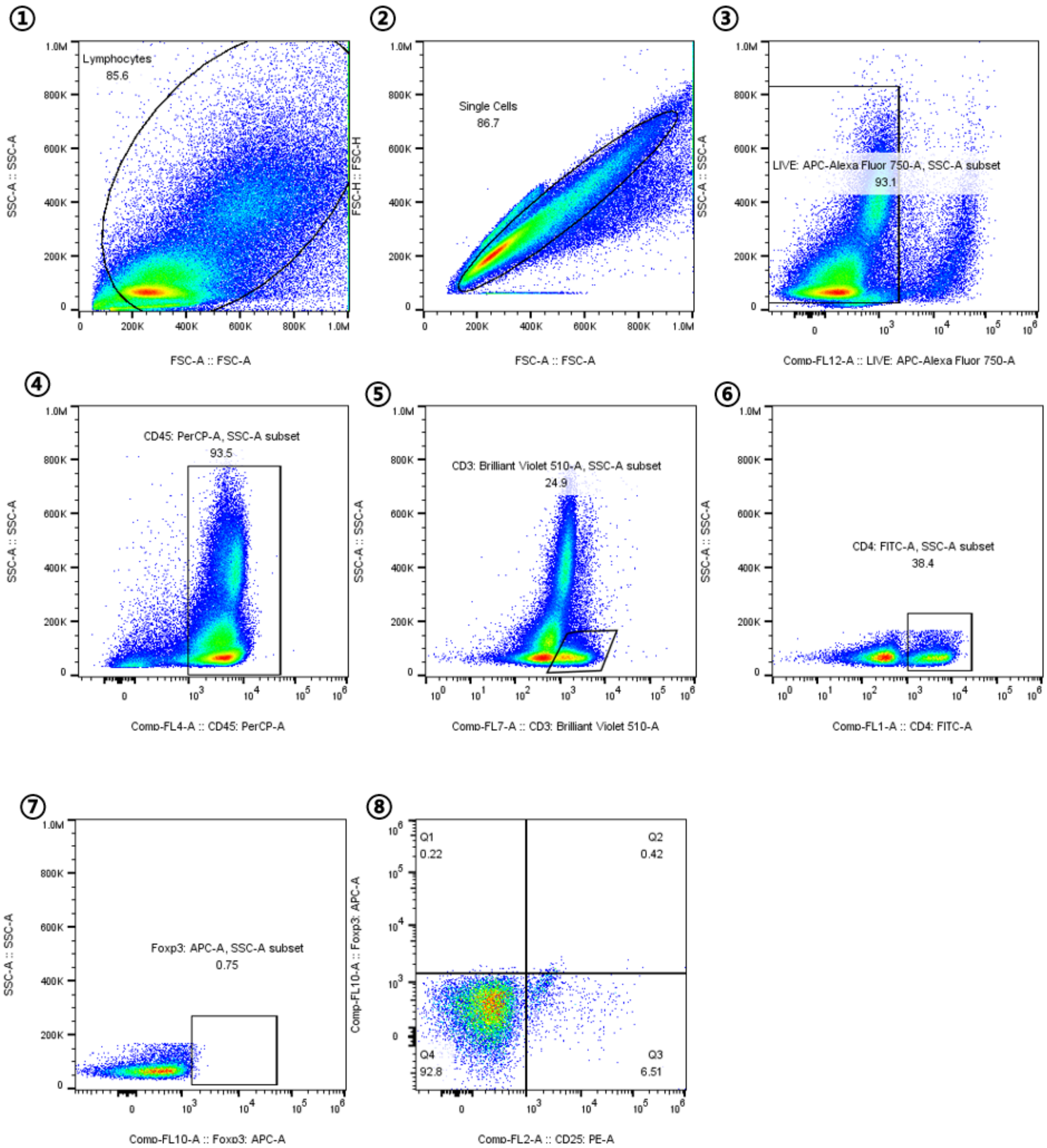

# Teff and Tex gating strategy

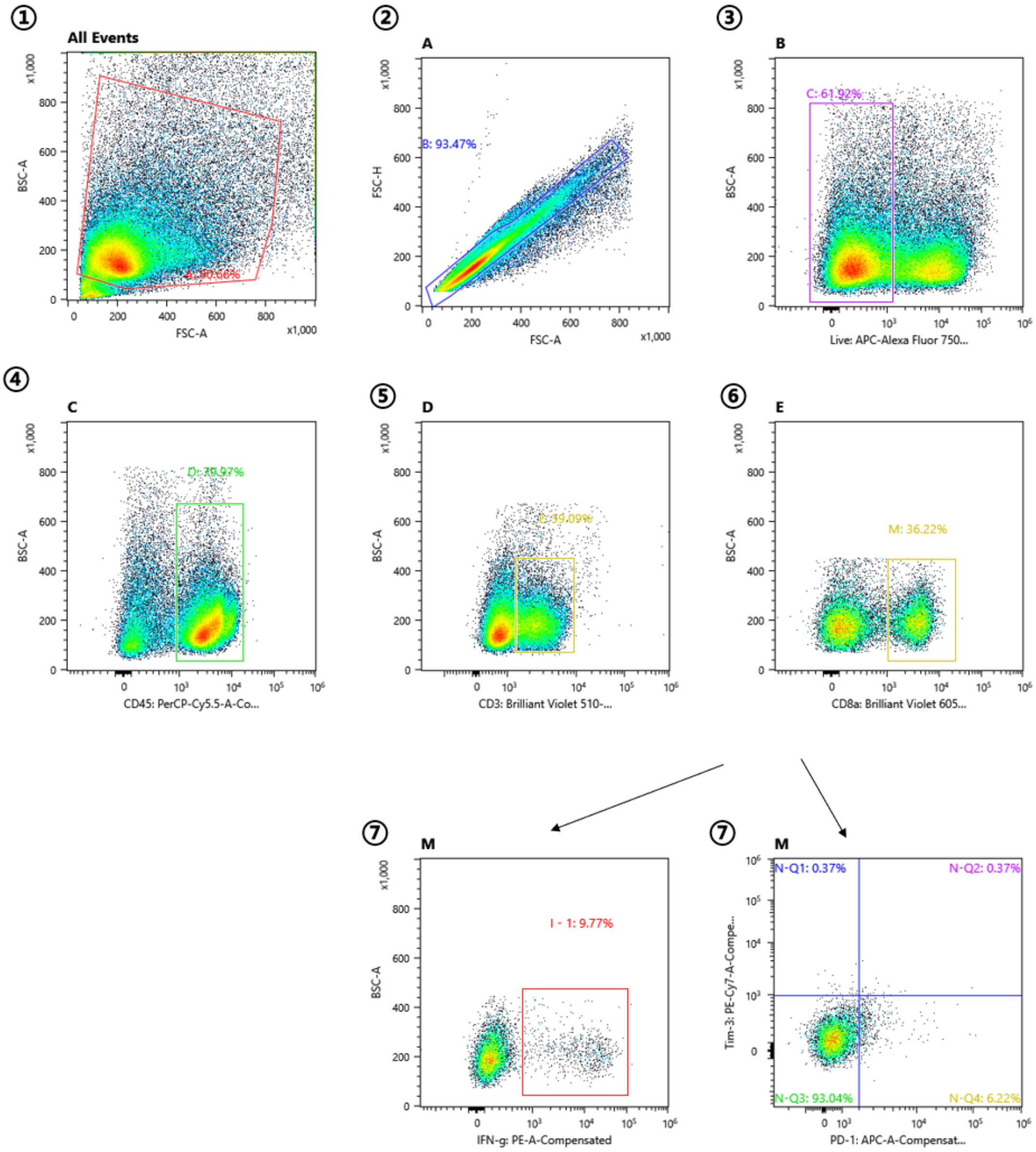

Supplement: Supplementary file 1 [file jci-136-207031-s129.pdf]
