# Supplementary material for: Metabolic stress–activated endothelial STING rewires the sinusoidal immune niche to promote MASLD progression
Source: J Clin Invest. 2026 Aug 6;136(19):e207031. doi: 10.1172/JCI207031 (PMC13626822; doi:10.1172/JCI207031)

Fig. 2D

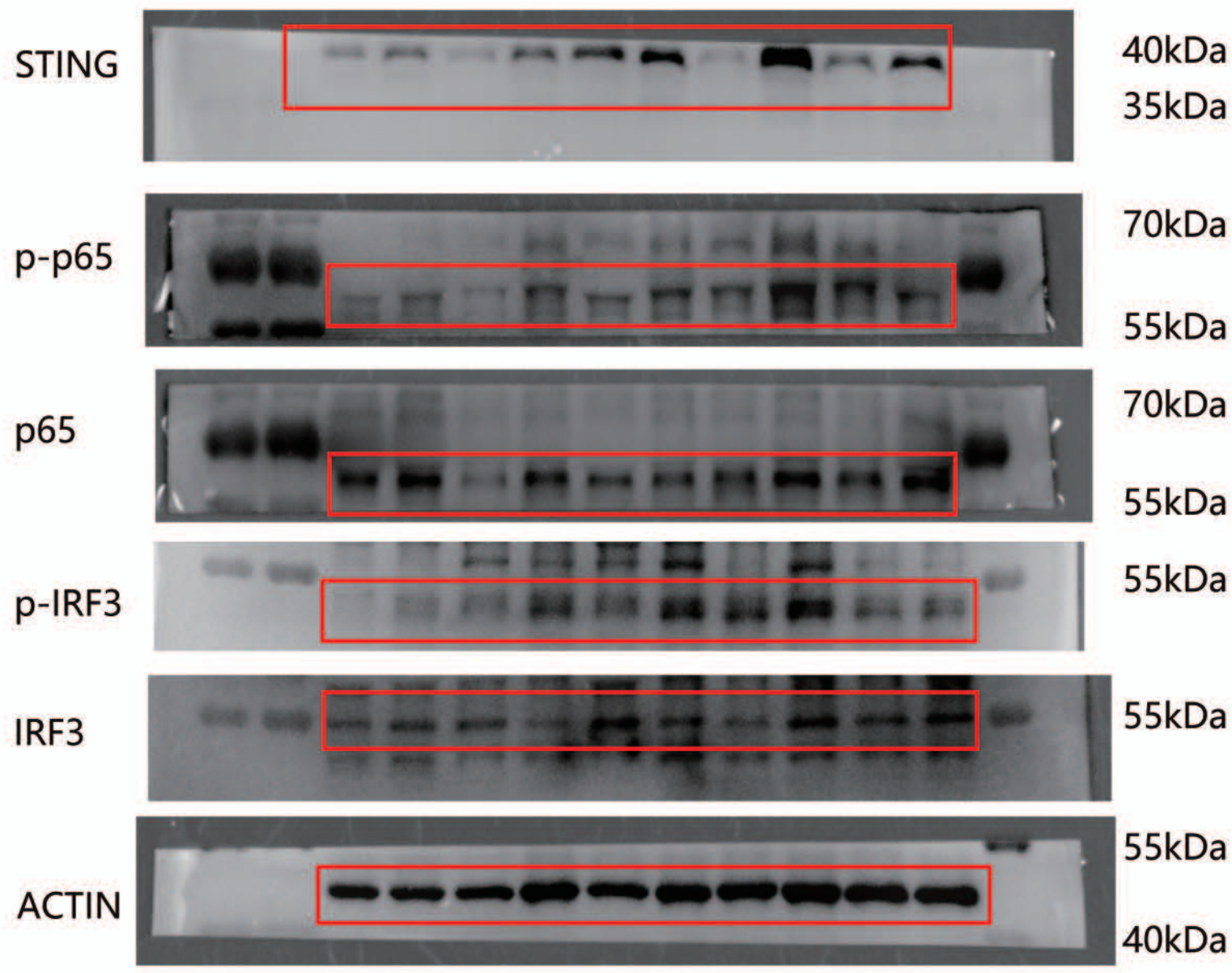

Fig. 8E

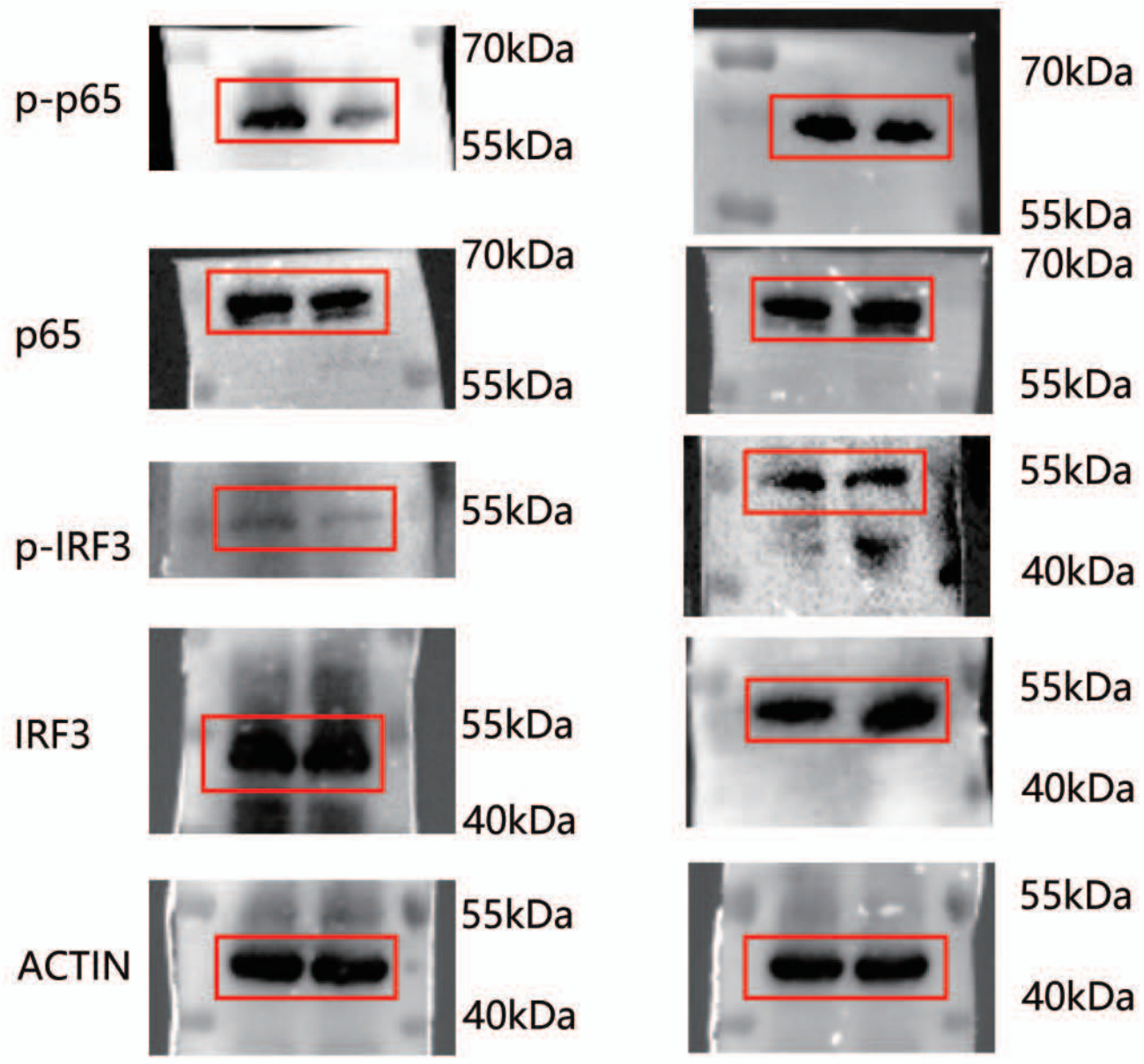

Fig. S1D

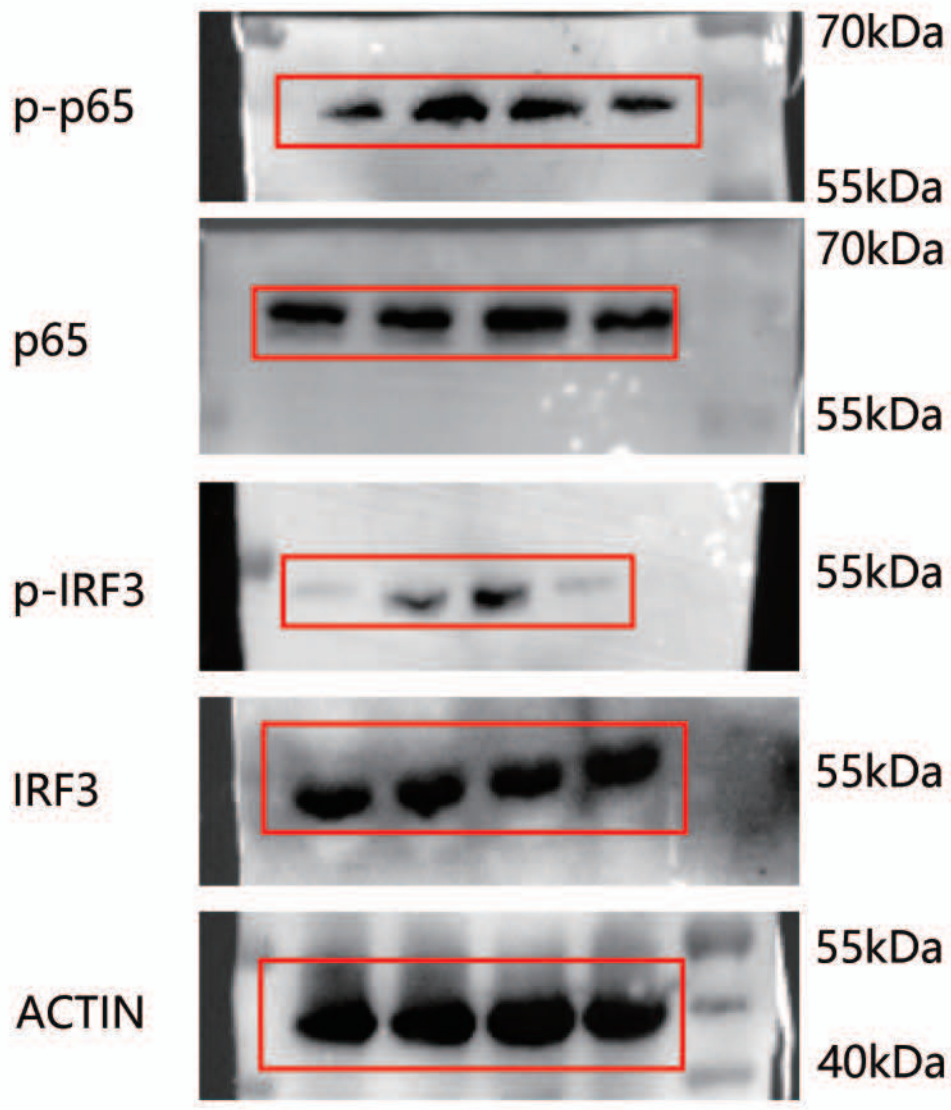

Fig. S2C

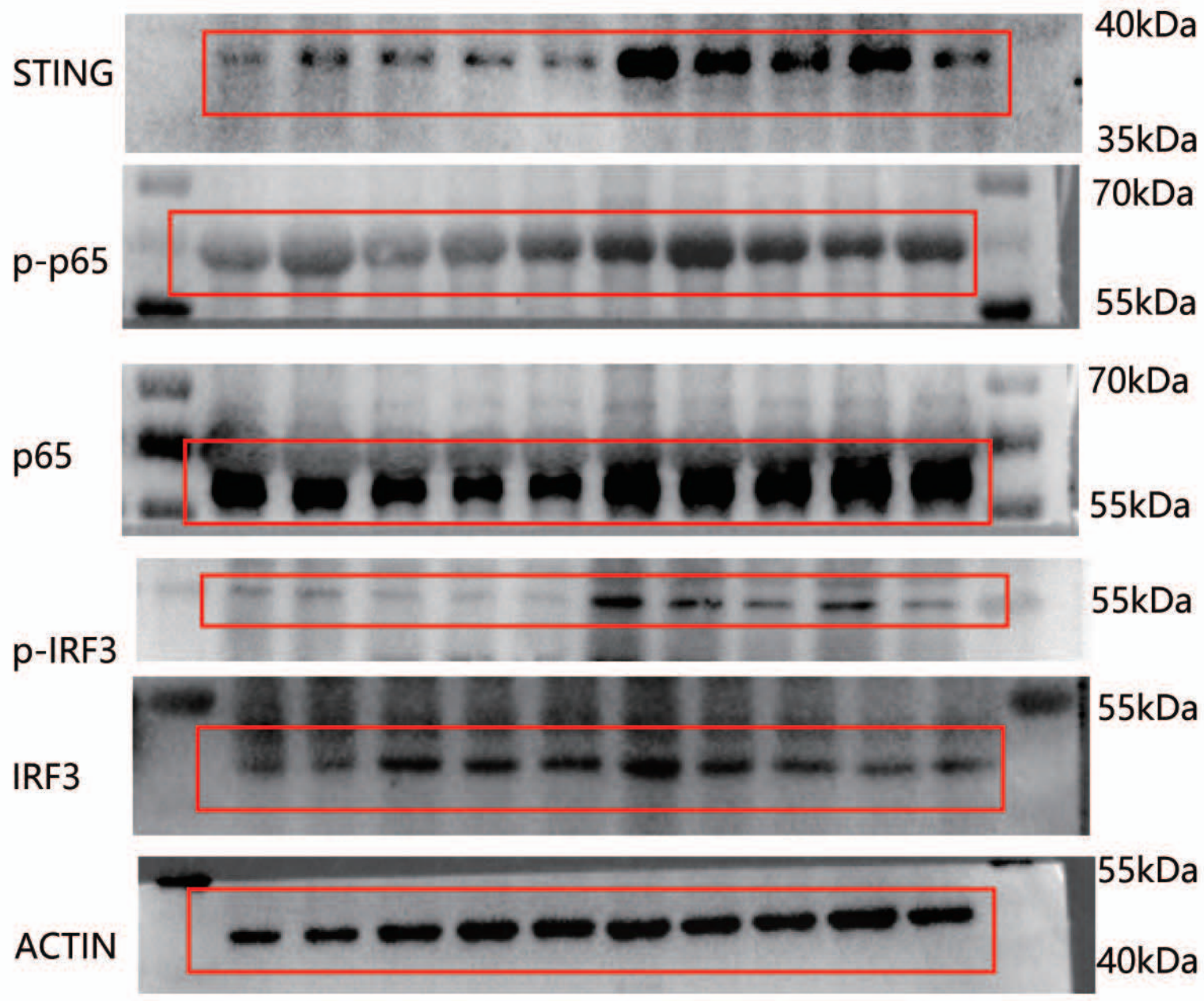

Fig. S3B

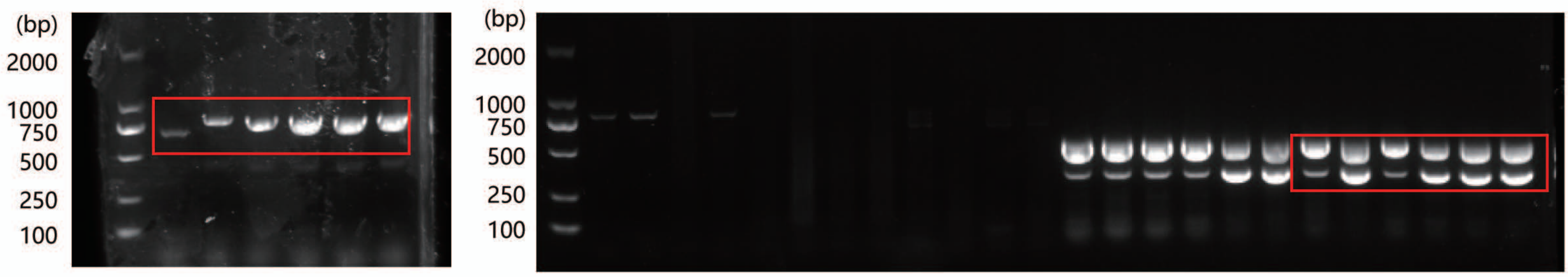

Fig. S3D

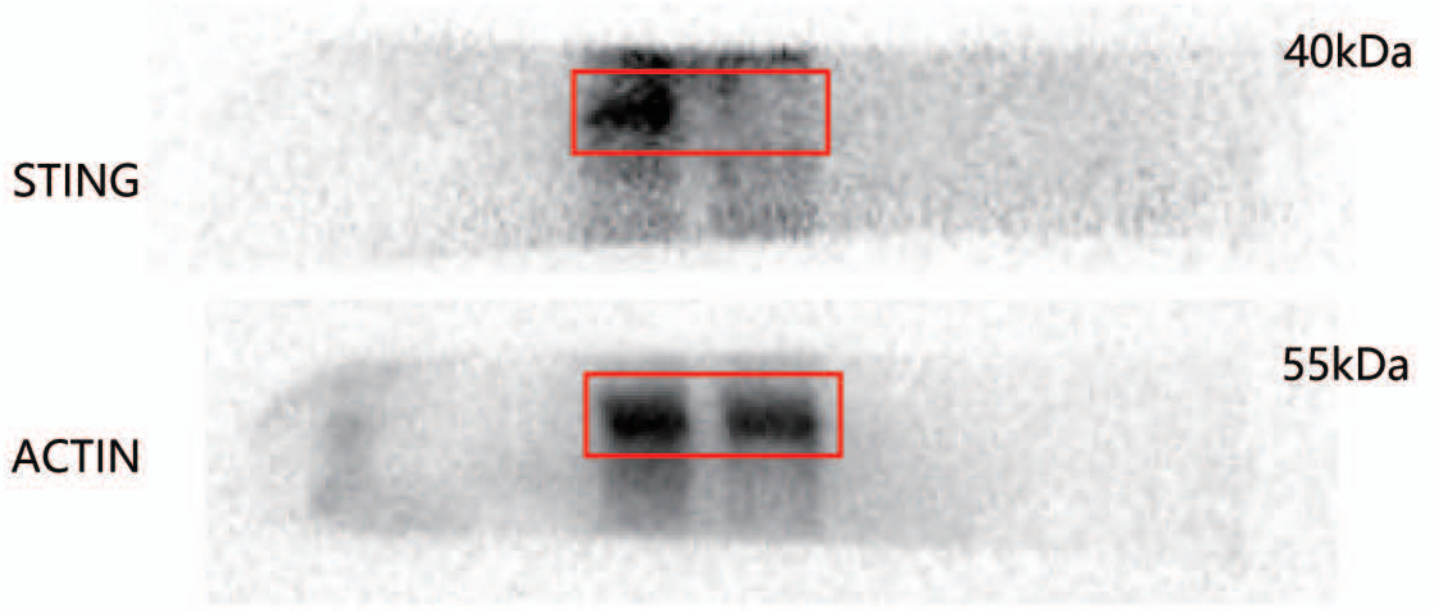

Fig. S5A

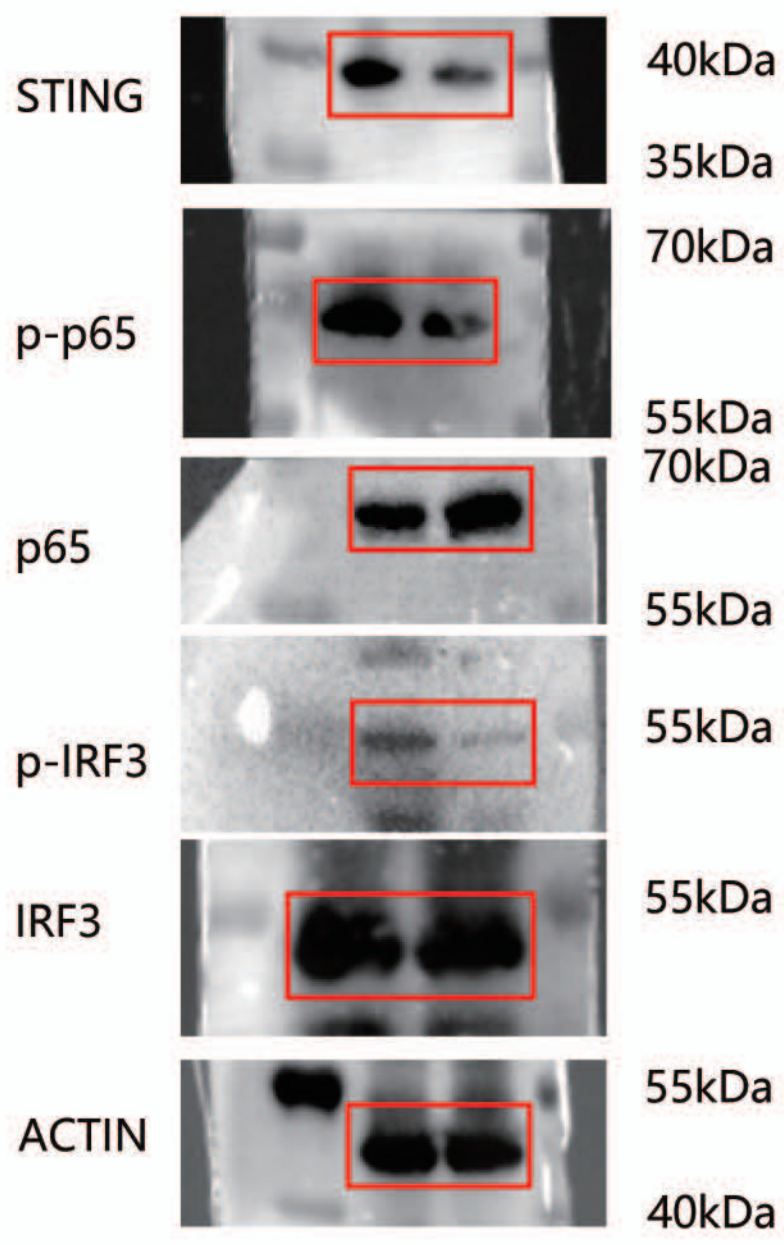

Fig. S8C

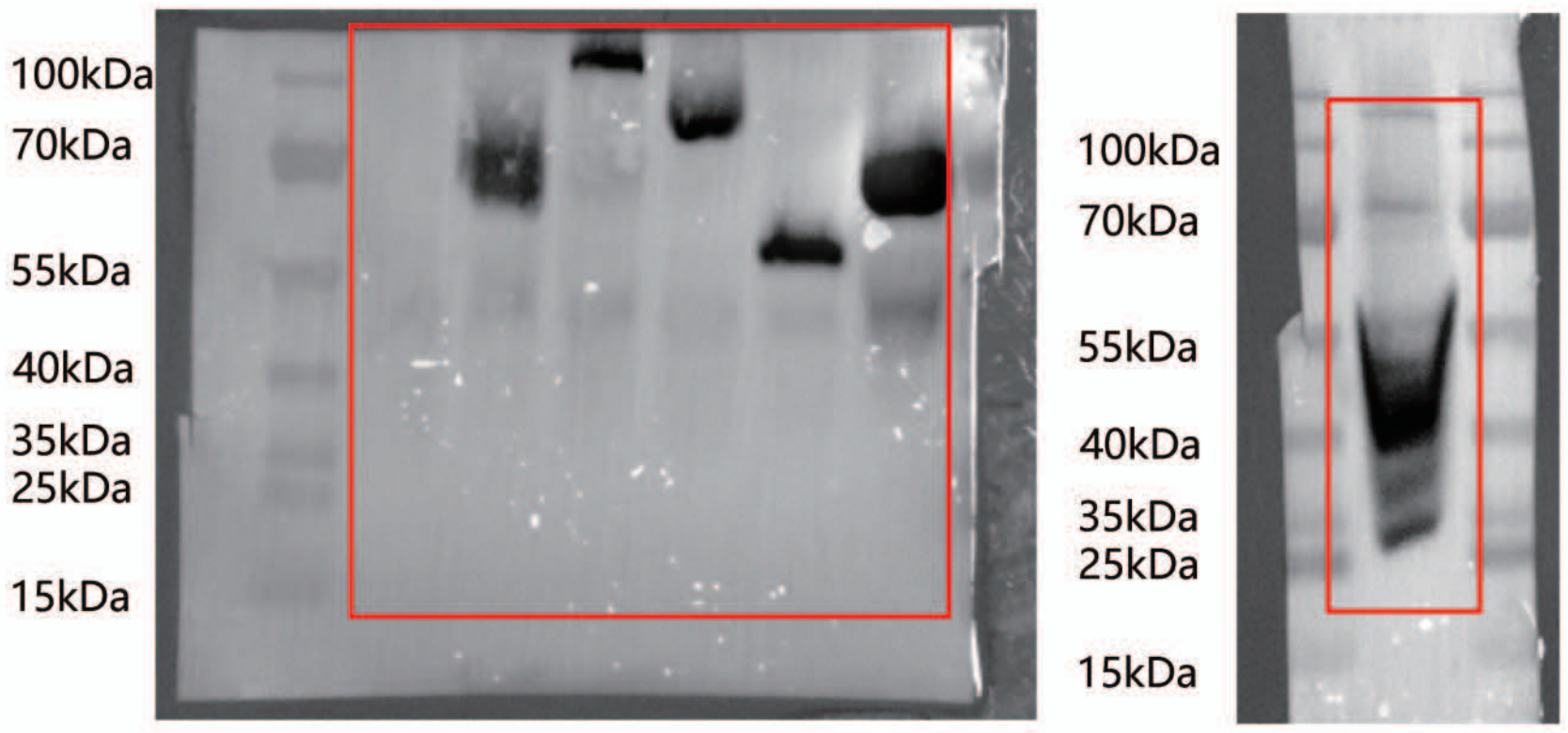

Fig. S8F

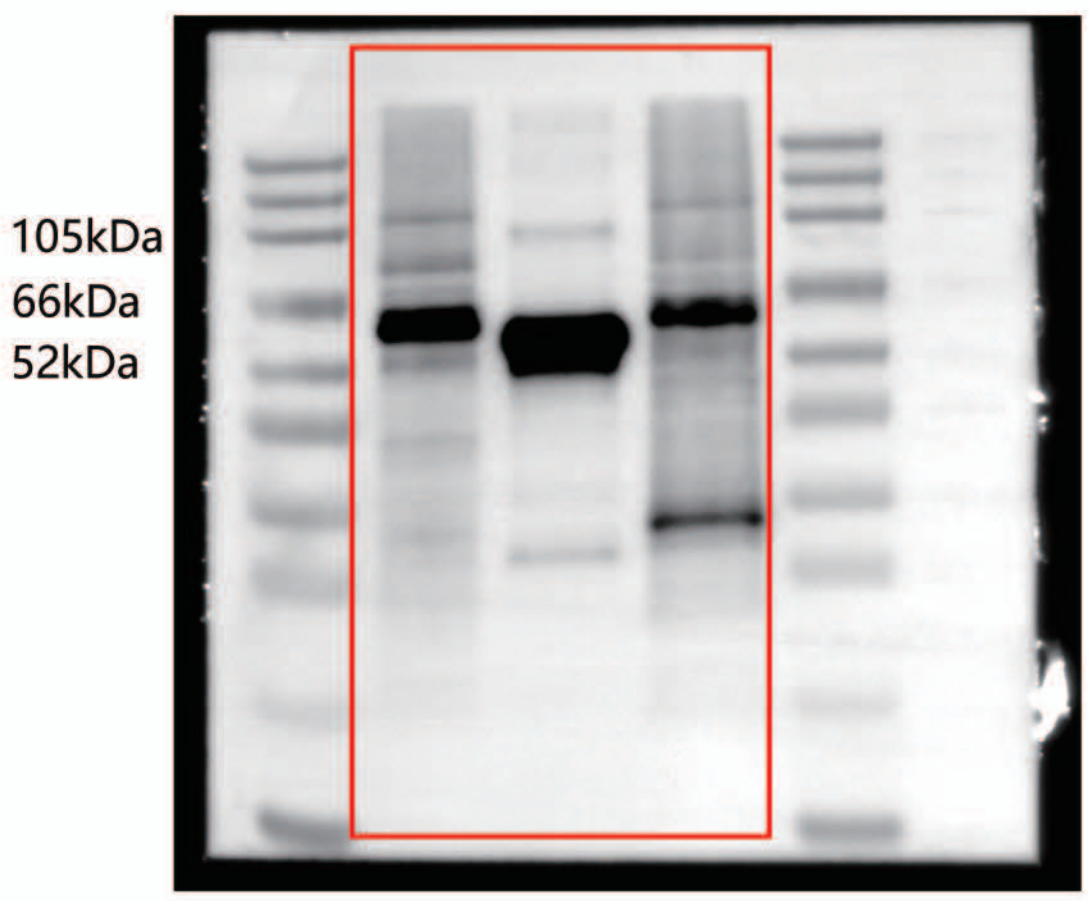

Fig. S8G

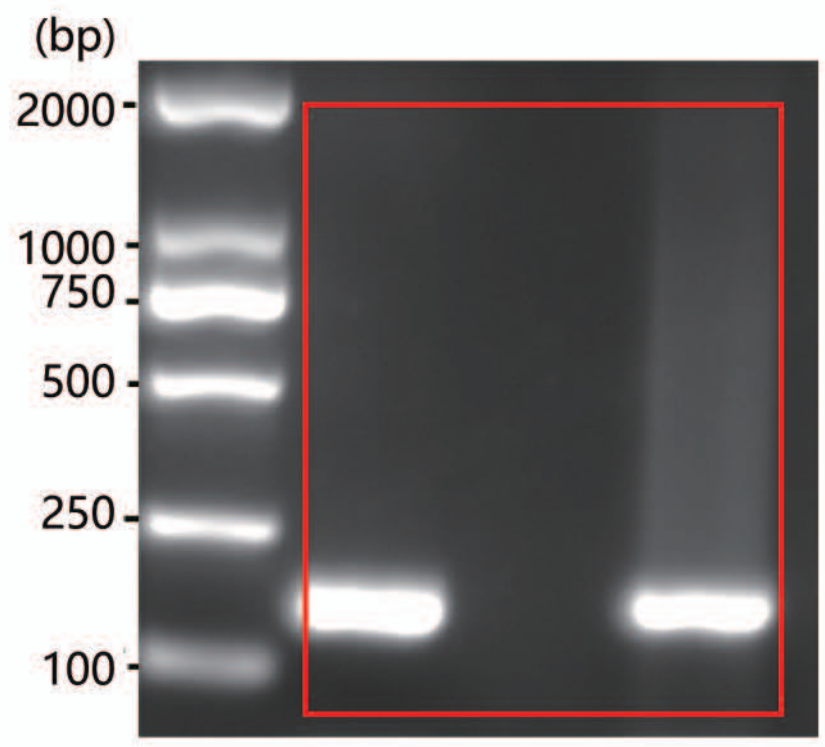

Fig. S10B

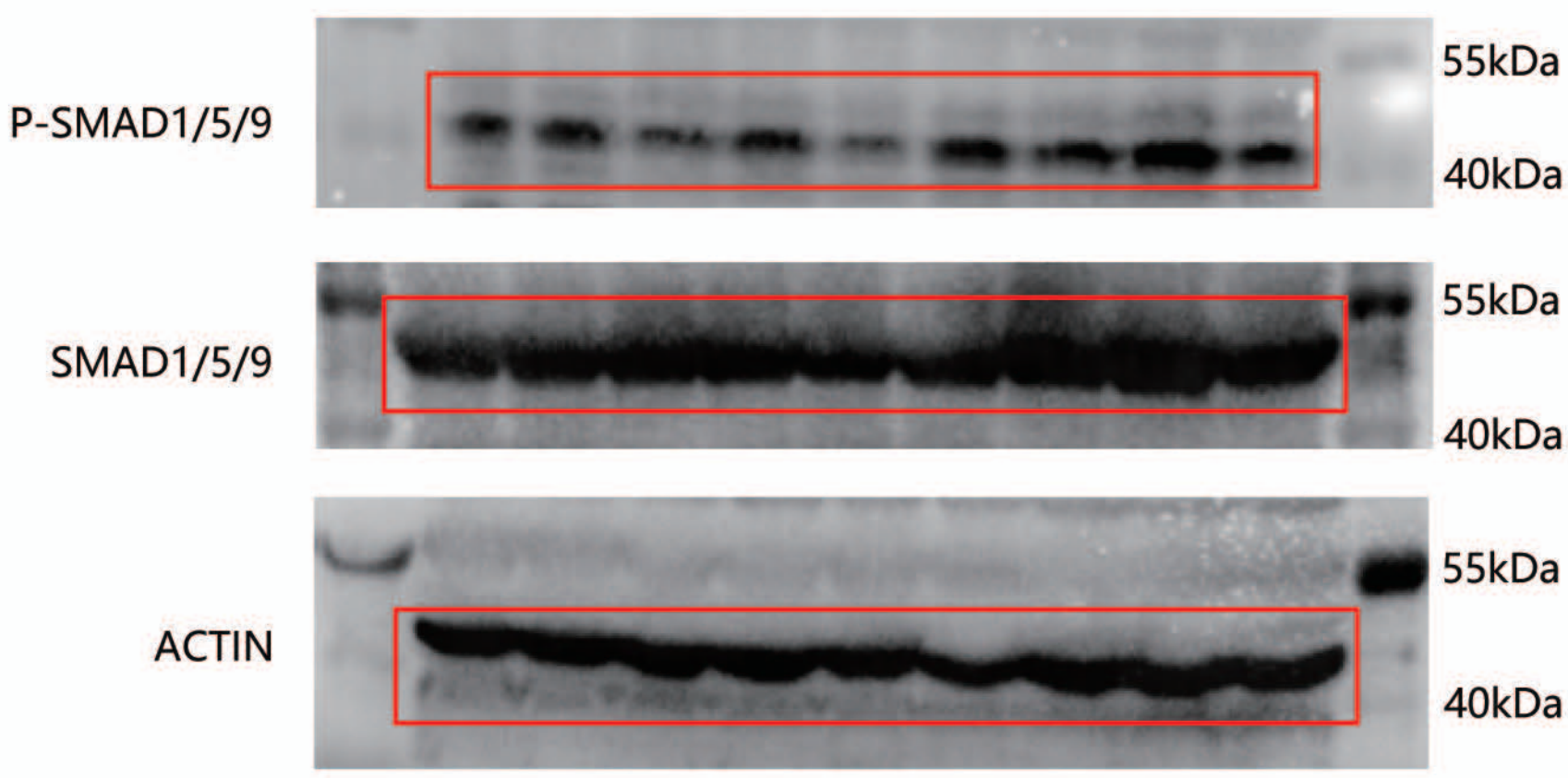

Supplement: Supplementary file 2 [file jci-136-207031-s130.pdf]
